# Optimizing Photogrammetry Parameters for 3D Reconstruction of Fresh Brain Specimens

**DOI:** 10.1007/s12021-026-09789-y

**Published:** 2026-07-25

**Authors:** Carlos A. Rueda-Pérez, María-Camila Valencia-Loaiza, Paula Vega-Cordoba, Juliana Tobón, Dylan S. Anaya, Andres Gonzalez-Leyton, Juan F. Mazo, Jesús D. Tarazona, Santiago Ruiz, Juan G. Martinez, Andrés Echeverri-Garcia, Catherine J. Gomez-Moreno, Brian Vicaño-Metaute, Johana Gómez-Ramirez, Andres Villegas-Lanau

**Affiliations:** 1 Grupo de Neurociencias de Antioquia, Universidad de Antioquia, Medellín, Colombia; 2 Facultad de Medicina, Universidad de Antioquia, Medellín, Colombia

**Keywords:** Photogrammetry, 3D brain reconstruction, Metashape, Neuroimaging

## Abstract

Photogrammetry has become an essential tool in medical and research fields for generating high-fidelity 3D models from 2D images. However, optimizing the imaging and processing parameters remains a challenge, particularly for fresh brain specimens, where repeated imaging is not feasible. This study systematically evaluates the impact of various Metashape alignment settings on 3D reconstruction outcomes, analyzing 12,600 configuration combinations and 63,000 alignments. Our results suggest that the optimal imaging setup consists of four photo sets: two captured at anatomical position (level with the brain and 30 cm above it at a 30° camera tilt) and two identical sets with the basal side facing upwards. The brain should be rotated 3° between shots, generating 120 images per set. Initial processing should be performed without masks using medium-precision alignment in Metashape. If alignment fails, we recommend generating one mask per image, delineating the brain’s borders, and applying masks to key points. Notably, higher image density only improves alignment reliability when masking is selected to detected features and may only increase processing time. We also observed variability in alignment results under identical conditions, suggesting an inherent stochastic component in Metashape. Consequently, unsuccessful alignments should be repeated before modifying imaging parameters. To our knowledge, this is the first study to systematically define an optimal imaging and processing parameters for 3D photogrammetry of fresh brains using a turntable. Future research should focus on determining the minimum number of images required to ensure high-quality reconstructions.

**Supplementary Information:**

The online version contains supplementary material available at https://doi.org/10.1007/s12021-026-09789-y.

## Introduction

Photogrammetry, originally developed for cartography and topographic mapping, has gained increasing relevance in biomedical research due to its capacity to generate highly realistic three-dimensional (3D) surface models from two-dimensional (2D) photographic data (Struck et al., 2019; Wesencraft & Clancy, 2019). This technique, underpinned by Structure-from-Motion (SfM) methodologies, is now being leveraged across a range of medical and neuroscientific applications, including the digital archiving of brain specimens (De Benedictis et al., 2018; Petriceks et al., 2018; Shintaku et al., 2019; Balzli et al., 2022; Oliveira et al., 2023; Titmus et al., 2023; Corvino et al., 2024; Piazza et al., 2025). Recent advances in computational photogrammetry software, such as Agisoft Metashape (Agisoft Metashape, Agisoft LLC, St. Petersburg, Russia), which offers a Python-based architecture for efficient data integration that have facilitated its adoption in laboratory settings.

Despite these advancements, unique challenges persist when applying photogrammetry to fresh ex vivo brain tissue, particularly within the constraints of brain banking workflows. At the Neurobank of the Neuroscience Group of Antioquia (GNA), photographs are taken from fresh brain specimens before further processing, which precludes repeated access to the intact structure. This protocol demands a highly efficient and reliable photographic workflow, as degradation of biomolecular and cellular structures can occur rapidly postmortem, influencing both the quality of tissue and the feasibility of advanced imaging modalities such as electron microscopy and immunohistochemistry (Krassner et al., 2023, Garrod et al., 2025, Olney et al., 2025). Notably, morphometric alterations (including changes in cell volume, oncosis, necrosis, and decomposition) can manifest within as little as three to four hours postmortem, underscoring the need for rapid sample handling (Koenig & Koenig, 1952; Badonic et al., 1992; Garcia et al., 1995; Solenski et al., 2002, Shepherd et al., 2009).

Moreover, the photogrammetric reconstruction of fresh brain specimens imposes additional constraints: the photographs captured represent the sole opportunity to document the tissue’s native state, as retakes are not possible after tissue processing commences. This stands in contrast to most published photogrammetry workflows, which typically rely on fixed tissue specimens that permit repeated imaging and iterative refinement (Shintaku et al., 2019; Nebel et al., 2020; Balzli et al., 2022; Cantizani-Oliva et al., 2023; Oliveira et al., 2023; Corvino et al., 2024; Hill et al., 2024). However, fixation is associated with significant and variable tissue shrinkage or swelling, with macroscopic changes in total brain or regional volumes reaching up to 60% in some animal models (Bahr et al., 1957; Del Signore et al., 2022; McKenzie et al., 2024; Yoshimaru et al., 2024). Such artifacts can confound morphometric analyses and limit the translational value of post-fixation photogrammetry.

A further challenge arises from the sensitivity of photogrammetric reconstruction to both user experience and software parameters. Although default or recommended settings in Agisoft Metashape are frequently adopted, these may not be optimal for the rapid, single-pass documentation required for fresh brain tissue in a brain banking context. In our experience at GNA, the configuration of SfM parameters during the “alignment” phase (Metashape’s terminology) is a critical determinant of model fidelity, yet the literature lacks systematic evaluation of these settings for fresh human brain specimens, particularly for workflows employing turntable-based imaging.

In this manuscript, we aim to address this knowledge gap by systematically evaluating how combinations of photogrammetry parameters influence the accuracy and reliability of 3D reconstructions from fresh ex vivo brain tissue. Our objective is to inform the development of an optimized, rapid photogrammetry workflow for brain banks, ensuring high-quality digital archiving while minimizing procedural errors and enhance the quality and utility of digital neuroanatomical archives. Ultimately, this work seeks to enhance the reproducibility and utility of neuroanatomical data within the field of neuroinformatics.

## Methodology

**Fig. 1:**
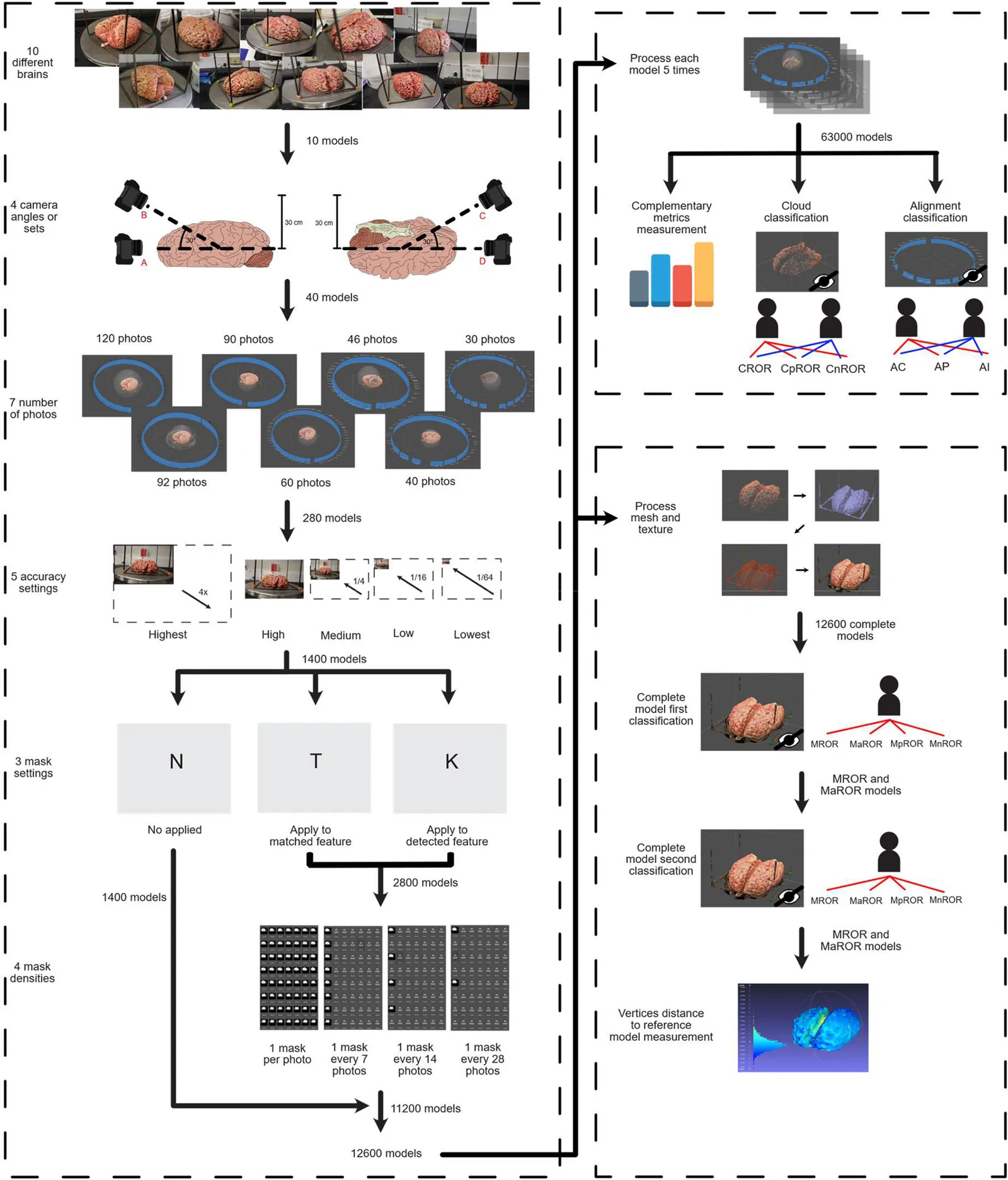
Workflow of the photogrammetry pipeline and experimental design. Ten brains were acquired and photographed using a turntable-based setup under four camera configurations (sets a–d), differing in angle and orientation. For each set, multiple photo densities were tested (30–120 images per 360°). SfM processing was performed in Metashape using different accuracy settings (highest to lowest), followed by masking strategies, where masks were applied either to detected features (key points) or matched features (tie points). Additionally, mask frequency per image was varied (from no masking to one mask per image). These parameter combinations generated 12,600 unique configurations, each rendered five times to account for stochastic variability, resulting in 63,000 models. Each model was independently evaluated twice for point cloud classification (CROR, CpROR, CnROR) and twice for alignment classification (AC, AP, AI), while complementary quantitative metrics were extracted. The original 12,600 configurations were further processed to generate dense clouds, meshes, and textures. These final models underwent a two-step qualitative evaluation, followed by quantitative assessment of vertex distance relative to a reference model

From our database, we selected 10 brains that had been photographed for photogrammetry. Images were acquired using a Canon EOS 60D (Canon Inc., Tokyo, Japan) with 21 MP resolution. A custom-built turntable rotated in 3° increments, completing a full revolution in 120 steps, with one photograph manually captured per step (Fig. 1). For each brain, four photo sets were obtained: two in anatomical position at the same level as the brain and two approximately 30 cm above the specimen with a camera inclination of ~ 30° (designated sets A and B). The remaining two sets used the same configurations but with the basal surface facing upward (sets D and C) (Fig. 1). Each brain therefore yielded approximately 480 photographs (~ 120 per set). Images were imported into Metashape, where one chunk per set was created to generate individual models.

**Fig. 2:**
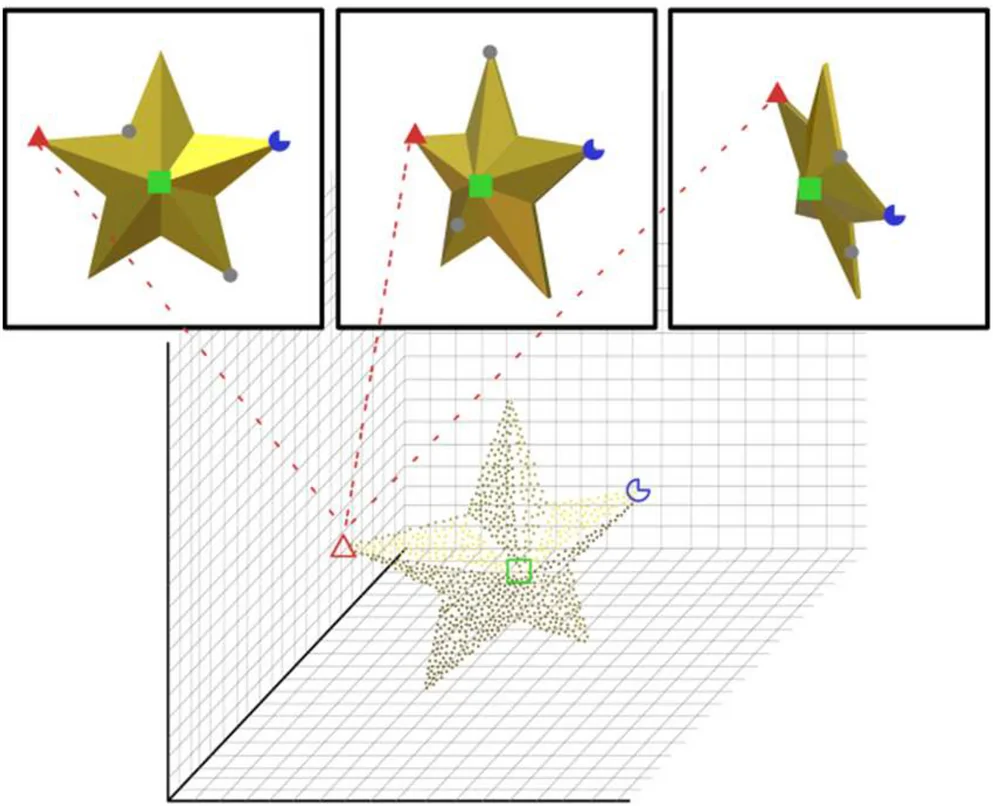
Each photograph (represented as squares with a star in the upper half) contains multiple points of interest, known as key points (gray circles). The software identifies when the same key point is present in multiple photographs, linking them together as tie points (solid red triangle, green square, and blue pie). The spatial coordinates of these tie points are then calculated in three-dimensional space (hollow red triangle, green square, and blue pie), forming a point cloud that approximates the shape of the reference object, referred to as the tie point cloud

Metashape generates 3D models through a SfM pipeline. The Photo Alignment stage, our primary focus, detects distinctive image features (“key points”) and matches them across photographs to create matched features (“tie points”), which form the sparse point cloud (Fig. 2) (Carrivick et al., 2016; Luhmann et al., 2020). Alignment quality can degrade when images lack texture or are blurred (Burk & Johnson, 2019; Oliveira et al., 2023), and the literature strongly recommends multi-angle acquisition to reduce blind spots (De Benedictis et al., 2018; Nebel et al., 2020; Gurses et al., 2021; Corvino et al., 2024).

We hypothesize that several camera-related factors influence SfM outcomes. Camera translation and rotation are interdependent, and preliminary testing suggested that configurations capturing a larger visible brain surface produce more consistent reconstructions, likely because the software detects more key points. For example, images captured at 0° tended to be less accurate than those at 30°, probably because the latter include both frontal and parietal surfaces. To evaluate angular effects, all combinations of sets A–D will be analyzed, comparing same-level images (A vs. D) with angled images (B vs. C).

Photo density was also investigated. Although greater coverage might improve reconstruction, Agisoft recommends ~ 60% overlap in aerial photogrammetry, suggesting a possible saturation point beyond which additional images mainly increase processing time (Agisoft, 2024). Because no specific guidelines exist for turntable-based objects, we will test densities of 120, 92, 90, 60, 46, 40, and 30 photos per 360° (Fig. 1). Configurations of 120, 90, 60, 40, and 30 use uniform spacing, whereas 92 and 46 increase sampling at the frontal and occipital poles. Preliminary observations indicated that poles are harder to align than lateral regions, possibly due to reduced surface area; thus, increased pole density may improve results. Qualitative correlation analyses will first evaluate relationships between density and subjective cloud quality, followed by comparisons between constant-density (90, 40) and variable-density (92, 46) groups. Rendering time will also be analyzed.

**Fig. 3:**
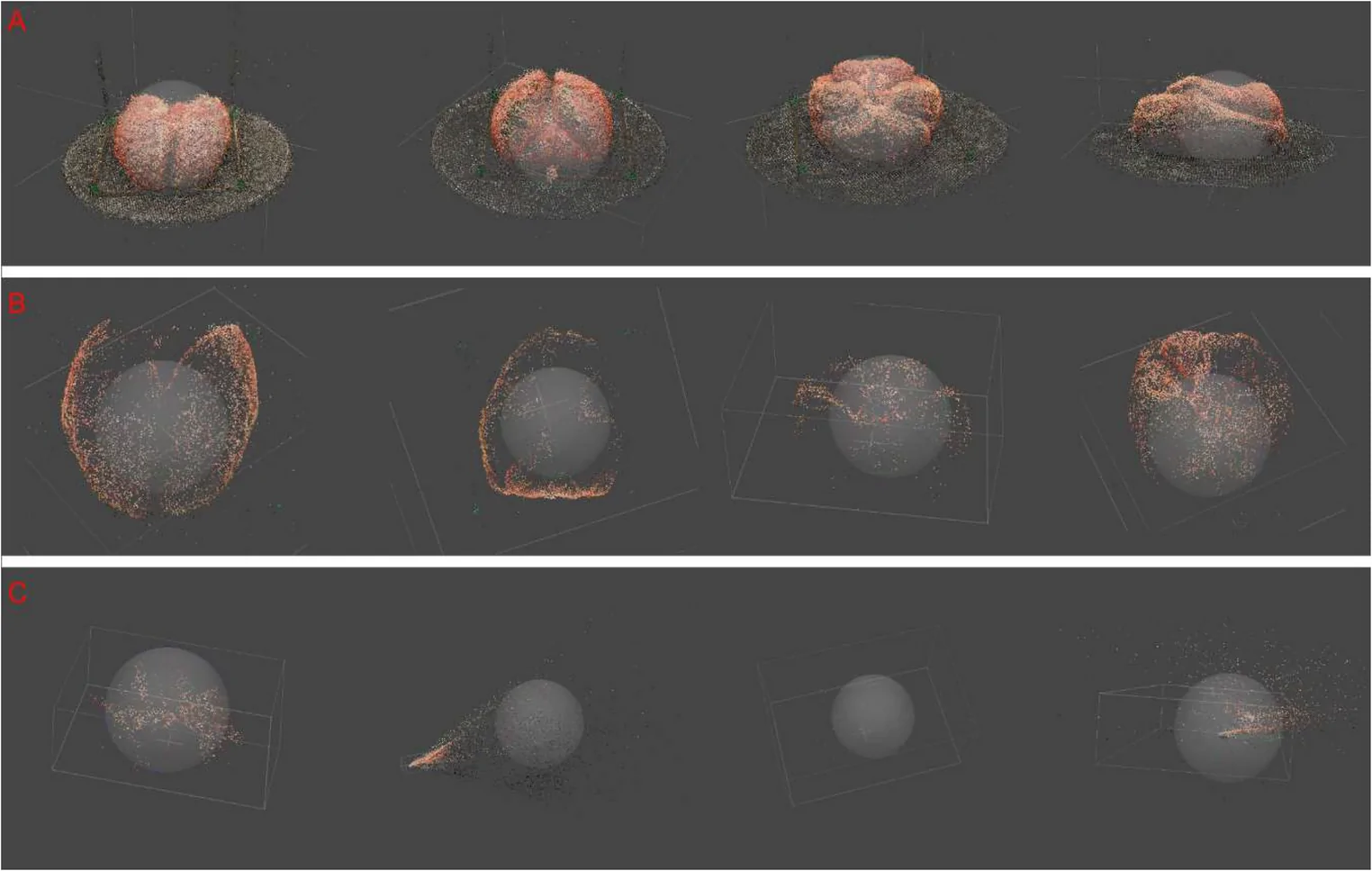
Examples of Brain Tie Point Clouds. Row (**a**) displays Clouds that Represent Objects of Reference (CROR), where all lobes are visible with nearly uniform density. Row (**b**) presents Clouds that *Partially* Represent Objects of Reference (CpROR); note that while some areas of the brain are identifiable, others exhibit lower density. Row (**c**) features Clouds that do *Not* Represent Objects of Reference (CnROR), where no recognizable shape is discernible

Based on preliminary qualitative assessment, sparse clouds can be classified as CROR, CpROR, or CnROR (Table 1; Fig. 3). Empirically, CnROR clouds rarely yield acceptable models despite parameter tuning, whereas CROR clouds typically reconstruct successfully. CpROR clouds show two possible behaviors: (1) incomplete but partially high-quality models, or (2) improved completeness after realignment without resetting prior results.

**Fig. 4:**
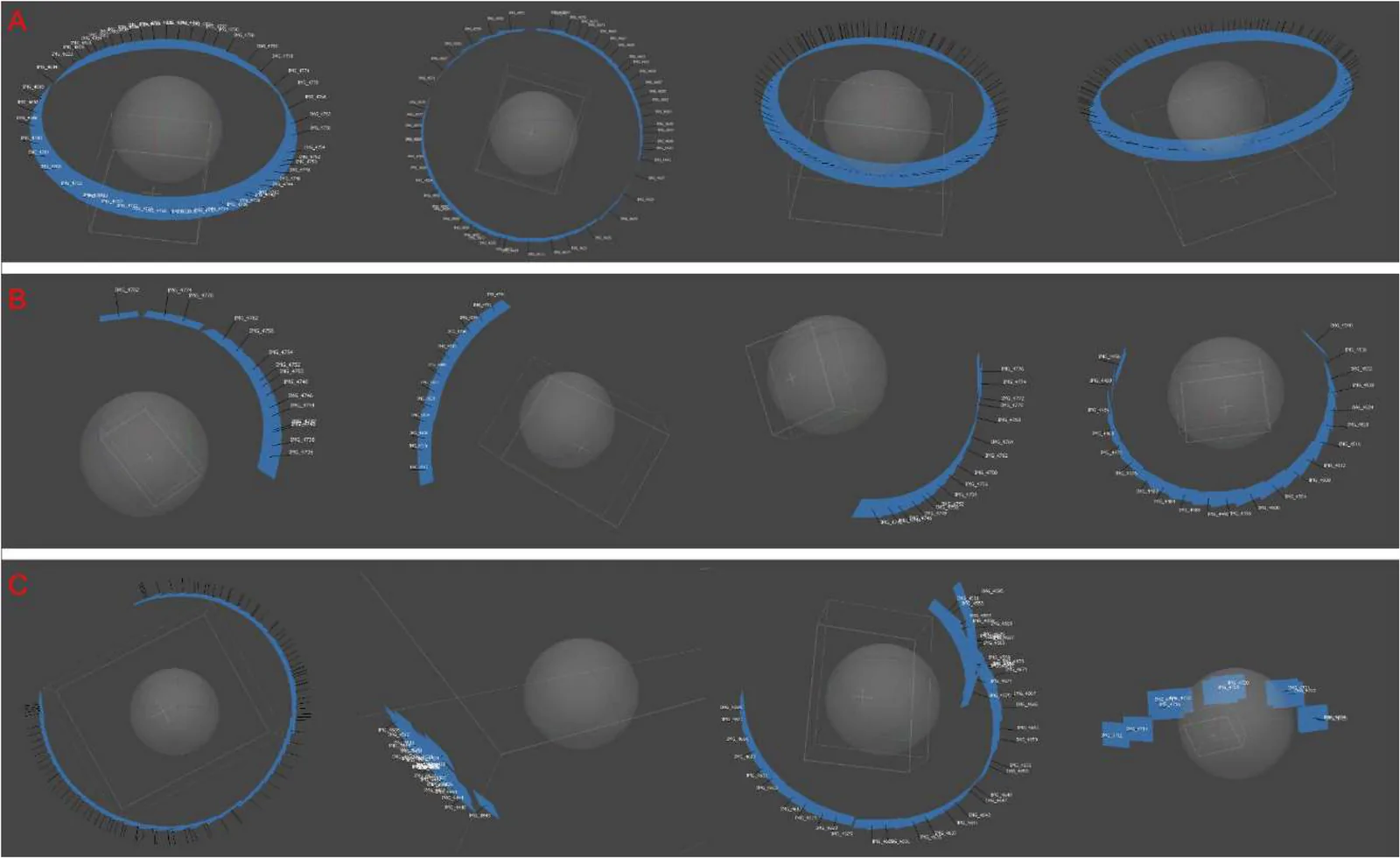
Examples of Brain Photo Alignment. Row (**A**) illustrates perfect circumferences formed by all photos, categorized as Correct Alignments (AC). Row (**B**) displays arcs of a circumference, identified as Partial Alignments (AP). Row (**C**) presents formations that deviate from circumferences or their arcs, classified as Incorrect Alignments (AI)

We also observed that camera alignment patterns provide predictive information. Optimal alignments form a crown-like camera cluster,

allowing classification into Complete (AC), Partial (AP), or Incorrect (AI) alignment (Table 2; Fig. 4).

**Table 1:** Evaluation criteria for brain tie point cloud

Evaluation	Criteria
CROR	Mandatory: • All lobes of the reference object are visible. • The density of the point cloud is consistent across the different lobes • No noticeable deformities are present when compared to the reference object. Optional (at least one must be met): • The dense cloud is symmetrical with respect to the sagittal plane of the model. •Any asymmetry in the sagittal plane is attributable to the acquisition of tie points rather than their absence when compared to the reference object. Furthermore, the asymmetry must result in low-density points and should not cause significant distortion of the reference object. These points may be eliminated during the dense cloud or mesh-building stages.
CpROR	Mandatory: • At least one complete lobe of the model is identifiable. •No noticeable deformities are present when compared to the reference object. Optional (at least one must be met): • The dense cloud exhibits asymmetry with respect to the sagittal plane of the model. The density of the cloud is inconsistent across its different lobes.
CnROR	Optional (at least one must be met): • No lobes of the reference object are identifiable. • There is a noticeable deformation compared to the reference object that is not due to the addition of tie points or results in high-density artifacts.

We hypothesize correspondence between AC and CROR, AP and CpROR, and AI and CnROR, and further propose that alignment quality may predict final model success more reliably than cloud inspection alone.

Because preliminary tests revealed stochastic variability, each configuration will be rendered five times. Quantitative variability will be measured via SD. Qualitative variability will use a scoring system combining alignment and cloud ratings (0–4 scale), with SD thresholds defining consistent (0), minimal (> 0–0.6), and high (> 0.6) variability.

For qualitative analysis, anonymized models will be independently evaluated by two raters, trained medical students, on cloud-only and alignment-only views (Fig. 1). Concordant ratings will be used for statistics. Associations between alignment and cloud quality will be assessed using χ² tests, Cramer’s V (Akoglu, 2018 thresholds), and adjusted standardized residuals (cutoff ≥ 0.01). This set of tests constitutes the qualitative correlation statistical battery.

Pipeline performance will be quantified using multiple complementary metrics: (i) number of matched features; (ii) Root Mean Square (RMS) reprojection error per matched feature; (iii) median of valid projections per matched feature; (iv) median of valid projections per image; (v) median total projections per image; (vi) valid/total projection ratio and (vii) total processing time (feature matching + alignment). Quantitative data will be extracted using custom Python scripts built on the Metashape API.

Quantitative comparisons will use t-tests and violin plots. Subplot matrices will highlight nonsignificant comparisons (red connectors), with horizontal lines representing within-group tests and vertical lines representing between-group tests. Outliers will be removed using the 1.5×IQR rule, and Bonferroni correction (m = 2898) will control the family-wise error rate .

To evaluate downstream effects, the full photogrammetry pipeline will be completed (low-quality depth maps, reduced meshes, 4096-px textures).

**Table 2:** Evaluation criteria for brain photo alignment

Evaluation	Criteria
AC	Mandatory: • The aligned photos form a perfect circle. • All photos in the set are aligned. • There are no gaps in the circle larger than the size of two cameras. • There is no clustering of more than two cameras. • The focus of the camera normals points to the center of the circle.
AP	Mandatory: • There is no clustering of more than two cameras. • The focus of the camera normals points to the center of the circle. Optional (at least one must be met): • The aligned photos form an arc of a circle. • There are two or more arcs that belong to the same circle.
AI	Optional (at least one must be met): • No arc or circle is formed. • The formed arc is not circular. • At any point along the arc or circle, there is a clustering of more than two cameras. • The focus of the camera normals does not point to the center of the circle. • There is a change in the focal point of the camera normals at any part of the arc or circle. • The alignment forms a circular arc despite all photos being aligned. • There are two or more arcs belonging to different circles.

**Fig. 5:**
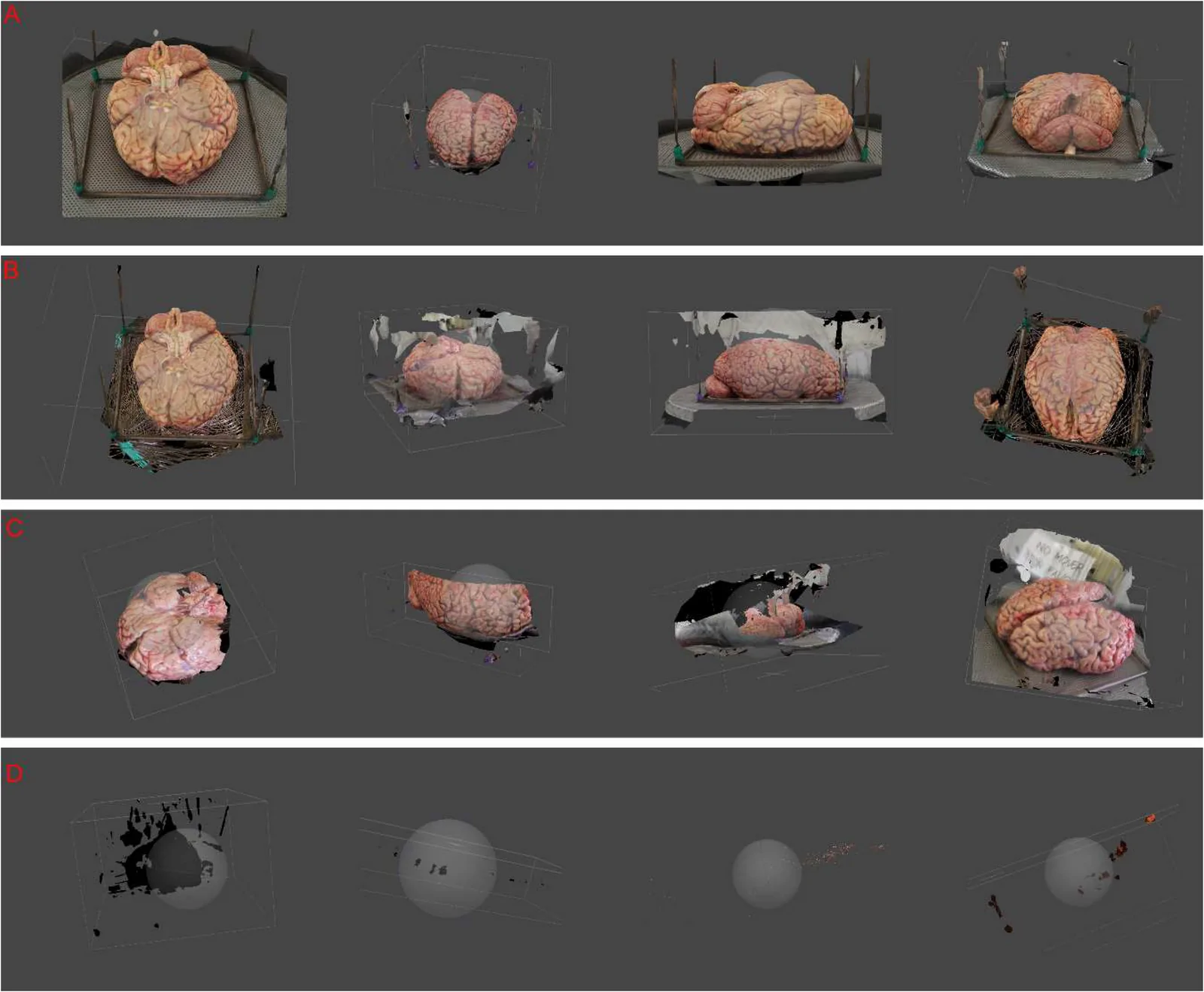
Examples of complete 3D reconstruction of photogrammetrically captured brains. Row (**A**) illustrates Models that Represent the Object of Reference (MROR), showcasing a complete and accurate representation of the entire brain and custom scale (green and purple dots). Row (**B**) features Models that *Approximately* Represent the Object of Reference (MaROR), where a complete brain and custom scale are rendered, but unwanted noise is also present. Row (**C**) displays Models that *Partially* Represent the Object of Reference (MpROR), in which some parts of the brain are recognizable, but the model is incomplete or deformed. Row (**D**) depicts Models that do *Not* Represent the Object of Reference (MnROR), where no part of a brain is identifiable

Final models will be classified as MROR, MaROR, MpROR, or MnROR (Table 2; Fig. 5). One co-author will perform the primary evaluation, and an experienced author (C.A.R.) will confirm MROR/MaROR cases (Fig. 1). Four contingency tables (separate and merged MROR/MaROR) will relate alignment and cloud quality to final model outcome, with the table showing the highest Cramer’s V prioritized for subsequent analyses.

RMS distance to a manually generated reference model will be measured on these complete models using MeshLab’s “Align” and “Distance from reference Mesh” functions (Cignoni et al., 2023).

## Results

Ten brains, donated between 2020 and 2021, were selected for photogrammetry imaging, with an intended goal of capturing 120 photos per set. A total of 4,541 photos were taken across 40 sets, yielding a mean of 117 photos per set with a standard deviation of 13.88. The smallest set comprised 79 photos, while the largest included 141. Each photo was manually masked. In total, the alignment process was rendered for 63,000 models, of which 12,600 underwent the full photogrammetry pipeline to generate dense point clouds, meshes, and textures.

**Table 3:** Evaluation Criteria for complete 3D reconstruction of photogrammetrically captured brains

Evaluation	Criteria
MROR	Mandatory: • From all perspectives, the model accurately represents a brain with a complete custom scale, and there are no deformities or a missing base.
MaROR	Mandatory: • From all perspectives, the model represents a brain with a complete custom scale; however, there are abstractions present at the base.
MpROR	Optional (at least one must be met): • The model resembles a brain; however, there are deformities in the custom scale, or it is incomplete. • From the predetermined viewpoint, the model represents the object of reference, but from other perspectives, it appears incomplete or deformed.
MnROR	Optional (at least one must be met): • There is no complete model, either due to missing texture creation, absence of a mesh, or lack of tie point alignment. • The model does not resemble a brain from any point of view. • The model contains an additional structure that is not present in the object of reference. • From the predetermined viewpoint, the model contains errors when compared with the object of reference.

The mean duration of the alignment process was 1.07 min (mins), with a median of 2.73 min and a standard deviation of 5.27 min. The minimum duration recorded was 0.06 min, with the first quartile at 0.48 min and the third quartile at 2.53 min. The longest alignment process took 393.15 min.

### Point Cloud Evaluation

In the qualitative evaluation of 63,000 tie point clouds, the two evaluators agreed on the classification of 43,099 models (68.41%). Among these, 14,020 were classified as CROR (22.25%), 4,165 as CpROR (6.61%), and 24,914 as CnROR (39.54%). For the remaining models with disagreement, 5,616 were classified as CROR/CpROR (8.91%), 5,315 as CROR/CnROR (8.43%), and 8,967 as CpROR/CnROR (14.23%).

**Fig. 6:**
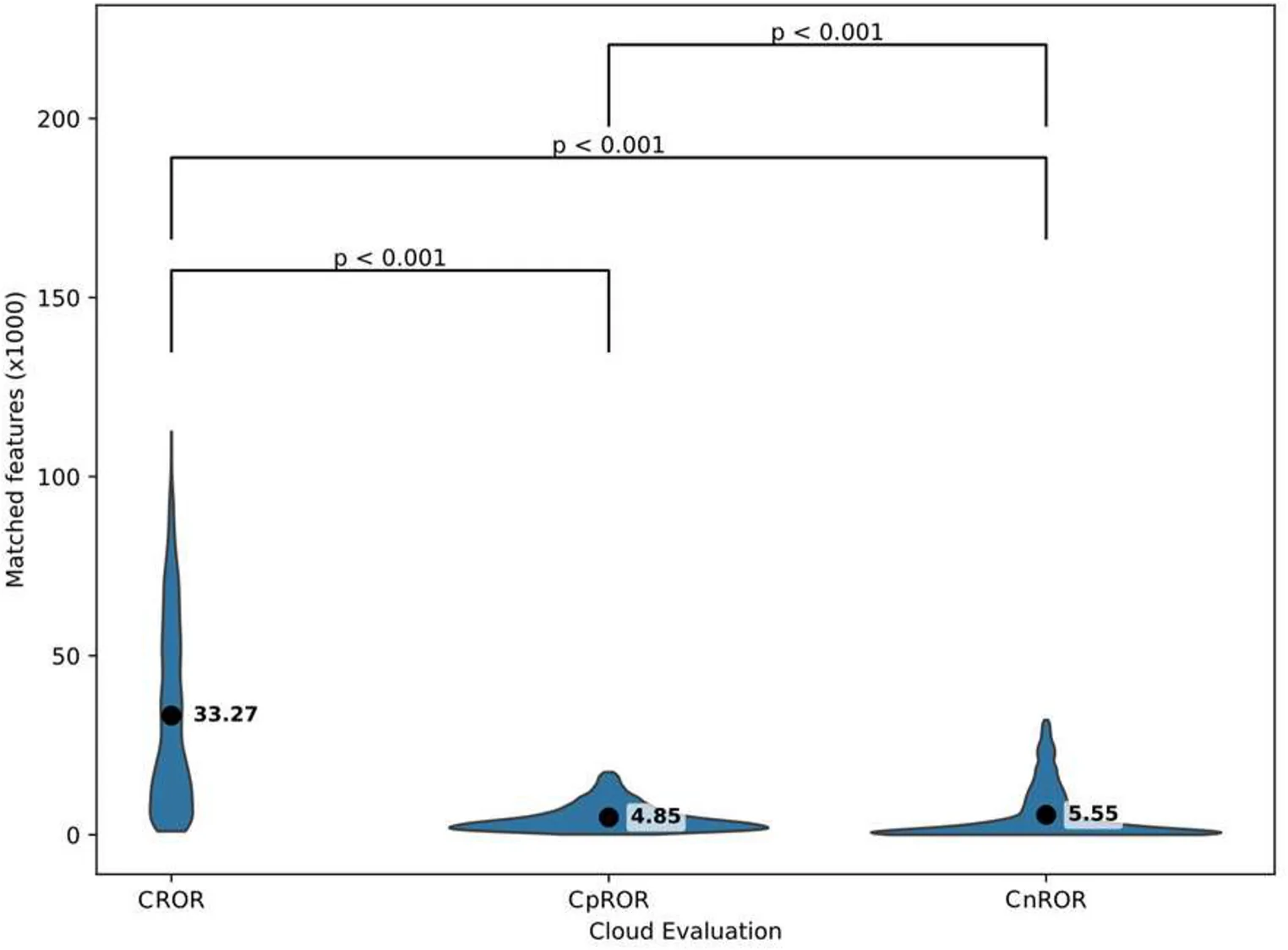
Matched features (x1000) by cloud classification. CROR models showed markedly higher numbers of matched features (mean: 33,271; median: 27,619; SD: 25,387; range: 972–112,548) compared with CpROR (mean: 4,850; SD: 3,786) and CnROR (mean: 5,546; SD: 7,407). Although CpROR and CnROR had similar mean values, CnROR exhibited a higher maximum (32,026 vs. 17,487) and a substantial proportion of models with zero matched features, whereas CpROR maintained a minimum of 95 matched features

**Fig. 7:**
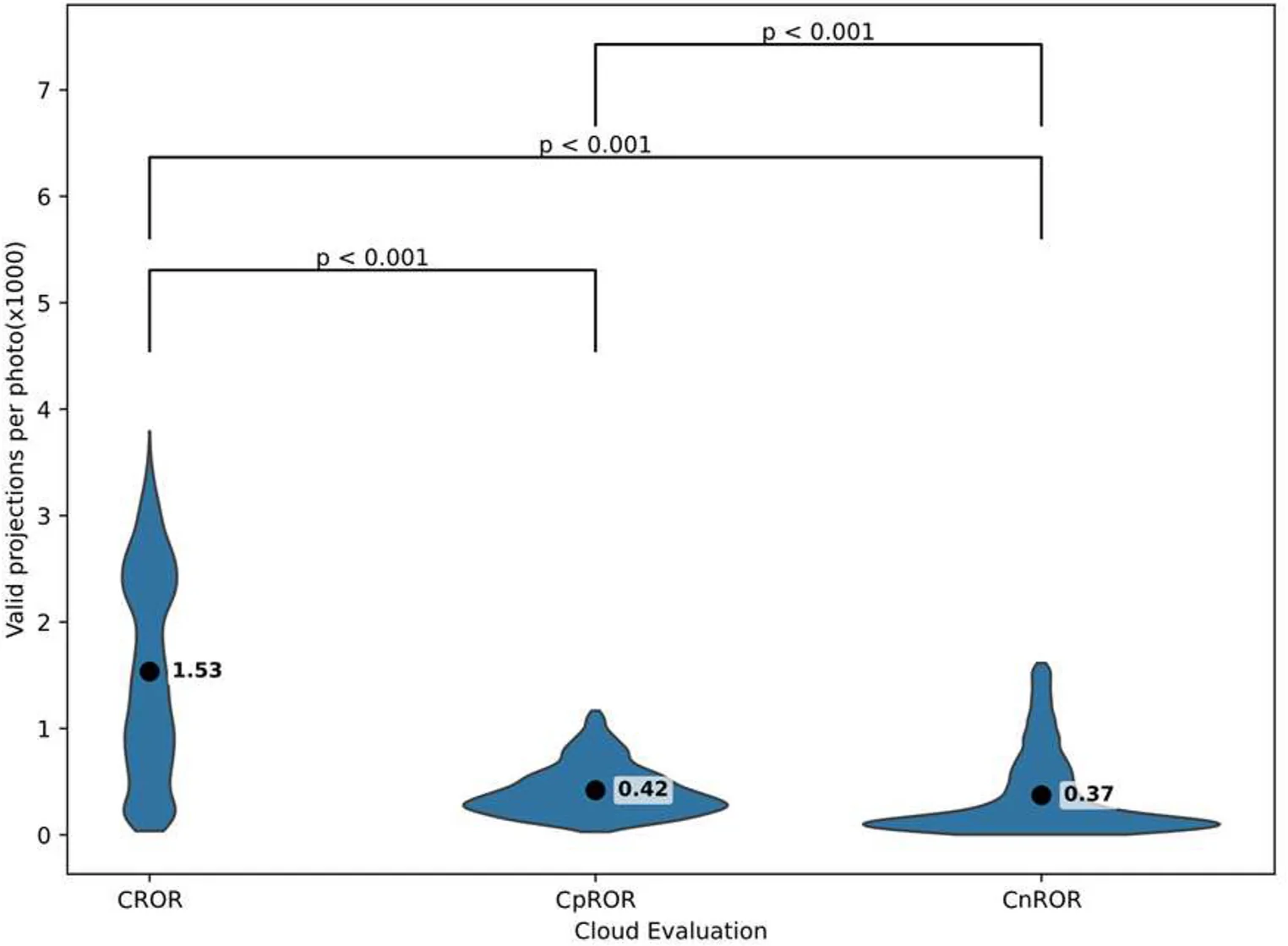
Figure 7 Valid projections per photo (x1000) by cloud classification. CROR models exhibited the highest number of valid projections per photo (mean: 1,534; median: 1,436; SD: 940), while CpROR and CnROR showed lower and narrower distributions (means: 419 and 372, respectively). A similar trend was observed for total projections per photo, with CROR showing the highest values and greatest variability

Most quantitative metrics differed significantly across CROR, CpROR, and CnROR groups (Supplementary Figure S1–S6), with the strongest separation observed in feature density and projection support. CROR models showed substantially higher numbers of matched features (~ 33k) compared with CpROR and CnROR (~ 4–6k) (Fig. 6). This distinction was reinforced by projection metrics (Fig. 7), where CROR also exhibited the highest number of valid projections per photo (~ 1,534 vs. ~400) and similarly higher total projections (Supplementary Figure S4).

In contrast, CpROR and CnROR displayed reduced and more variable feature support, with CnROR including many models with no matched features.

Processing time also differed across groups (Fig. 7), with CROR showing intermediate and stable durations (~ 1.5 min), CpROR exhibiting longer and more variable times (~ 2.1 min), and CnROR the shortest (~ 1.0 min). Error metrics provided complementary insights: RMS point error per matched feature was similar between CROR and CpROR but markedly higher and more variable in CnROR (Supplementary Figure S2). The valid-to-total projection ratio was highest in CpROR and slightly lower in CROR, while CnROR showed greater variability (Supplementary Figure S5), indicating that this metric alone does not reflect reconstruction quality.

**Fig. 8:**
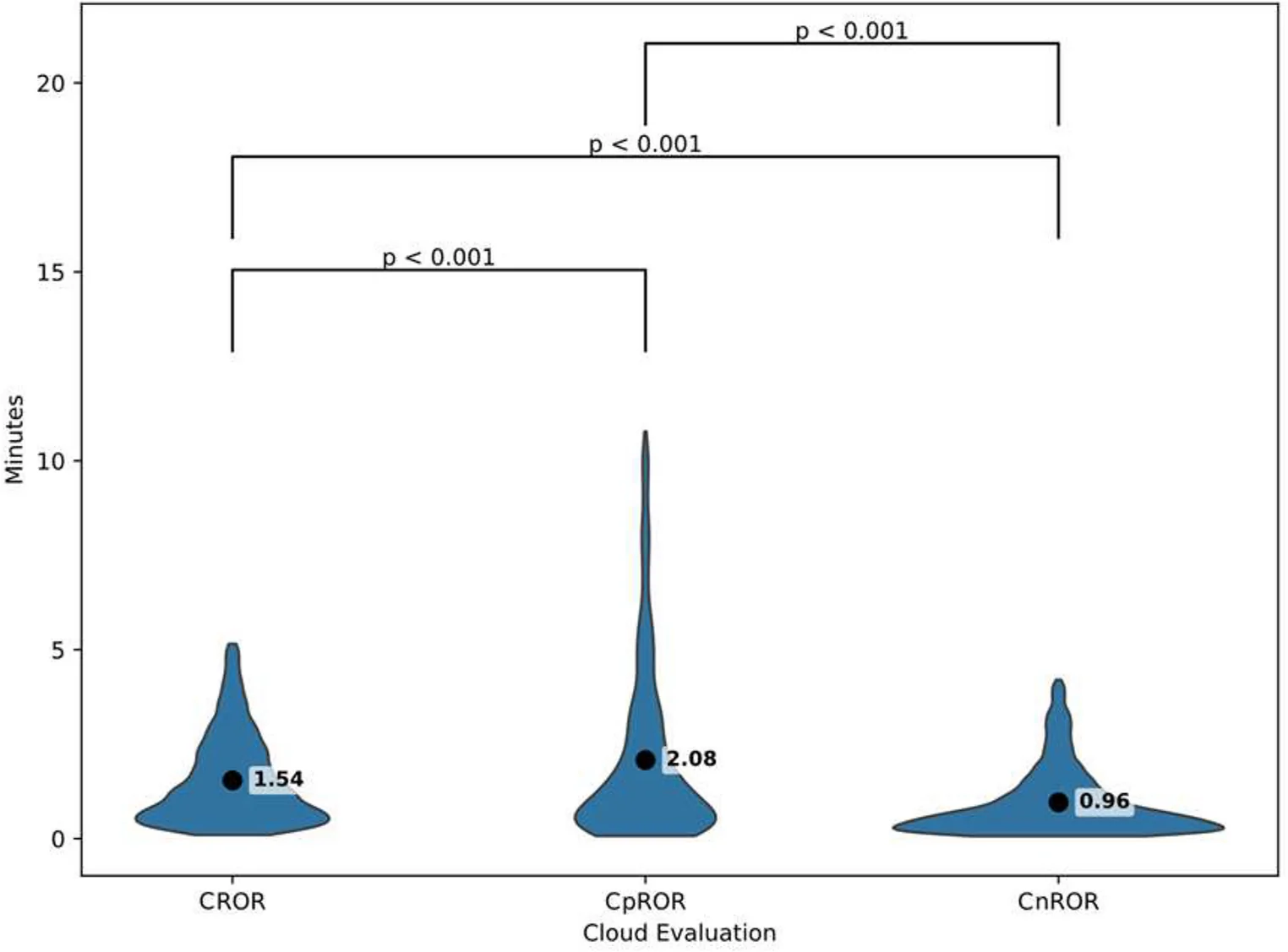
SfM processing time in minutes by cloud classification. Processing time varied across groups, with CROR averaging 1.54 min (median: 1.21), CpROR showing greater variability (mean: 2.08 min; SD: 2.29), and CnROR exhibiting the shortest times (mean: 0.96 min)

Importantly. final geometric accuracy, (RMS vertex distance to the reference model) did not differ significantly across groups, (*p* = 1), (Supplementary Figure S6), with similar central values for CROR and CpROR and a narrower distribution in CnROR due to its small sample size. Overall, these results indicate that CROR models are characterized by substantially higher feature density and projection support, although these advantages do not necessarily translate into measurable differences in final geometric accuracy.

### Alignment Evaluation

Of 63,000 alignments, the assessments of the two evaluators agreed on 45,304 models (71.91%). 11,041 models were classified as AC (17.52%), 2,425 as AP (4.28%), and 31,567 as AI (50.11%). For the remaining models with disagreement, 2,979 were classified as AC/AP (4.72%), 7,031 as AC/AI (11.16%), and 7,682 as AP/AI (12.19%).

Most quantitative metrics differed significantly across alignment categories (AC, AP, AI; *p* < 0.001), with the clearest separation observed in feature density and alignment performance (Supplementary Figures S7–S16). AC models showed the highest number of matched features (~ 33k), whereas AP had the lowest (~ 2.3k) and AI intermediate values (~ 7.8k), often including models with no matched features. This was consistent with alignment quality, where AC achieved perfect photo alignment (ratio = 1.0), AP showed poor alignment (~ 0.44), and AI displayed a broad and unstable distribution (~ 0.74). Similarly, AC models exhibited the highest number of valid projections per photo (~ 1,446), followed by AI (~ 475) and AP (~ 289), with comparable trends for total projections (See Supplementary Information).

Error and efficiency metrics further distinguished the groups. RMS point error per matched feature was similar between AC and AP (~ 2 mm), but substantially higher in AI (~ 3.75 mm), indicating poorer reconstruction consistency in misaligned cases (See Supplemental material). SfM processing time was longest and most variable in AP (~ 4.1 min), whereas AC (~ 1.6 min) and AI (~ 1.1 min) were faster and more stable. The valid-to-total projection ratio was highest in AP and lower in AC, while AI showed the greatest variability (See Supplemental Material), suggesting limited utility of this metric alone for assessing alignment quality.

Despite these differences in intermediate metrics, final geometric accuracy (RMS vertex distance to reference model) did not differ significantly across alignment categories (*p* = 1; SI). Overall, AC alignments are characterized by substantially higher feature density and more stable reconstructions, while AP and AI reflect degraded or inconsistent alignment performance, without a clear impact on final geometric accuracy.

### Point cloud vs. Alignment

**Fig. 9:**
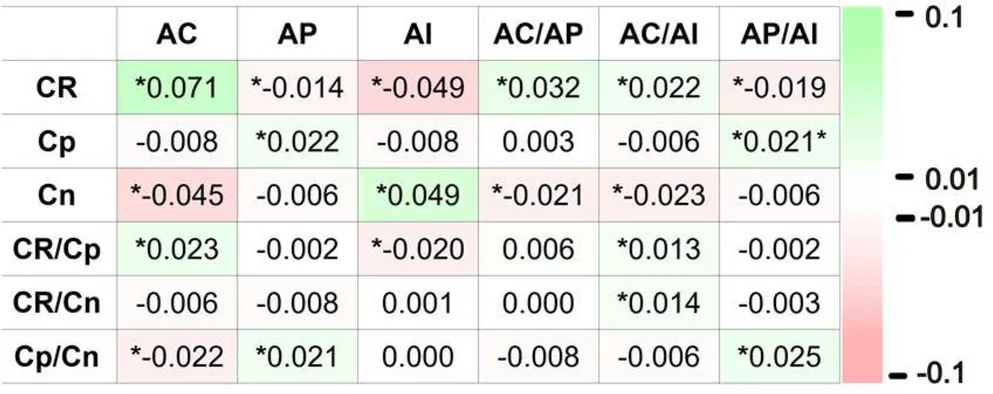
Adjusted standardized residuals of correlations between brain photo alignment evaluations and brain tie point cloud evaluation. Asterisks indicate significant values. p < 0.001. Cramer’s V = 0.31

When comparing the correlation between the subjective alignment evaluation and the subjective point cloud evaluation (Fig. 9), the χ² test indicated a statistically significant relationship (*p* < 0.001), with a very strong correlation (Cramer’s V = 0.3141). The comparisons that contributed the most to the correlation included AC and CROR (0.071), ACAP and CROR (0.032), and AI and CnROR (0.049), which were directly proportional. In contrast, AI and CROR (-0.049) and AC and CnROR (-0.045) were inversely proportional Fig. 9.

### Complete Model Evaluation

Of the 12,600 final models, 1,571 were initially classified as MROR (12.46%), 1,260 as MaROR (10.00%), 4,117 as MpROR (32.67%), and 5,652 as MnROR (44.85%). When MROR and MaROR were combined, they accounted for 2,831 models (22.46%). Following a second evaluation performed by a more experienced author, 600 models originally classified as MROR or MaROR were reclassified as MpROR. This resulted in 1,399 MROR models (11.10%), 832 MaROR models (6.60%), 4,717 MpROR models (37.43%), and 5,652 MnROR models (44.87%). The combined MROR/MaROR group thus comprised 2,231 models (17.70%).

**Fig. 10:**
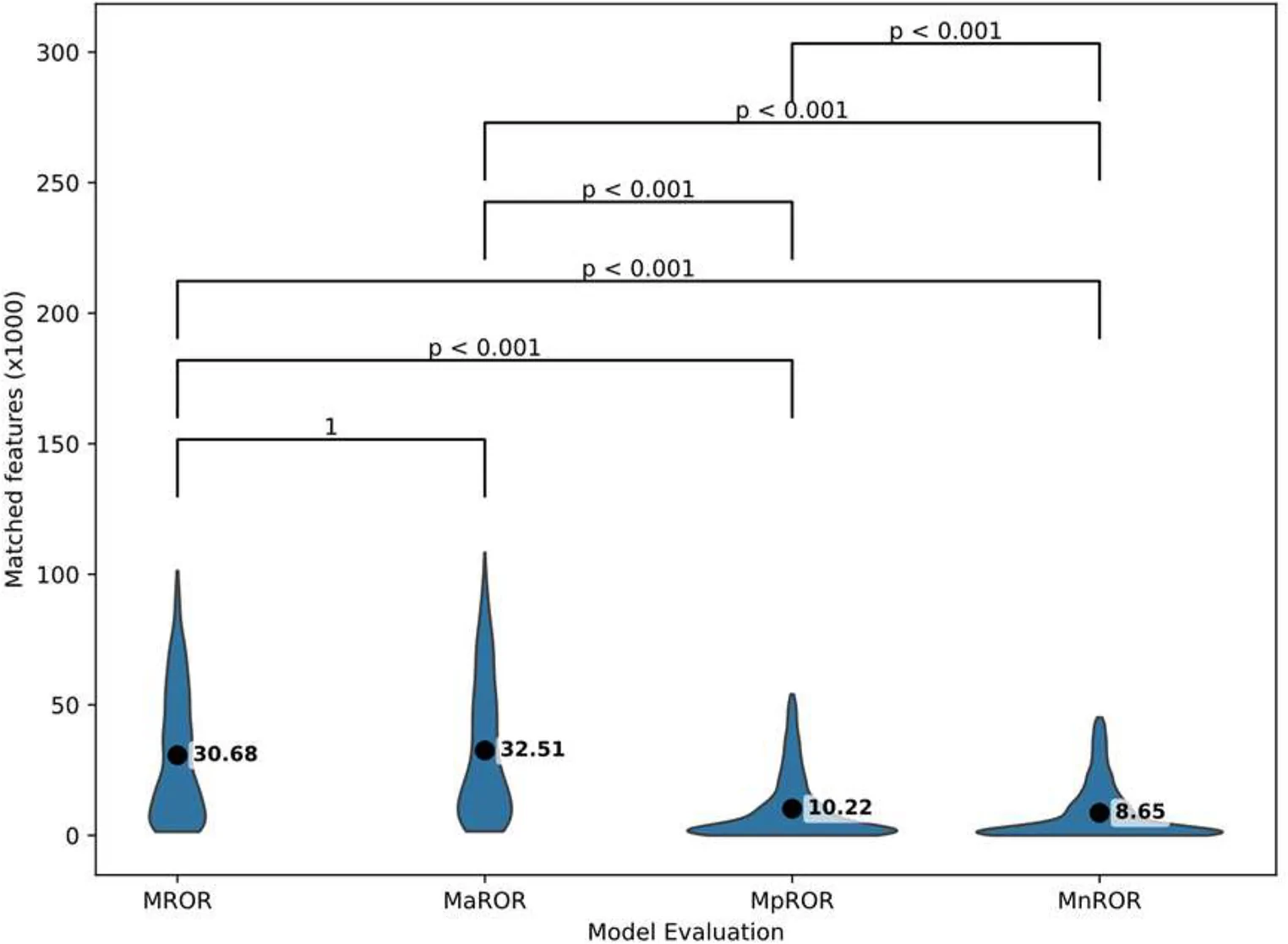
Matched features (x1000) by model classification. The number of matched features was similar between MROR (mean: 30,677; SD: 24,359) and MaROR (mean: 32,510; SD: 25,759; p = 1), but both were significantly higher than MpROR (mean: 10,216) and MnROR (mean: 8,648; all p < 0.001)

**Fig. 11:**
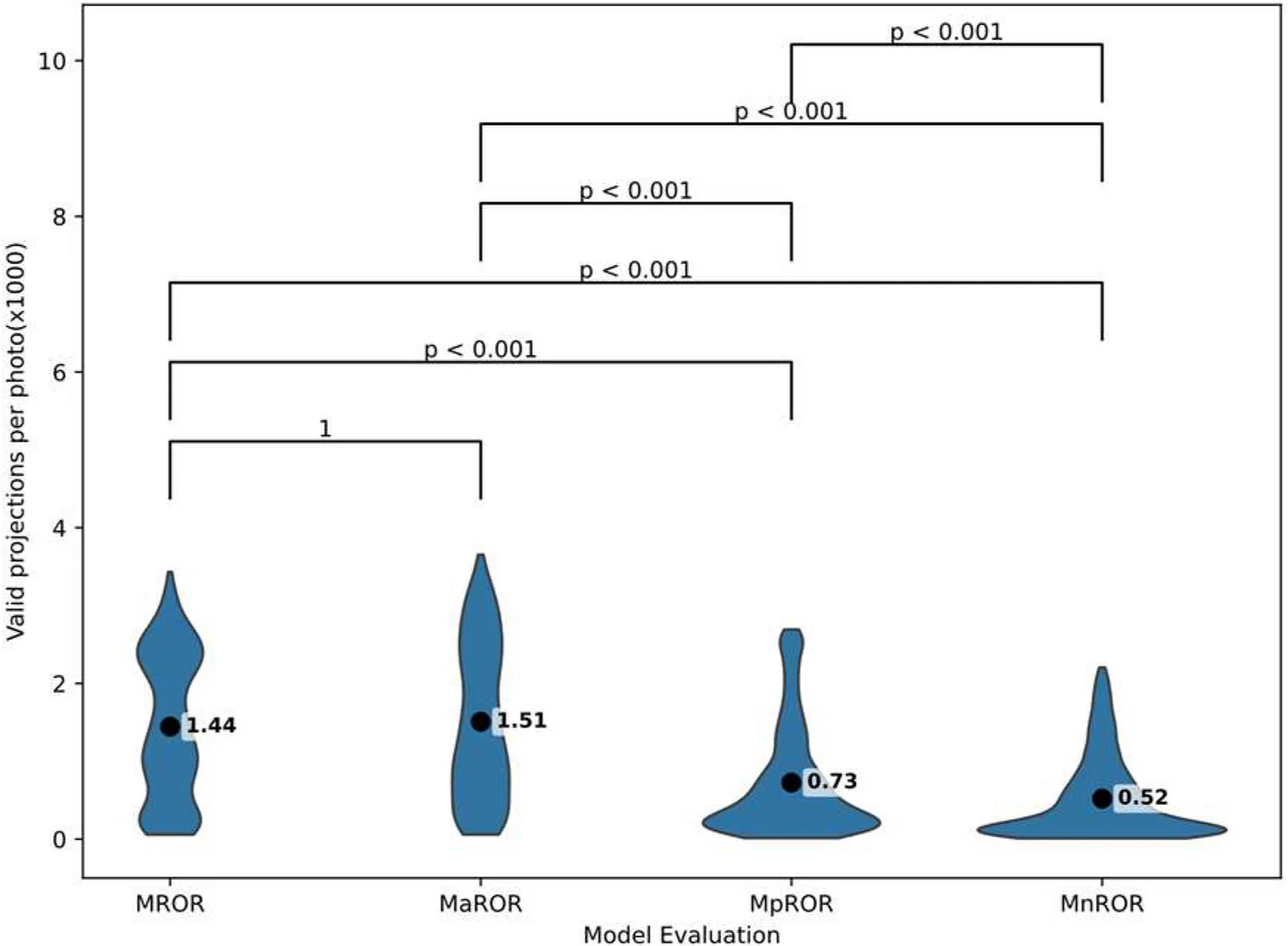
11 Feature valid projections per photo (x1000) with model classification. The number of valid projections per feature was higher in MROR and MaROR (means ≈ 2.3) than in MpROR and MnROR (mean: 2.0; p < 0.001), with no difference between MROR and MaROR

**Fig. 12:**
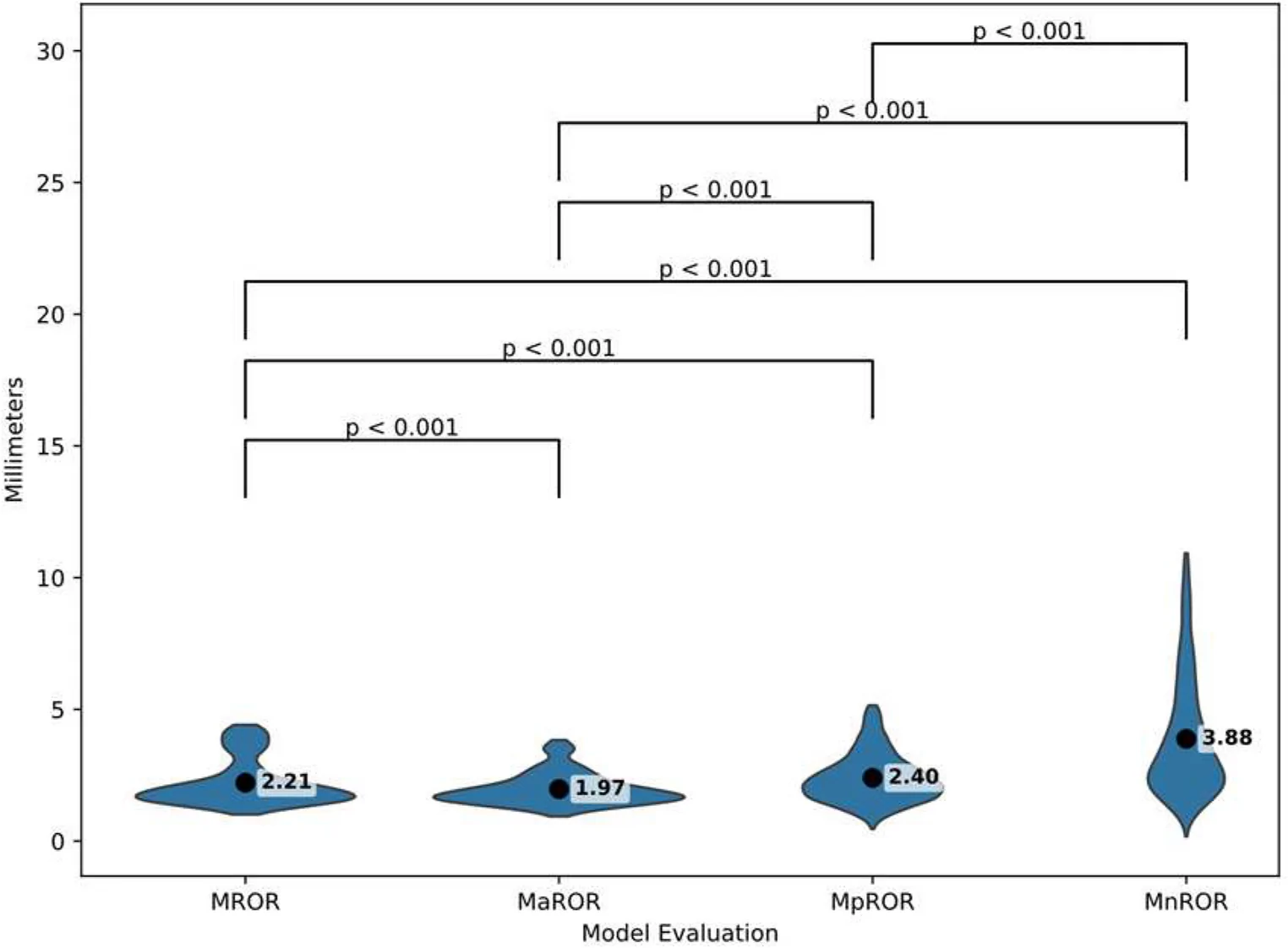
RMS point error per matched feature in millimeters by model classification. RMS point error per matched feature increased from MROR (2.21 mm) and MaROR (1.97 mm) to MpROR (2.40 mm) and MnROR (3.88 mm), with all pairwise differences significant (p < 0.001)

**Fig. 13:**
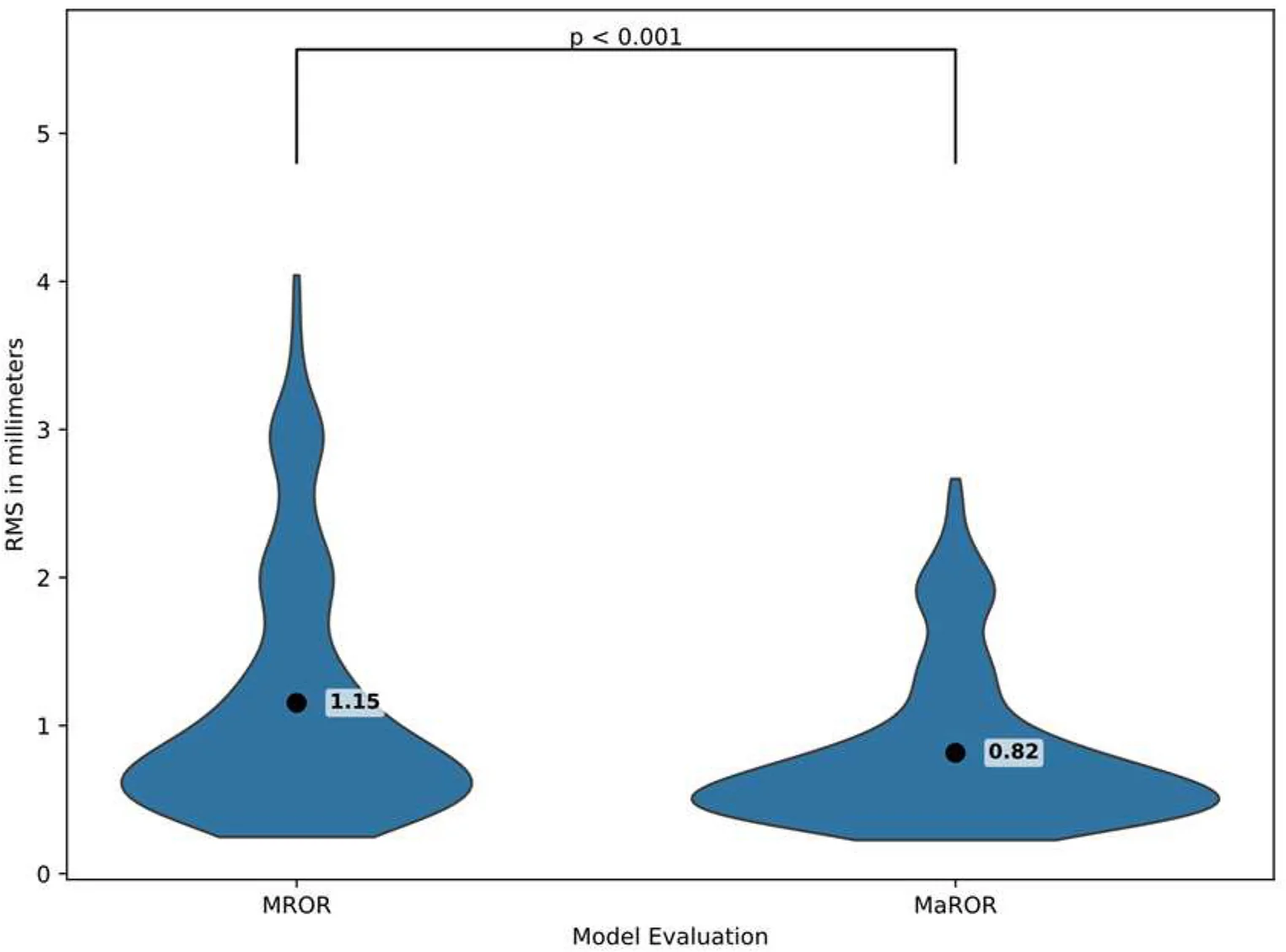
RMS distance from reference model in millimeters by model classification. It was lower in MaROR (mean = 0.82 mm) than in MROR (1.15 mm; p < 0.001)

Quantitative analysis showed clear separation between final model categories, particularly in feature density and reconstruction quality (Fig. 10-13; Supplemental Figures S16–S20). MROR and MaROR behaved similarly across key metrics, with high numbers of matched features (~ 30k–32k) and no significant differences between them, but both were substantially higher than MpROR and MnROR (~ 8–10k) (Fig. 10). 

This pattern was consistent for valid projections per feature, where MROR/MaROR (~ 2.3) exceeded MpROR/MnROR (~ 2.0) (Fig. 11), and for projection density metrics (See Supplemental Material), indicating greater reconstruction support in higher-quality models. Photo alignment ratio was also optimal in MROR and MaROR, while reduced in MpROR and MnROR (See Supplemental Material), further distinguishing successful from suboptimal outcomes.

Error metrics reinforced this separation. RMS point error per matched feature increased progressively from MROR and MaROR (~ 2 mm) to MpROR and MnROR (up to ~ 3.9 mm), with all comparisons significant (*p* < 0.001) (Fig. 12). At the final model level, RMS vertex distance to ground truth was slightly lower in MaROR (~ 0.82 mm) than in MROR (~ 1.15 mm; *p* < 0.001), although both remained within a similar accuracy range (Fig. 13). Together, these findings indicate that MROR and MaROR share comparable quantitative performance, while MpROR and MnROR consistently exhibit degraded reconstruction quality.

Additional metrics shown in Supplemental Material provided complementary trends. MROR and MaROR showed similar processing times, whereas MpROR had longer and MnROR shorter processing time. The valid-to-total projection ratio was highest in MpROR and intermediate in MaROR, but lower in MROR and MnROR, suggesting that this metric alone does not reflect reconstruction quality. Overall, most variables showed statistically significant differences across groups, but the strongest and most consistent distinction was between high-quality (MROR/MaROR) and lower-quality (MpROR/MnROR) models.

### Complete Model vs. Point Cloud and Alignment

The relationship between subjective alignment evaluation and final model quality was statistically significant across all analyses (*p* < 0.001). When MROR and MaROR were treated as separate categories, the correlation was very strong (Cramer’s V = 0.366), driven primarily by positive associations between MROR and Correct Alignment (AC) and between MaROR and AC, as well as inverse associations between MnROR and AC and between MROR and Incorrect Alignment (AI). Grouping MROR and MaROR into a single category further strengthened this relationship (Cramer’s V = 0.448), with the most influential comparisons being [MROR, MaROR]–AC and [MROR, MaROR]–AI, indicating that alignment quality is a strong predictor of final model adequacy.

**Fig. 14:**
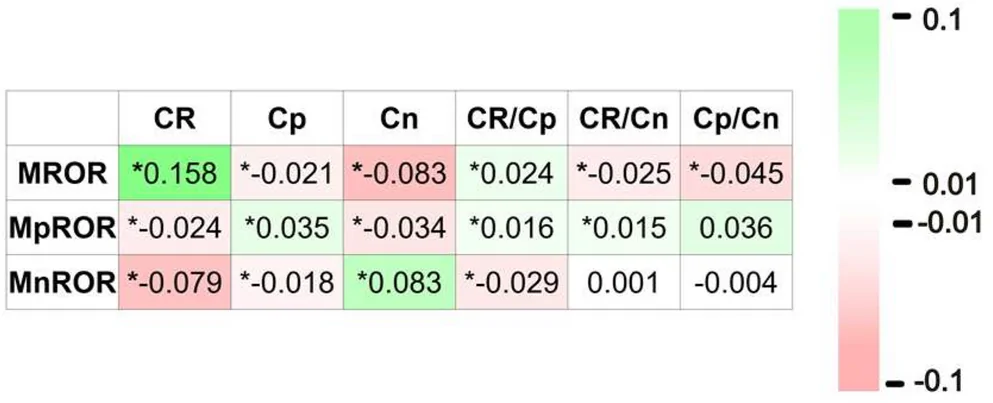
Adjusted standardized residuals of correlations between brain tie point cloud evaluations and complete 3D model evaluations. Asterisks indicate significant values. p < 0.001. Cramer’s V = 0.50

A similarly robust pattern was observed when comparing subjective cloud evaluation with final model quality. Treating MROR and MaROR separately yielded a very strong correlation (Cramer’s V = 0.428), primarily driven by positive associations between CROR and both MROR and MaROR, and negative associations between CROR and MnROR. When MROR and MaROR were combined, the correlation increased further (Cramer’s V = 0.525), with the strongest contributors being [MROR, MaROR]–CROR and MnROR–CnROR (Fig. 14). Because of the slightly higher correlation of cloud evaluation with a complete model evaluation, the former will be used on subsequent analysis.

### Photo Capture Angle

**Fig. 15:**
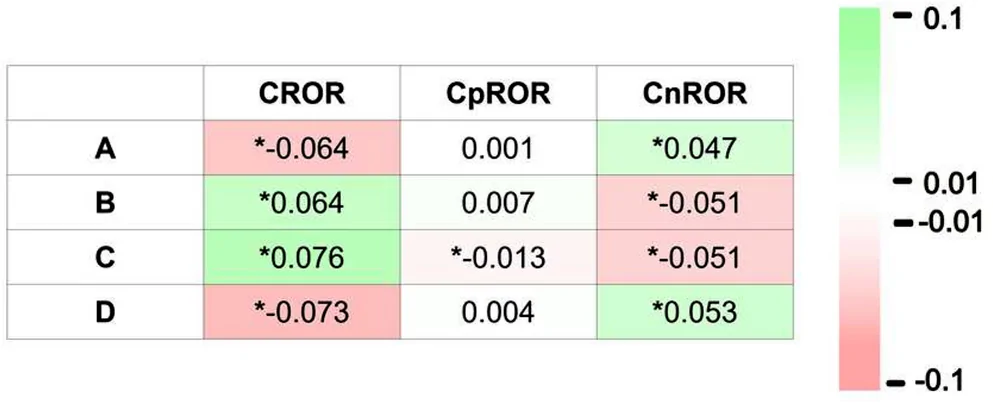
Adjusted standardized residuals of correlations between brain tie point cloud evaluations and photo capture angle configurations. Asterisks indicate significant values. p < 0.001. Cramer’s V = 0.30

When evaluating the correlation between the subjective cloud assessment and the photo capture angle (Fig. 15), the analysis revealed statistical significance (*p* < 0.001) with a very strong correlation (Cramer’s V = 0.30). Groups B and C showed a positive correlation with CROR (0.064 and 0.076, respectively), while groups A and D exhibited a negative correlation (-0.064 and − 0.073). Regarding CpROR, only group C had a value exceeding 0.01 (-0.013). For CnROR, groups A and D showed direct correlations (0.047 and 0.053), whereas groups B and C had inverse correlations (both − 0.051).

Across configurations A–D, MROR and CROR showed highly consistent behavior across all quantitative metrics, with no statistically significant differences either within or between reconstruction types (Supplementary Figures S21–S26). The number of matched features was comparable between MROR and CROR across all configurations, while CpROR and CnROR retained substantially fewer matches (~ 4–20% of MROR/CROR). Similarly, photo alignment ratio remained perfect (1.0) in both MROR and CROR, contrasting with the broader and bimodal distributions observed in CpROR and CnROR, indicating reduced alignment stability in lower-quality models, (See Supplementary Material for Figures).

**Fig. 16:**
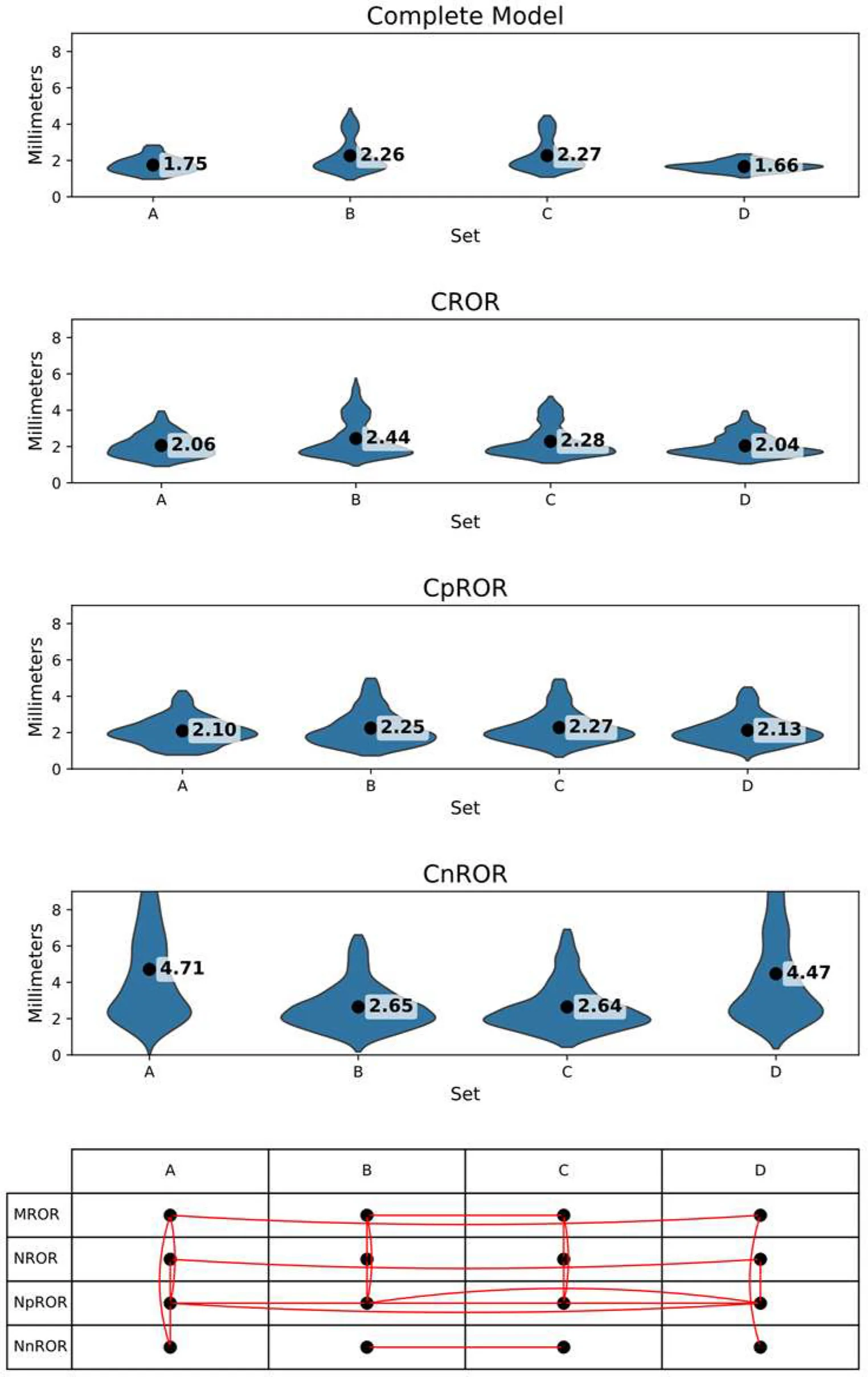
RMS point error per matched feature in millimeters by capture angle. MROR showed values ranging from 1.66 ± 0.26 (d) to 2.59 ± 1.13 (c), while CROR ranged from 2.04 ± 0.60 (d) to 2.44 ± 1.03 (b). Red lines in the bottom matrix shows no significant correlations (p > 0.05)

**Fig. 17:**
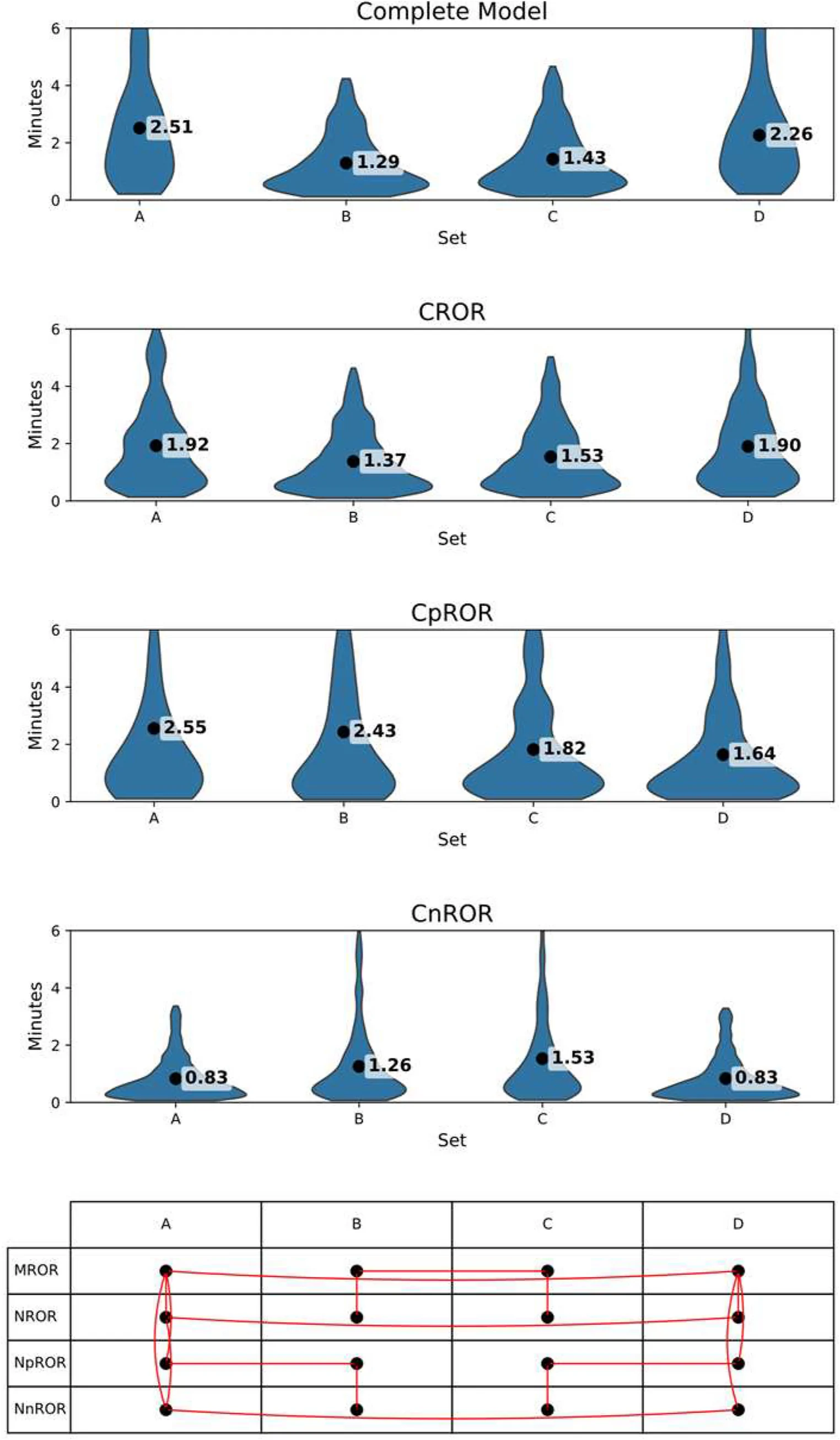
SfM processing time in minutes by capture angle. Time varied across set configurations but was comparable between MROR (1.29–2.51 min) and CROR (1.37–1.92 min). Most within-model differences were significant (p < 0.001), except (**a**) vs (**d**) and (**b**) vs (**c**); no differences were observed between MROR and CROR (p ≥ 0.16). Red lines in the bottom matrix shows no significant correlations (p > 0.05)

Error and performance metrics further supported the equivalence between MROR and CROR. RMS point error per matched feature showed similar ranges in both groups (~ 1.7–2.6 mm), with no consistent differences between reconstruction types (Fig. 16), although CnROR tended to exhibit higher errors, particularly in some configurations. SfM processing time followed comparable patterns in MROR (~ 1.3–2.5 min) and CROR (~ 1.4–1.9 min), with configuration-dependent differences but no differences between reconstruction strategies (Fig. 17).

**Fig. 18:**
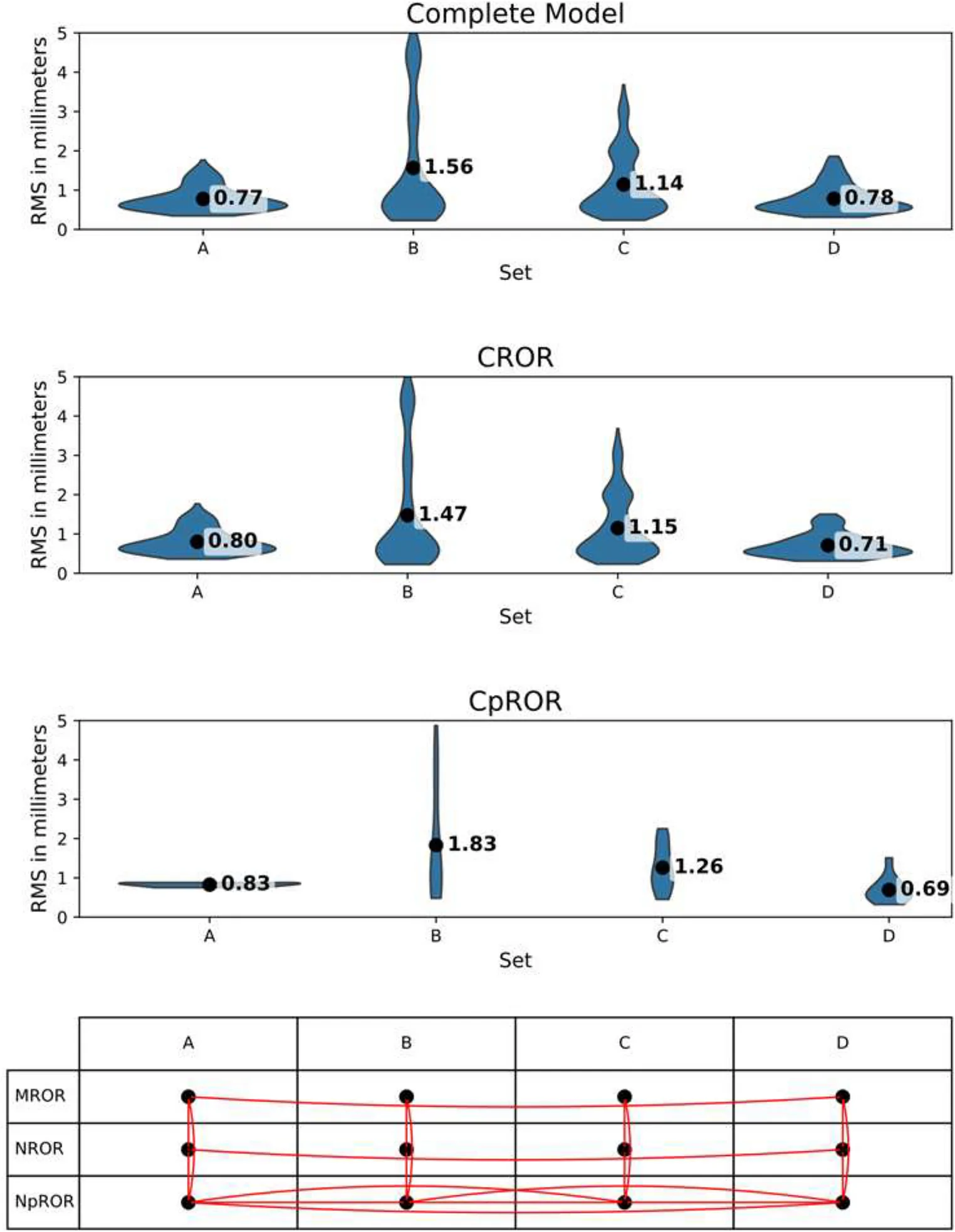
RMS distance from reference model in millimeters by capture angle. It was similar between MROR (0.77–1.56) and CROR (0.71–1.47), with no differences between groups (p = 1), indicating comparable geometric accuracy. Red lines in the bottom matrix shows no significant correlations (p > 0.05)

Geometric accuracy was also equivalent between MROR and CROR. RMS vertex distance to the reference model showed overlapping ranges (~ 0.7–1.6 mm) and no significant differences across configurations (Fig. 18). Secondary metrics, including valid projections per feature, projections per photo, and projection ratios, were likewise similar between MROR and CROR, while CpROR and CnROR consistently showed reduced projection support (See Supplementary Material).

### Number of Photos

**Fig. 19:**
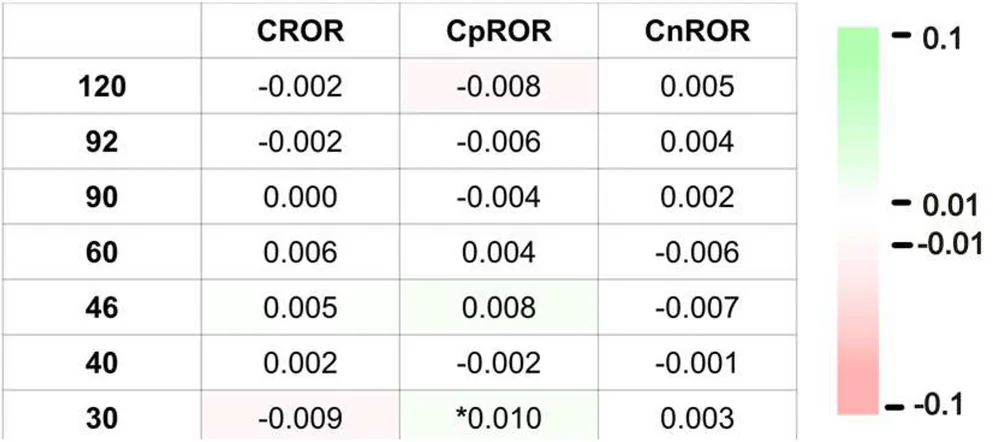
Adjusted standardized residuals of correlations between brain tie point cloud evaluations and photo number configurations. Asterisks indicate significant values. p < 0.001. Cramer’s V = 0.30

When analyzing the correlation between the subjective evaluation of dense clouds and the number of photos per set, the result was statistically significant (*p* < 0.001). However, the correlation strength was negligible to non-existent, as indicated by a very low Cramer’s V value of 0.02. Among the comparisons, only the correlation between CpROR and the 30-photo set exceeded our defined threshold, with a value of 0.010 (Fig. 19).

The number of matched features increased consistently with photo count in both MROR and CROR (Supplementary Figure S27), rising from ~ 12k at 30 photos to ~ 50–58k at 120 photos. Most pairwise comparisons were statistically significant, except between adjacent configurations (40–46 and 90–92 photos), and no differences were observed between MROR and CROR at any photo count. Despite this increase in feature density, photo alignment remained perfect (ratio = 1.0) across all configurations (Supplementary Figure S28), and projection-based metrics showed only modest changes, with higher-density sets (≥ 90 photos) yielding slight increases in projections but reduced projection ratios (Supplementary Figures S31, S33).

Importantly, these large differences in matched feature counts did not translate into improved geometric accuracy. RMS vertex error relative to ground truth remained stable across all photo counts in both MROR and CROR, with overlapping ranges (~ 0.8–1.2 mm) and no statistically significant differences (Supplementary Figure S29). This indicates that increasing photo count enhances feature matching density but does not meaningfully improve the accuracy of the final reconstructed surface.

**Fig. 20:**
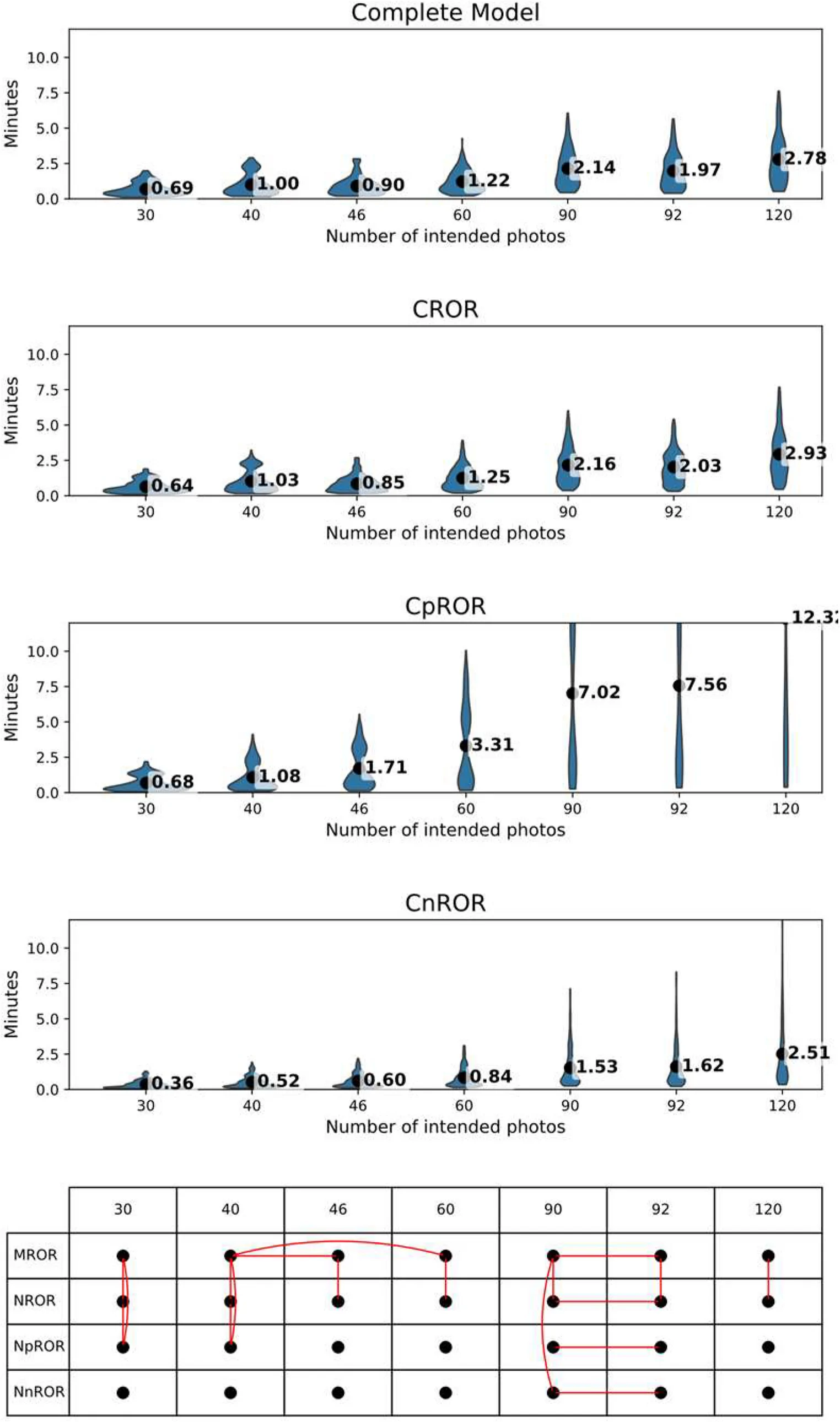
SfM processing time in minutes by number of photos. Mean processing time increased from ~ 0.6–0.7 min (30 photos) to ~ 2.8–2.9 min (120 photos), with most differences significant (p < 0.001) except between adjacent groups (e.g., 40–46, 90–92). No differences were observed between MROR and CROR. Red lines in the bottom matrix shows no significant correlations (p > 0.05)

In contrast, computational costs increased substantially with photo count (Fig. 20). SfM processing time rose from ~0.6–0.7 minutes at 30 photos to ~2.8–2.9 minutes at 120 photos, with most differences between groups being statistically significant. As with other metrics, no differences were observed between MROR and CROR, confirming equivalent computational behavior 

### Photo’s Density

**Fig. 21:**
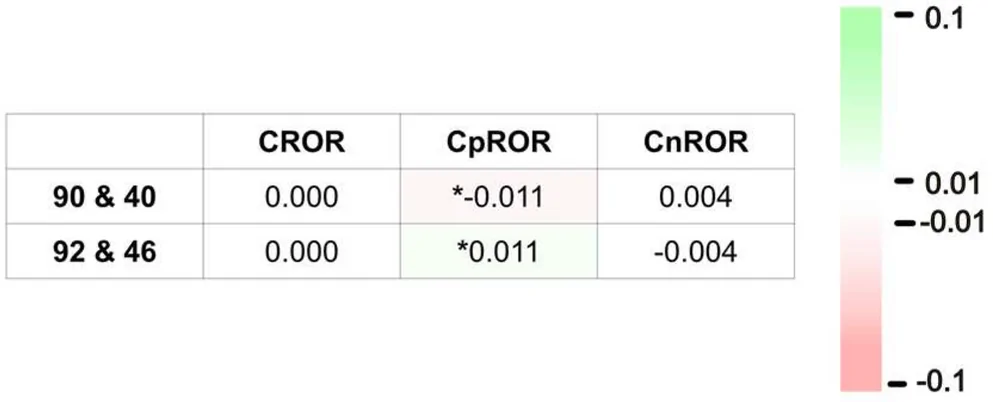
Adjusted standardized residuals of correlations between brain tie point cloud evaluations and photo density configurations. Asterisks indicate significant values. p < 0.001. Cramer’s V = 0.06

Examining the correlation between the subjective evaluation of point clouds and the density of photos (continuous or variable), the results were statistically significant (*p* < 0.001) but showed a weak correlation (Cramer’s V = 0.06). Having a variable density of photos (92 & 46 photos) weakly correlates to CpROR (0.011) (Fig. 21).

Comparisons between adjacent photo-count configurations showed minimal differences. Specifically, no statistically significant differences were observed between the 40- and 46-photo sets or between the 90- and 92-photo sets across the main quantitative metrics, including number of matched features, photo alignment ratio, median valid and total projections per photo, projection ratios, SfM processing time, and RMS vertex error relative to ground truth (*p* ≥ 0.05). Both complete models (MROR) and high-quality dense clouds (CROR) exhibited equivalent behavior in these comparisons.

### Number of Masks Applied

**Fig. 22:**
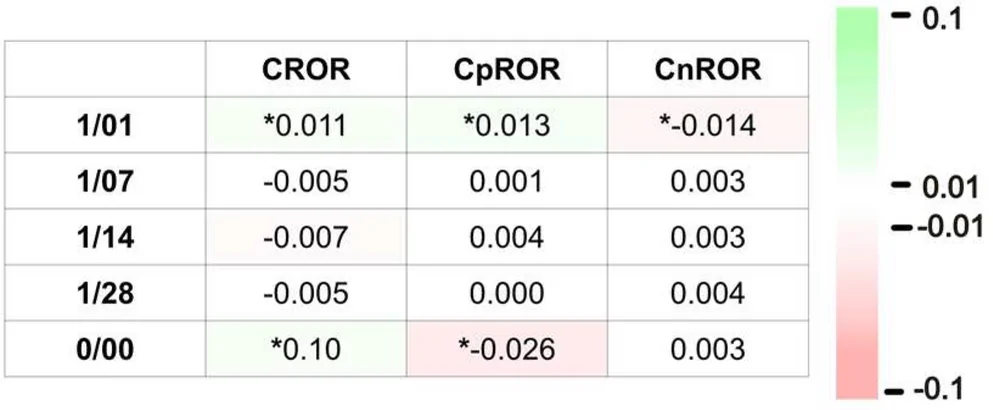
Adjusted standardized residuals of correlations between brain tie point cloud evaluations and photo density configurations. Asterisks indicate significant values. *p* < 0.001. Cramer’s V = 0.06

Exploring the relationship between the subjective evaluation of point clouds and the number of masks applied per image (Fig. 22), the results were statistically significant (*p* < 0.001) but revealed a weak correlation (Cramer’s V = 0.07). For CROR, the most significant correlation was observed when applying masks to all images (0.011) and when no masks were applied to any images (0.010). Regarding CpROR, applying masks to each image showed a positive correlation (0.013), while not applying any masks demonstrated a negative correlation (-0.026). For CnROR, applying masks to every image exhibited **Fig. **22 Adjusted standardized residuals of correlations between brain tie point cloud evaluations and masks applied per image configurations. Asterisks indicate significant values. *p* < 0.001. Cramer’s V = 0.07.

a negative correlation (-0.014).

**Fig. 23:**
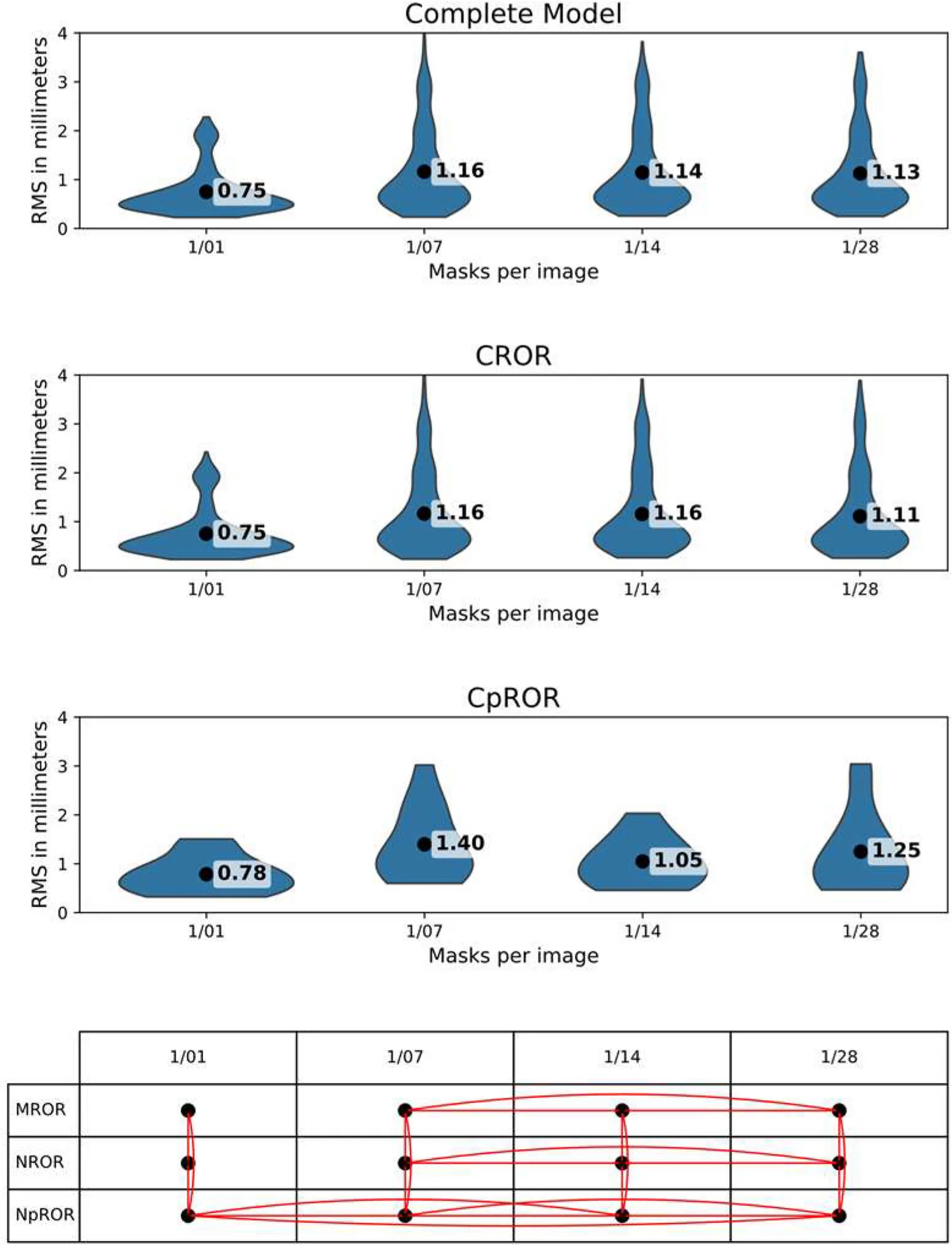
RMS distance from reference model in millimeters by number of masks applied. The 1/01 configuration yielded lower RMS values (~ 0.4 mm; ~35% reduction) than 1/07, 1/14, and 1/28 (*p* < 0.001), with no differences among the latter or between MROR and CROR. Red lines in the bottom matrix shows no significant correlations (*p* > 0.05)

Masking strategy influenced several intermediate metrics, although most effects were modest and consistent between MROR and CROR (Supplemental Figure S35–S42). Applying masks to every image (1/01) produced a small but consistent increase in matched features (~ 16%) compared with lower masking frequencies (1/07, 1/14, 1/28), which behaved similarly. RMS point error per matched feature also followed this pattern, with 1/01 yielding slightly lower values, while the remaining configurations showed no differences among them (See Supplemental Material). Projection-based metrics showed limited sensitivity to masking density: 1/01 resulted in moderately higher valid projections per photo, whereas total projections and projection ratios remained largely unchanged across configurations (See Supplemental Material). Photo alignment ratio was invariant (1.0) in all cases, and no differences were observed between MROR and CROR.

Processing time was largely unaffected by masking in MROR and only modestly reduced in CROR for the 1/01 configuration (~ 12% decrease) (See Supplemental Material). In contrast, the most relevant effect of masking was observed in final geometric accuracy (Fig. 23). The 1/01 configuration consistently produced lower RMS vertex distances to the reference model in both MROR and CROR, with an average reduction of ~ 0.4 mm (~ 35%) compared with the other masking strategies (*p* < 0.001). No differences were observed among 1/07, 1/14, and 1/28, and MROR and CROR behaved equivalently across all comparisons.

### Masks Configurations

**Fig. 24:**
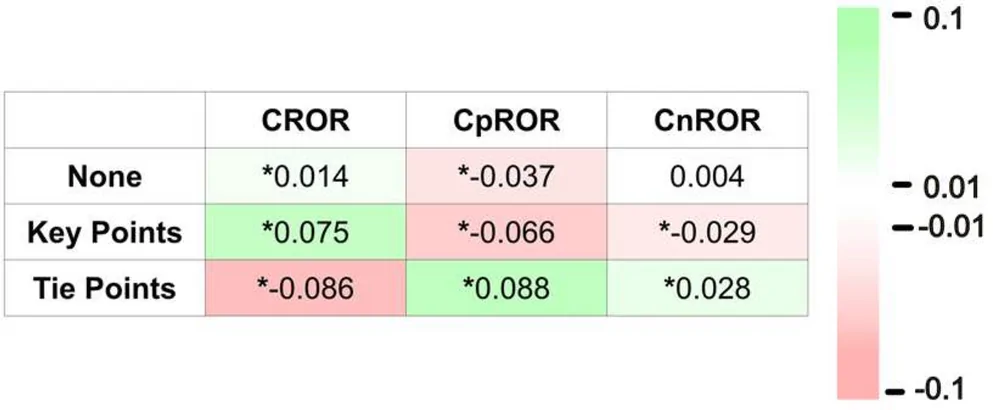
Adjusted standardized residuals of correlations between brain tie point cloud evaluations and masks application configurations. Asterisks indicate significant values. p < 0.001. Cramer’s V = 0.24

When comparing the correlation between subjective cloud evaluations and the mask application configuration (Fig. 24), the results were statistically significant (*p* < 0.001) with a strong correlation (Cramer’s V = 0.24). The most significant correlation with CROR was observed when configuring the mask application as tie points (-0.086), followed by key points (0.075), and finally not applying any mask (0.014). For CpROR, there was a positive correlation with tie points (0.088) and negative correlations with key points (-0.06) and no mask (-0.03). Similarly, for CnROR, a positive correlation was found with tie points (0.028) and a negative correlation with key points (-0.029).

Across masking strategies, MROR and CROR exhibited highly comparable behavior in all quantitative metrics, with no statistically significant differences between reconstruction types (Supplemental Figures S43–S50). Under no masking (N) and detected feature masking (K), both groups showed similarly high numbers of matched features (~ 37k–40k), while matched feature masking (T) produced a marked reduction to ~ 30% of these values (Supplemental Figure S43). A consistent decline under T was also observed in projection-based metrics: valid projections per photo and total projections per photo dropped to ~ 35–45% of N and K values (Supplemental Figure S47–S48). In contrast, projection ratios increased under T (to ~ 0.78–0.79), indicating a higher proportion of valid matches despite fewer total projections (Supplemental Figure S49). Across all configurations, alignment ratio remained perfect (mean = 1.0) for MROR and CROR (Supplemental Figure S44), and RMS point error per matched feature showed no meaningful differences between them (Supplemental Figure S45).

**Fig. 25:**
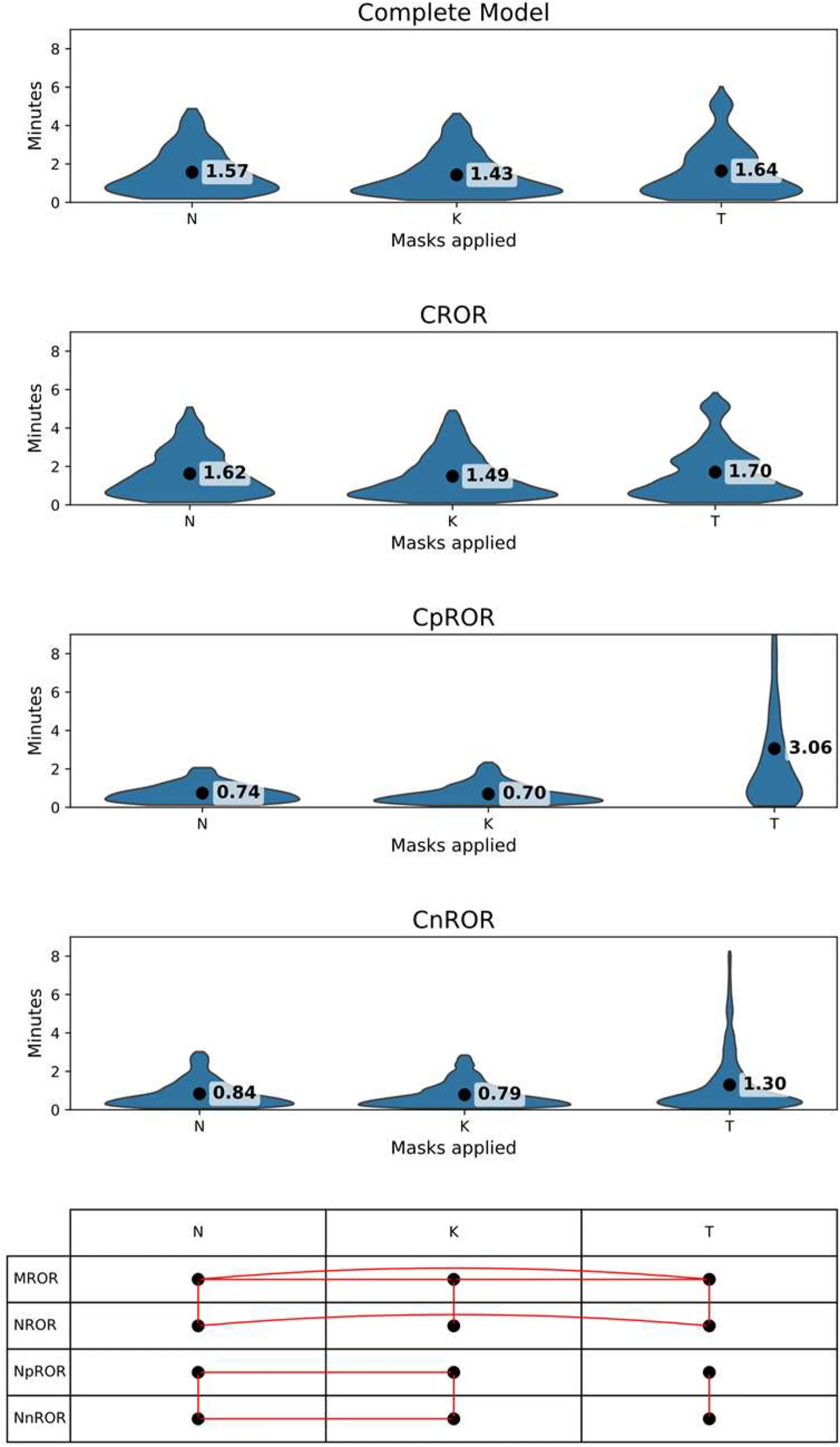
SfM processing time in minutes by mask configuration. Time was similar between MROR (1.43–1.64 min) and CROR (1.49–1.70 min) across masking strategies. In CROR, key-point masking was ~ 23% faster than tie-point masking (p < 0.001), while no differences were observed in MROR. CpROR and CnROR showed reduced times overall, except for increased time with tie-point masking in CpROR. Red lines in the bottom matrix shows no significant correlations (p > 0.05)

Processing time shows comparable computational performance between MROR and CROR across masking strategies (Fig. 25). Mean times ranged from ~ 1.4 to 1.7 min, with no significant differences within MROR, and only a modest but significant reduction for key-point masking compared to tie-point masking in CROR (~ 23% faster). Importantly, despite substantial differences in feature density and projection counts, RMS vertex distance relative to ground truth remained stable across all masking strategies and reconstruction types (Supplemental Figure S50), indicating that geometric accuracy of the final surface is largely insensitive to masking configuration.

### Post Hoc Masks Number on Masks Applied to Key Points

**Fig. 26:**
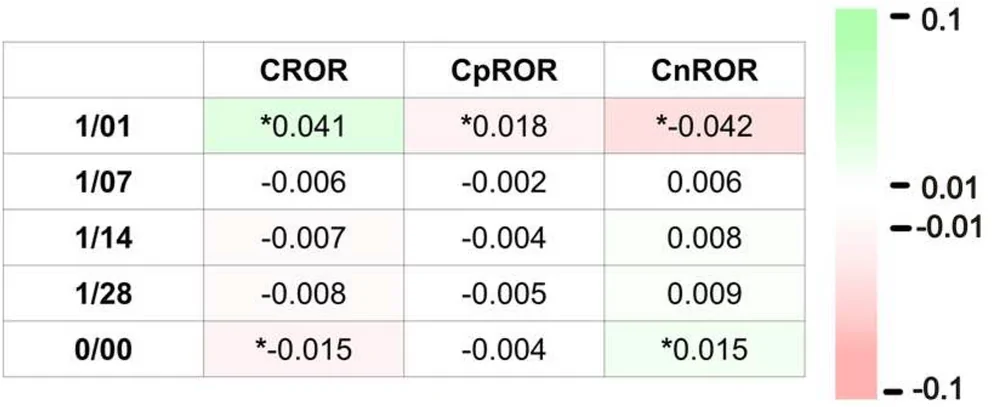
Adjusted standardized residuals of correlations between brain tie point cloud evaluations and masks application configurations within the subgroup where masks were applied to key points. Asterisks indicate significant values. p < 0.001. Cramer’s V = 0.13

When analyzing the correlation between the subjective evaluation of point clouds and the number of masks per image within the subgroup where masks were applied to key points (Fig. 26), the results were statistically significant (*p* < 0.001), showing a moderate correlation (Cramér’s V = 0.13). In the CROR group, the most significant correlation was observed when masks were applied to all images, showing a positive correlation (0.041), while not applying any masks showed a negative correlation (-0.015). This trend was reversed in the CnROR group, where applying masks to all images showed a negative correlation (-0.042), and not applying any masks showed a positive correlation (0.015). It is worth noting that, for both CROR and CnROR, intermediate configurations of mask application per image (1/07, 1/14, 1/28) did not surpass the standardized residual threshold of 0.01.

### Alignment Precision

**Fig. 27:**
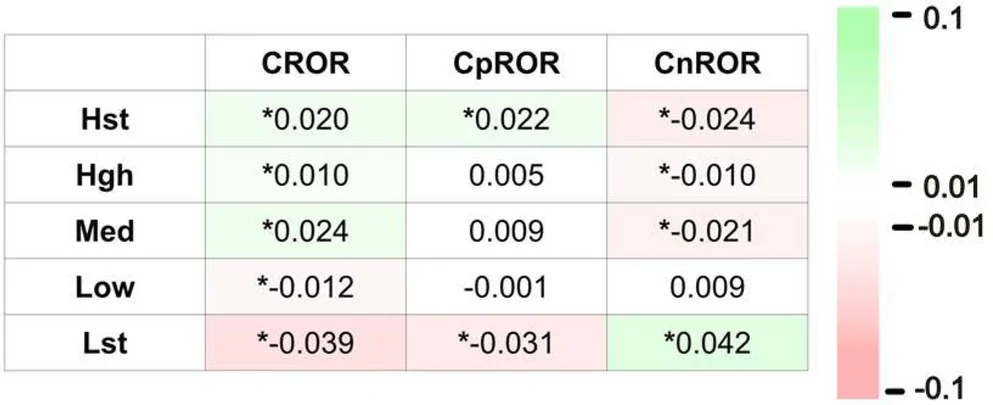
Adjusted standardized residuals of correlations between brain tie point cloud evaluations and alignment precision configurations. Asterisks indicate significant values. p < 0.001. Cramer’s V = 0.17

When analyzing the correlation between the subjective evaluation of point clouds and alignment precision (Fig. 27), the results were statistically significant (*p* < 0.001), demonstrating a strong correlation (Cramér’s V = 0.17). In the CROR group, the most significant correlation was negative, associated with the lowest precision (-0.039), followed by a positive correlation with medium precision (0.024) and high precision (0.020). The CpROR group showed a positive correlation with the highest precision (0.022) and a negative correlation with the lowest precision (-0.031). Conversely, the CnROR group exhibited a positive correlation with the lowest precision (0.024) and negative correlations with medium precision (-0.021) and the highest precision (-0.024).

**Fig. 28:**
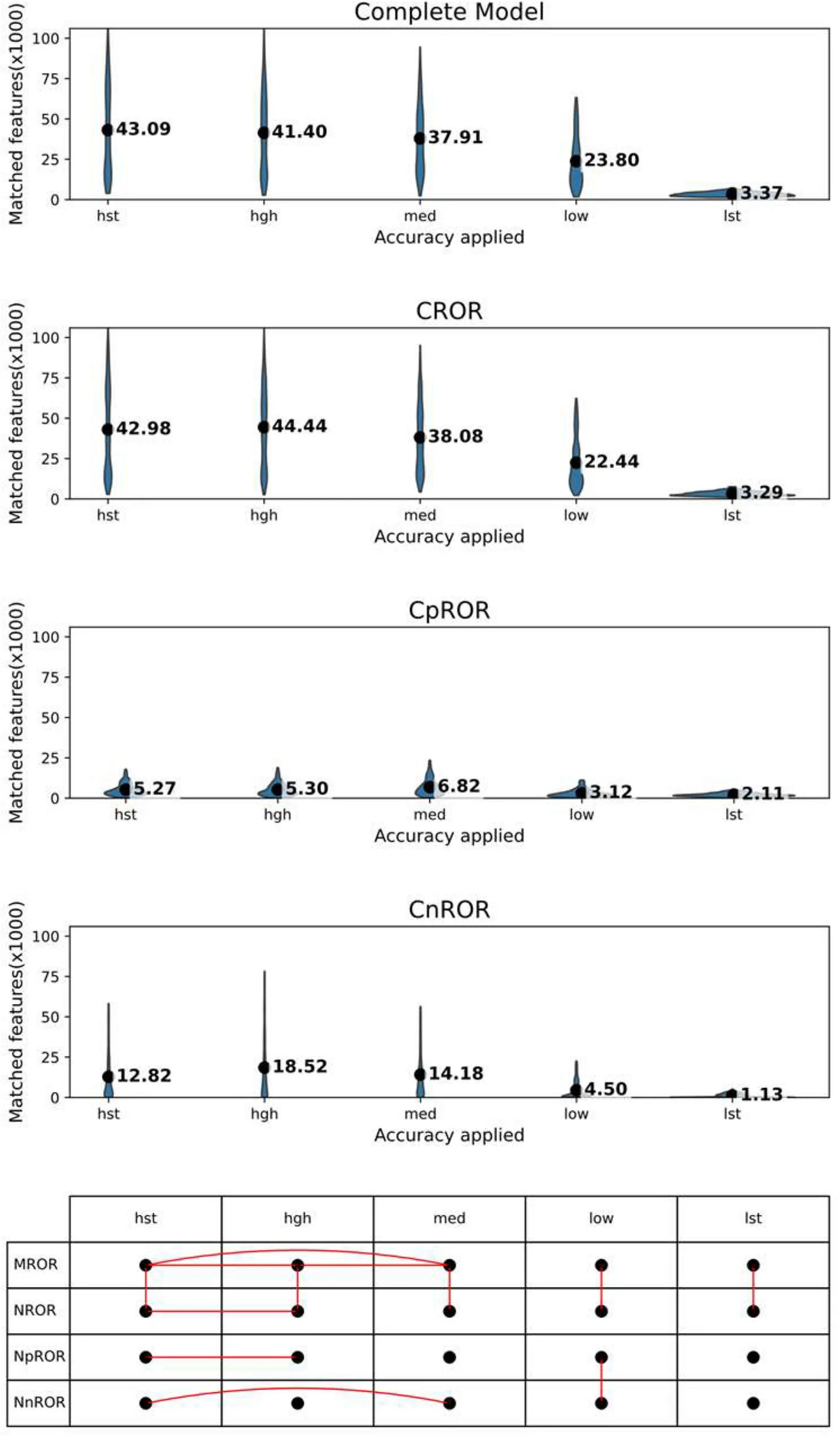
Matched features (x1000) by alignment precision. They were highest at highest–high–medium accuracy in MROR (~43k–38k; no differences, p = 1), decreased by ~50% at low (~23.8k), and dropped to ~3.4k (~8%) at lowest (p < 0.001). CROR showed the same trend. CpROR (12–62%) and CnROR (30–35%) consistently yielded fewer matched features than MROR/CROR. Red lines in the bottom matrix shows no significant correlations (p > 0.05)

**Fig. 29:**
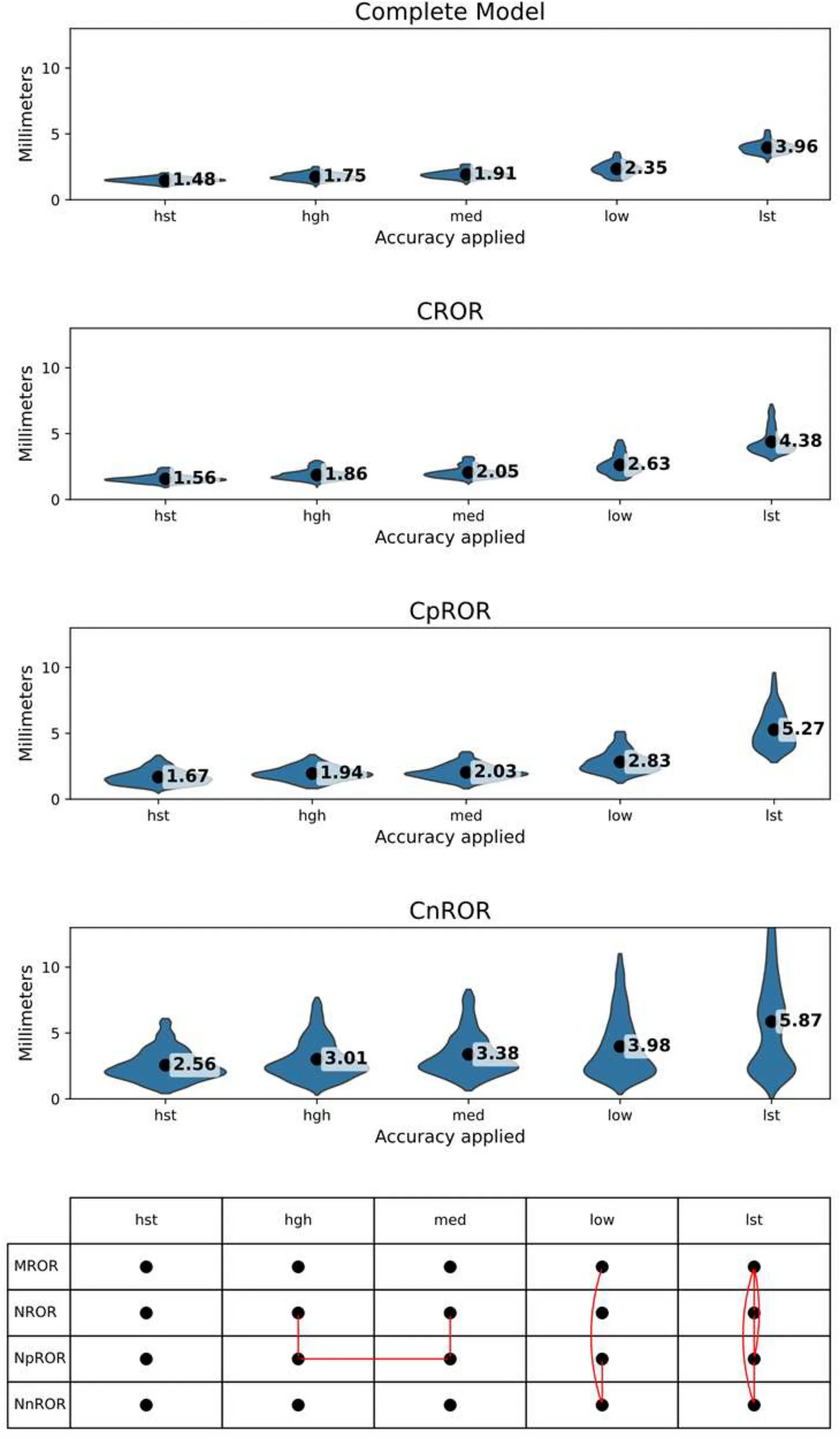
RMS point error per matched feature in millimeters by alignment precision. RMS point error increased with decreasing accuracy in MROR (1.48 ± 0.20 at highest to 3.96 ± 0.42 at lowest; all p < 0.001). CROR showed a similar trend, with slightly higher values (~5–12%) but no difference at lowest (p = 1). This pattern was consistent in CpROR and CnROR. Red lines in the bottom matrix shows no significant correlations (p > 0.05)

**Fig. 30:**
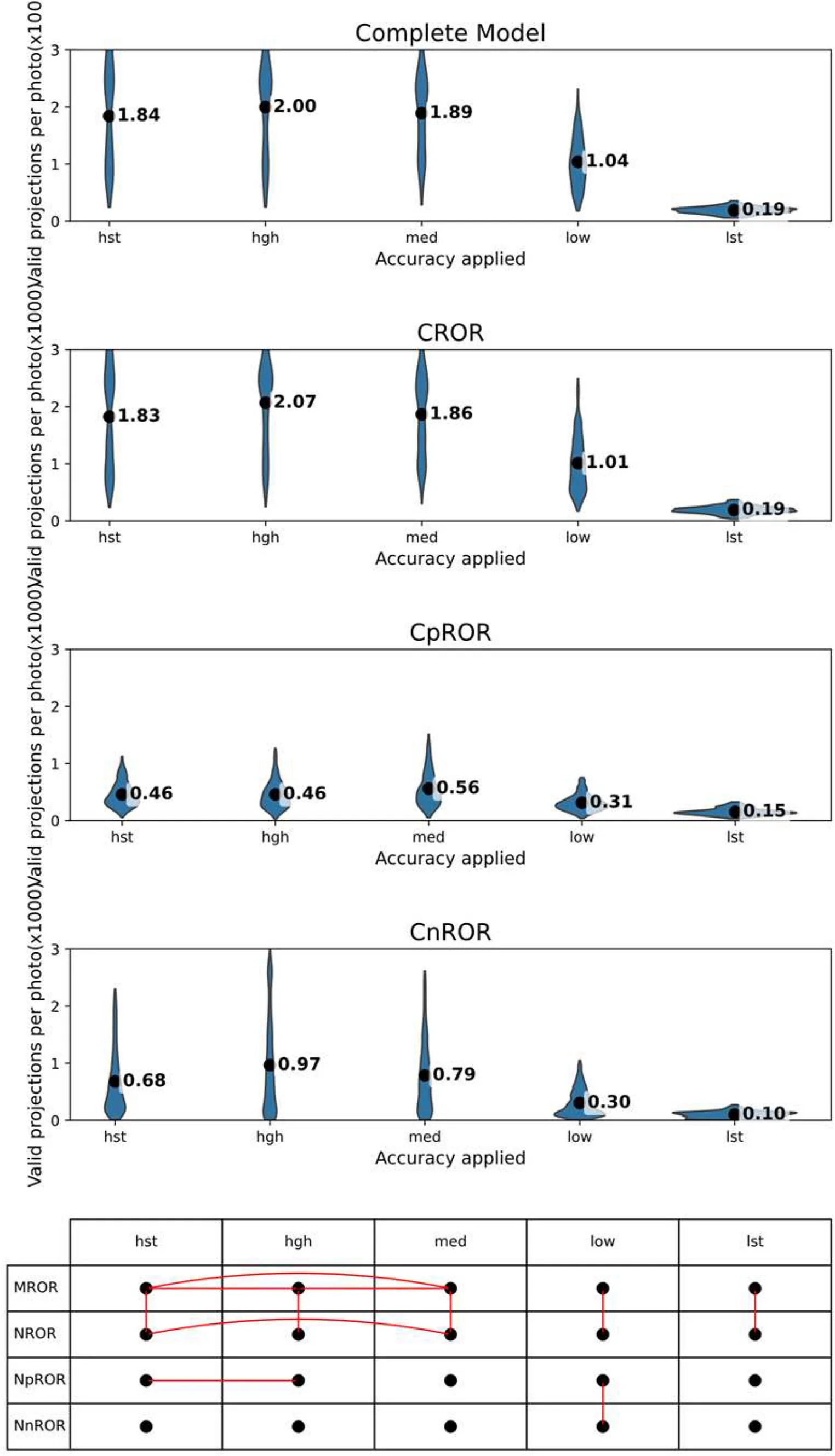
Valid projection per photo (x1000) by alignment precision. It decreased with lower accuracy. In MROR, highest–high–medium formed a stable group (medians 2,039–2,269), dropping ~50% at low (1,005) and to ~20% at lowest (192). CROR showed the same pattern. Most comparisons were significant (p < 0.001) except within the high–medium group, with no differences between MROR and CROR. CpROR (20–80%) and CnROR (37–50%) showed reduced values. Total projections followed a similar trend. Red lines in the bottom matrix shows no significant correlations (p > 0.05)

**Fig. 31:**
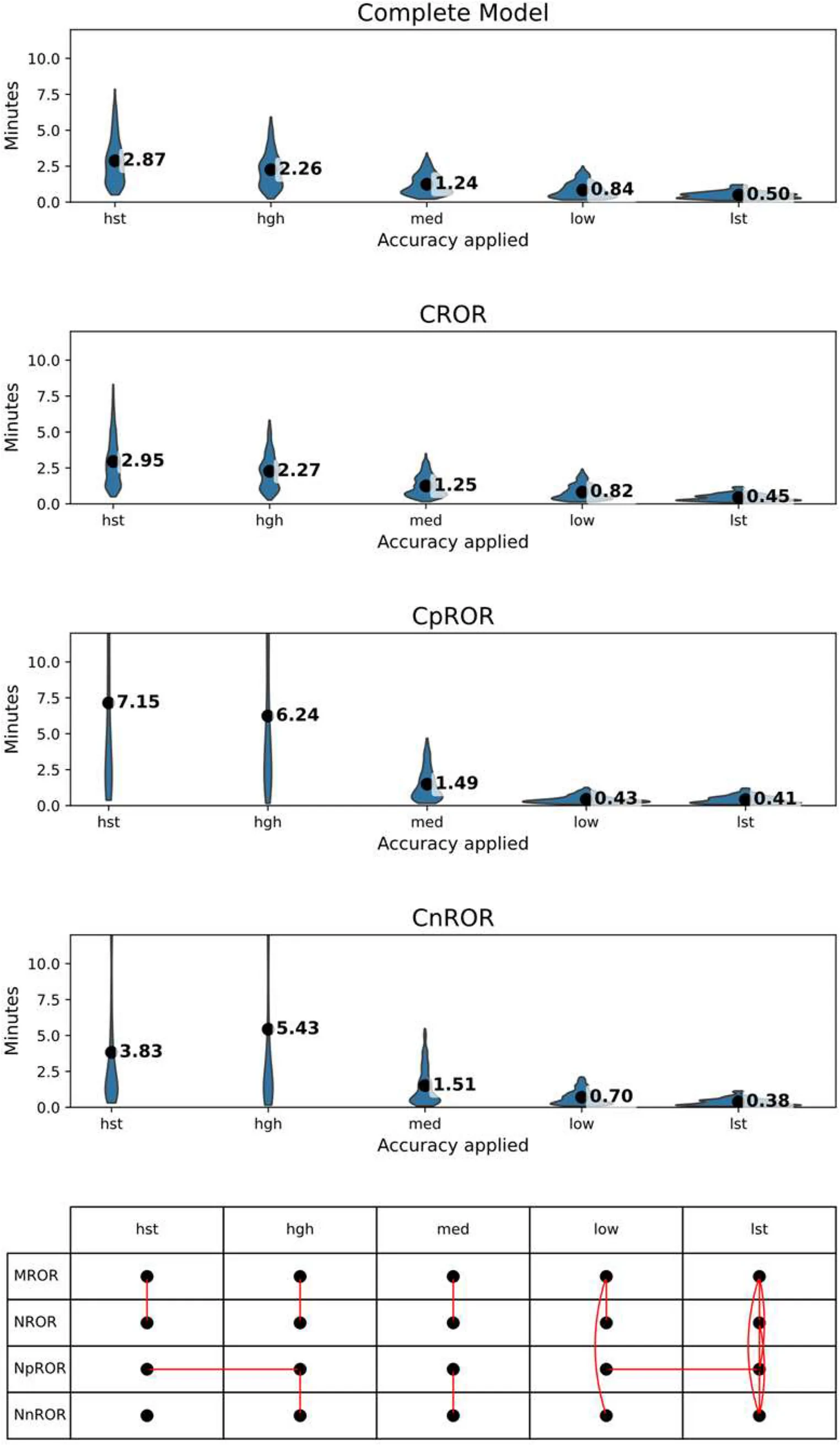
SfM processing time in minutes by alignment precision. Time decreased with lower accuracy in both MROR (2.87 ± 1.59 to 0.50 ± 0.25 min) and CROR (2.95 ± 1.67 to 0.45 ± 0.24 min), representing a ~ 75–80% reduction (all *p* < 0.001). No differences were observed between MROR and CROR. CpROR and CnROR showed higher variability, with highest/high settings up to 2–3× slower (max ~ 12 min). Red lines in the bottom matrix shows no significant correlations (*p* > 0.05)

**Fig. 32:**
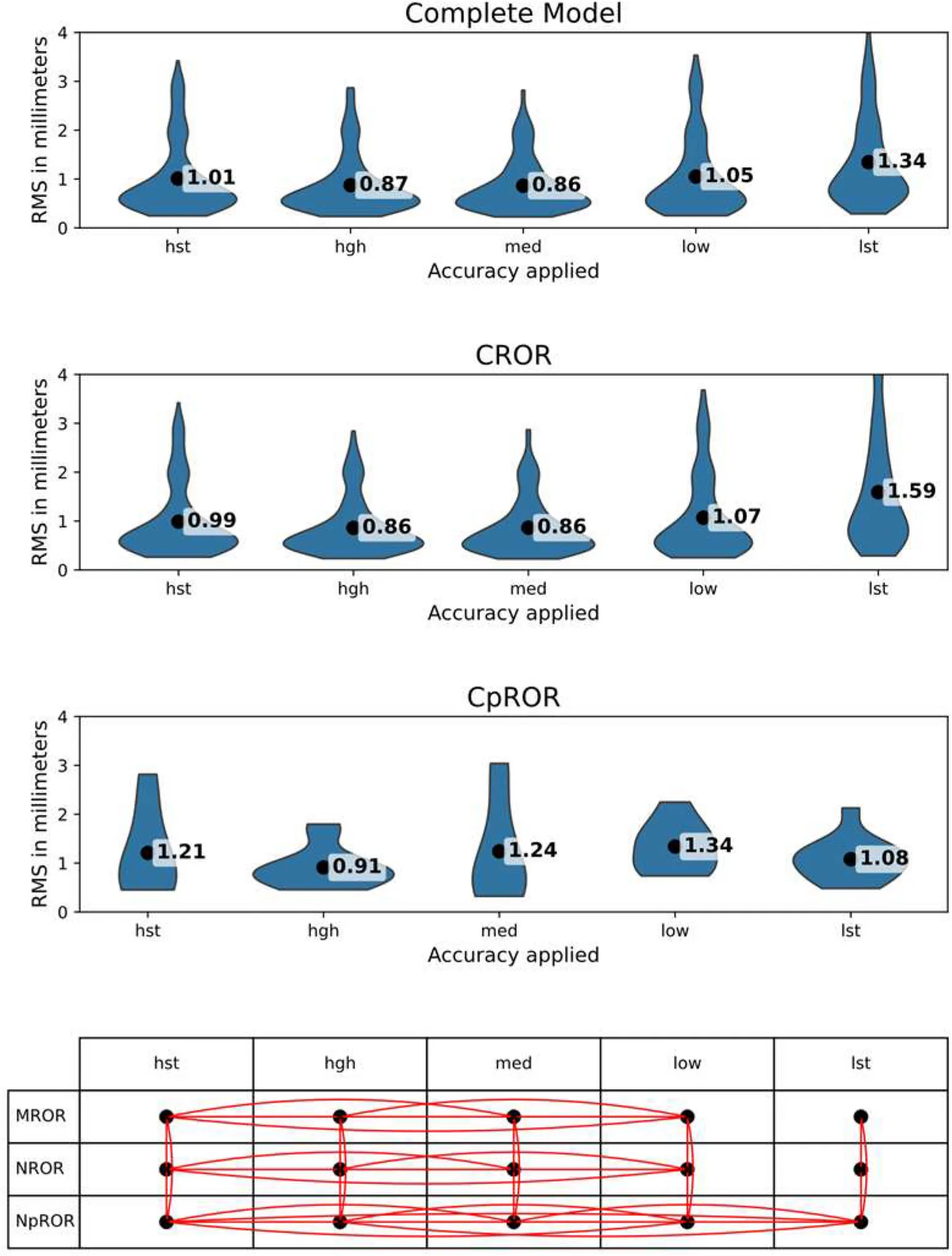
millimeters by alignment precision. It was largely insensitive to accuracy and reconstruction type. MROR ranged from 0.86–1.01 mm (highest–medium) to 1.34 mm (lowest), with CROR showing similar values (0.86–0.99 mm; 1.59 mm at lowest). Differences were minimal, with no MROR–CROR differences (p = 1), except for a consistent increase at lowest accuracy (p < 0.001). Red lines in the bottom matrix shows no significant correlations (p > 0.05)

Across accuracy settings, MROR and CROR exhibited highly consistent and comparable behavior across all quantitative metrics, with no statistically significant differences between reconstruction types (Fig. 28–32; SI, Figs. 51, 52, 53 and 54). The number of matched features decreased progressively as accuracy was reduced (Fig. 30), with highest, high, and medium settings forming a stable group, while low reduced feature counts by approximately half and lowest reduced them to ~ 8% of high–medium values. A similar monotonic trend was observed for RMS point error per matched feature, which increased as accuracy decreased (Fig. 29). Projection-based metrics followed the same pattern: the median number of valid projections per photo remained stable at higher accuracies but dropped sharply at low (~ 50% reduction) and lowest (~ 20% of low) settings (Fig. 30). In contrast, projection ratios remained relatively stable across settings, with only a noticeable decrease at the lowest accuracy (Supplementary Figure S54). Across all these variables, MROR and CROR showed nearly identical distributions.

Processing time was strongly influenced by accuracy (Fig. 31), decreasing by ~ 75–80% from highest to lowest settings, with all comparisons statistically significant within groups but no differences between MROR and CROR. Despite these large differences in feature density and processing cost, geometric accuracy remained largely stable: RMS vertex distance relative to reference model showed minimal variation across accuracy levels and no differences between reconstruction types, except for a slight degradation at the lowest setting (Fig.32). Supporting metrics, including alignment ratio and valid projections per feature, remained largely invariant or showed only minor changes across

### Alignment Variability

When analyzing variability of subjective cloud evaluation across 12,600 sets (Fig. 32), 4,116 sets (32.66%) showed no variability among the five rendered models (SD = 0). A further 4,283 sets (33.99%) exhibited low variability (0 < SD < 0.6), likely attributable to inter-user rating differences, while 4,201 sets (33.35%) demonstrated high variability (SD > 1).

Variability across repeated renderings showed distinct patterns depending on the metric analyzed (Fig. 34; Supplemental Figures S55–S61). The RMS of point error per matched feature (Fig. 34) revealed that variability was generally low at central tendencies but increased substantially at higher percentiles, rising from minimal dispersion at the median to markedly higher values in the upper tail. In contrast, some structural metrics showed relative higher stability: the number of valid projections per matched feature (Supplemental Figure S57), and photo alignment ratio remained highly consistent, with variability emerging only at extreme percentiles **Fig. **30 Valid projection per photo (x1000) by alignment precision. It decreased with lower accuracy. In MROR, highest–high–medium formed a stable group (medians 2,039–2,269), dropping ~ 50% at low (1,005) and to ~ 20% at lowest (192). CROR showed the same pattern. Most comparisons were significant (p < 0.001) except within the high–medium group, with no differences between MROR and CROR. CpROR (20–80%) and CnROR (37–50%) showed reduced values. Total projections followed a similar trend. Red lines in the bottom matrix shows no significant correlations (p > 0.05).

Other projection-based metrics showed moderate but increasing variability. Both valid and total projections per photo displayed low variability in a substantial proportion of groups (≈ 30–47% with SD = 0), but dispersion increased sharply toward higher percentiles (Supplemental Figures S58–S59). A similar trend was observed for projection ratios, which remained stable in most cases but exhibited increasing spread at higher percentiles (Supplemental Figure S60). The number of matched features showed one of the widest variability ranges, with most groups exhibiting some dispersion and extreme variability at upper percentiles (Supplemental Figure S55).

Processing time behaved differently from all other metrics, its dispersion remained relatively low even at high percentiles, indicating consistent computational performance despite stochastic variation in reconstruction outcomes.

### Probability

We estimated the probability of a model being classified as MROR or MaROR based on its configuration and additionally conditioned this probability on both configuration and cloud evaluation. The highest probability (45%) was observed for the configuration consisting of 92 photos per set, one mask per image, masking applied to key points, and highest accuracy settings. Among the top 50 configurations with the highest probabilities (Table 4), 47 had applied masks to key points and 39 included ≥90 photos, highlighting a consistent pattern favoring high photo density combined with detected feature masking.

### Post Hoc Photo Number with Masks Settings

**Fig. 33:**
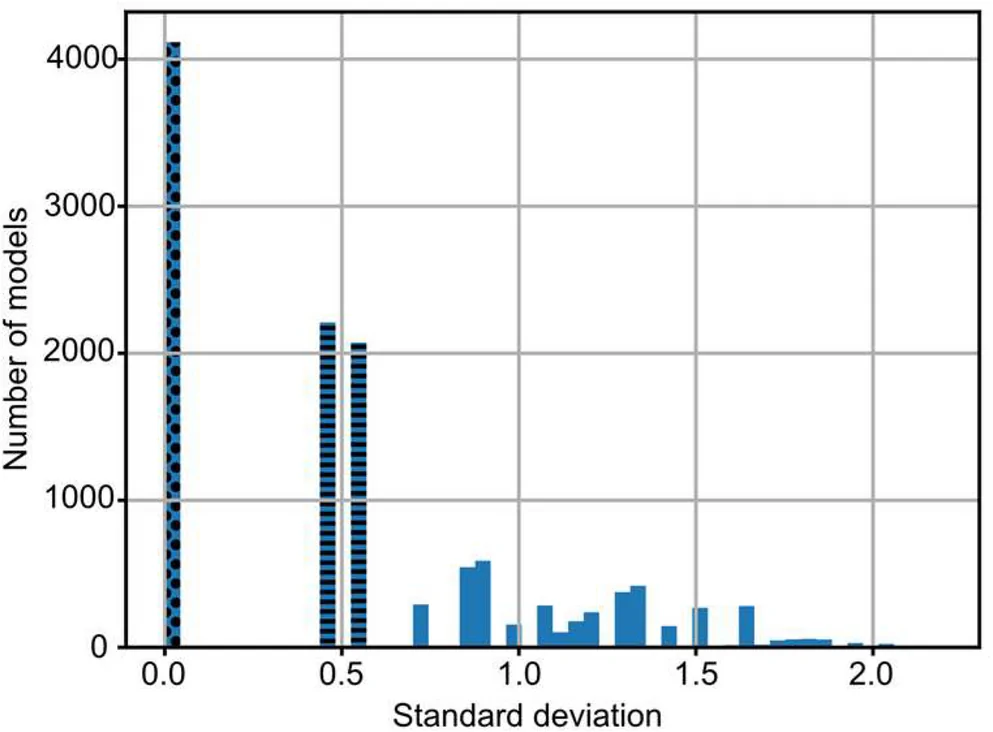
Histogram of standard deviation in variability points across groups of five models with identical configurations. Dotted bars represent groups with no variation. Horizontal line bars indicate groups with variations attributed to inter-user differences in criteria, while flat bars denote groups with significant variations in their results

**Fig. 34:**
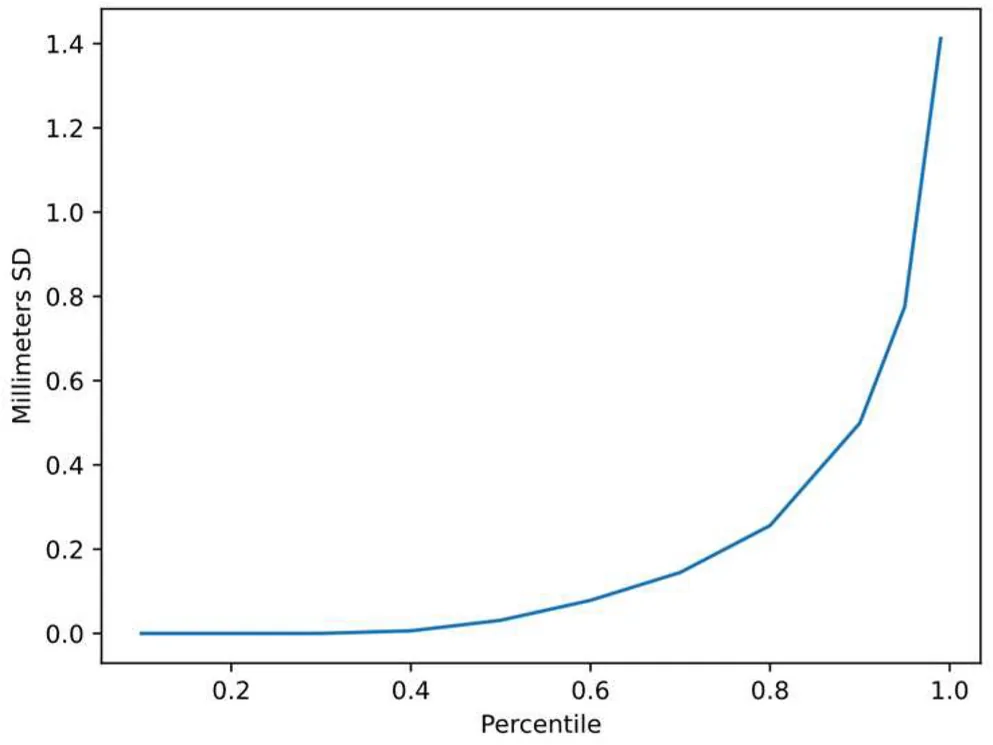
Standard deviation (SD) of RMS point error per matched feature in millimeters. Noticeable variability emerged at the median (SD = 0.03 mm), increasing to 0.499 mm at the 90th percentile and 1.411 mm at the 99th percentile

**Fig. 35:**
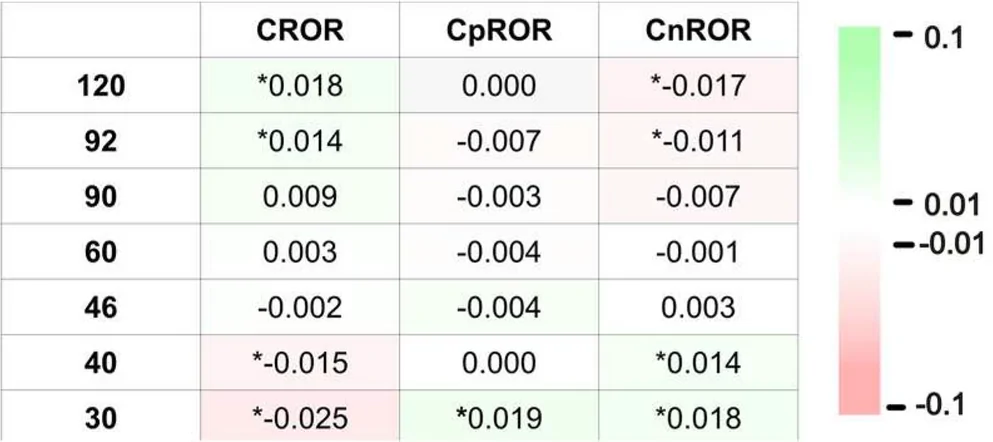
Adjusted standardized residuals of correlations between brain tie point cloud evaluations and photo number configurations only in the mask applied to detected features subgroups. Asterisks indicate significant values. p < 0.001. Cramer’s V = 0.13

Based on the previous observation, we performed a focused quantitative analysis of photo count within the subgroup using detected feature masking (Fig. 33, 34, 35). This analysis revealed a statistically significant association (*p* < 0.001) with a moderate effect size (Cramer’s V = 0.135). The strongest positive contributors to CROR outcomes were configurations with 120 and 92 photos (adjusted standardized residuals: +0.018 and + 0.014, respectively), whereas configurations with 40 and 30 photos showed the strongest negative contributions (− 0.015 and − 0.025, respectively).

**Table 4:** Configuration of top 50 MROR/MaROR probability

Photo number	Mask density	Mask setting	Accuracy	Satisfactory cloud probability	Satisfactory model probability
92	1/01	K	hst	0,75	0,45
90	1/01	K	hgh	0,53	0,35
92	1/01	K	low	0,73	0,35
120	1/01	K	hst	0,58	0,35
120	1/14	K	hst	0,82	0,35
90	1/01	K	hst	0,65	0,32
90	1/07	K	med	0,81	0,32
120	1/07	K	hgh	0,68	0,32
120	1/14	K	hgh	0,81	0,32
120	1/28	K	hgh	0,72	0,32
60	1/28	K	low	0,92	0,3
90	1/14	K	hst	0,8	0,3
90	1/14	K	med	0,70	0,3
90	1/28	K	med	0,66	0,3
92	1/01	K	hgh	0,57	0,3
92	1/01	K	med	0,63	0,3
92	1/07	K	hst	0,70	0,3
120	1/01	K	low	0,6	0,3
120	1/28	K	med	0,63	0,3
60	1/01	K	med	0,57	0,27
60	1/07	K	low	0,64	0,27
90	1/01	K	med	0,61	0,27
90	1/01	K	low	0,55	0,27
92	1/01	N	med	0,68	0,27
92	1/14	K	hgh	0,68	0,27
92	1/14	K	med	0,64	0,27
92	1/28	K	hst	0,73	0,27
92	1/28	K	hgh	0,64	0,27
92	1/28	K	med	0,68	0,27
92	1/28	K	low	0,91	0,27
120	1/01	N	med	0,84	0,27
120	1/01	K	hgh	0,55	0,27
120	1/01	K	med	0,68	0,27
120	1/07	K	med	0,73	0,27
120	1/14	K	med	0,68	0,27
40	1/01	K	med	0,5	0,25
46	1/01	K	med	0,58	0,25
46	1/07	K	hst	0,76	0,25
46	1/07	K	med	0,71	0,25
46	1/14	K	hst	1	0,25
46	1/14	K	low	0,83	0,25
46	1/28	K	med	0,83	0,25
60	1/01	K	low	0,58	0,25
60	1/07	K	hst	0,90	0,25
60	1/14	K	hst	1	0,25
60	1/14	K	med	0,76	0,25
60	1/28	K	med	0,76	0,25
90	1/01	N	hst	0,76	0,25
90	1/07	K	hst	0,76	0,25
90	1/14	K	hgh	0,71	0,25

## Discussion

The first noteworthy result is the variability in photo capture during our semi-automated process. In this system, a turntable rotates automatically, while the user manually takes a photo at each rotation. Although the goal is to capture 120 photos per set, this number is not always achieved due to human error. Photos may be missed if the user fails to take them before the turntable completes its next rotation, or multiple photos may be taken inadvertently. Additionally, it is common to end up with blurry images when the shutter is triggered while the turntable is still rotating. Ultimately, capturing such many photos consecutively is a monotonous task that demands continuous attention for at least 10 min.

In a fully manual system, where the user rotates the object of interest and takes the photos themselves, there is the advantage of greater precision in photo capture. However, the time required can vary depending on the user’s experience, and maintaining laboratory asepsis in pathology often requires more than one person. Ideally, a fully automated system, where a machine rotates the object and captures the photos, would address these issues.

In parallel with the work presented in this article, our laboratory has been prototyping a machine with these capabilities. While there is a significant improvement in photo quality and a reduction in capture time, the main challenges lie in training personnel to use the system and ensuring the technical maintenance of the equipment.

Although the average duration of the SfM process is 1.07 min, as we will see later, this can vary significantly depending on the configurations. Notably, there is a striking difference of nearly 2.7 times between the mean and the median, indicating the presence of significant time outliers that skew the average. In extreme cases, some models take an indefinite amount of time to align, as demonstrated by the longest alignment recorded, which took nearly 400 times the average duration.

Users should exercise caution when leaving alignment processes unsupervised, as the system may enter these prolonged alignment scenarios. Implementing a feature in the software that automatically cancels or restarts the process after a predefined time could be beneficial to avoid such situations.

The two types of qualitative assessments proposed in this article, evaluating the alignment “ring” and assessing the cloud shape, proved to be effective, with most cases showing agreement between two distinct evaluators. The alignment assessment demonstrated slightly higher evaluation concurrence (71.91%) compared to the cloud assessment (68.41%), suggesting that the former may be more intuitive and easier to learn.

When considering all possible configuration combinations, only 1 in 5 models aligned satisfactorily. In the remaining cases, results included either disagreement between evaluators, consensus that the model did not align completely, or a clear misalignment. This serves as an initial indication that certain configurations may yield better qualitative outcomes than others.

When looking for quantitative measurements of SfM clouds we see that CROR models had a significantly higher number of matching features and highest number of valid projections per photo; and shortest SfM processing time than other evaluations. These same properties were shared with their homologous of alignment evaluation: AC.

There is a strong correlation between the two qualitative assessment methods. The most robust and practical correlation is observed between CROR and AC; in other words, both methods tend to classify the same model as satisfactory. The correlation between CnROR and AI is equally practical but unfortunately weaker, meaning that if one method determines the model to be rendered incorrectly, the other method often suggests the same outcome, though it is more prone to disagreement.

The strength of the correlation diminishes when comparing other homologous ratings between the two methods. However, these comparisons are less useful in practice.

Due to computational and time constraints, we completed the full reconstruction pipeline (including mesh and texture generation) for only one representative model from the five renderings generated for each configuration. In the qualitative evaluation, the probability of successful rendering was initially consistent with results from the point cloud stage (approximately 1 in 5). However, this proportion decreased slightly after reassessment by a more experienced author (approximately 1 in 6). This suggests that, although the parameters used to define a satisfactory model are broadly reliable, expert evaluation remains important for accurately identifying successful reconstructions.

In the quantitative analysis, MROR and MaROR exhibited similar behavior across all parameters, with only a minor difference in RMS distance to the reference object (~ 0.3 mm on average), with MaROR showing slightly lower values. This minimal difference suggests that separating these categories may not be necessary.

When combined into a single group, MROR/MaROR exhibited the highest number of matched features and valid projections per photo, consistent with their corresponding CROR and AC classifications. Notably, MROR/MaROR also demonstrated lower RMS point error per detected feature compared to MpROR and MnROR. This indicates that final model evaluation may provide a more reliable indicator of geometric accuracy than either point cloud or alignment assessments alone.

A very strong correlation was observed between the alignment assessment and final model outcome, with a slightly stronger correlation identified between point cloud evaluation and final model quality. When MROR and MaROR were combined into a single group, these correlations increased further. This suggests that the presence of a CROR cloud is strongly associated with successful reconstruction, with a 62.3% probability of obtaining an MROR or MaROR when both evaluators agree on a CROR classification.

However, this relationship is not absolute. Some apparently high-quality clouds may still fail to produce accurate final models, while, conversely, some clouds initially classified as suboptimal can yield acceptable reconstructions. These findings indicate that additional stages of the pipeline, particularly dense cloud generation, meshing, and texturing, play a critical role in determining final model quality.

Our hypothesis regarding the photo-taking angle was confirmed. Renderings taken at a 30-degree angle and 30 cm height (configurations B and C) were indeed more correlated with a correct cloud, while those taken at the same height without inclination (configurations A and D) showed an inverse correlation with a correct cloud. We believe the primary reason for this is that configurations A and D offer less surface area for the software to generate key points.

Across photo-taking angles, MROR/MaROR behaved similarly to CROR in all quantitative parameters. Configurations A and D tended to show a slightly lower RMS of point error per matched feature but required slightly longer processing times compared to B and C. Additionally, RMS vertex error relative to ground truth was comparable between A and D and was approximately half that observed in B and C.

These findings suggest that, although configurations A and D are less likely to produce a satisfactory complete model, when successful, they yield more geometrically reliable reconstructions than those obtained from configurations B and C.

While both A and D capture the same brain data, albeit rotated 180 degrees, one might initially assume this information to be redundant. However, since A and D have lower success rates than B and C, it may be prudent to capture both, as one could serve as a backup if the other fails.

**Fig. 36:**
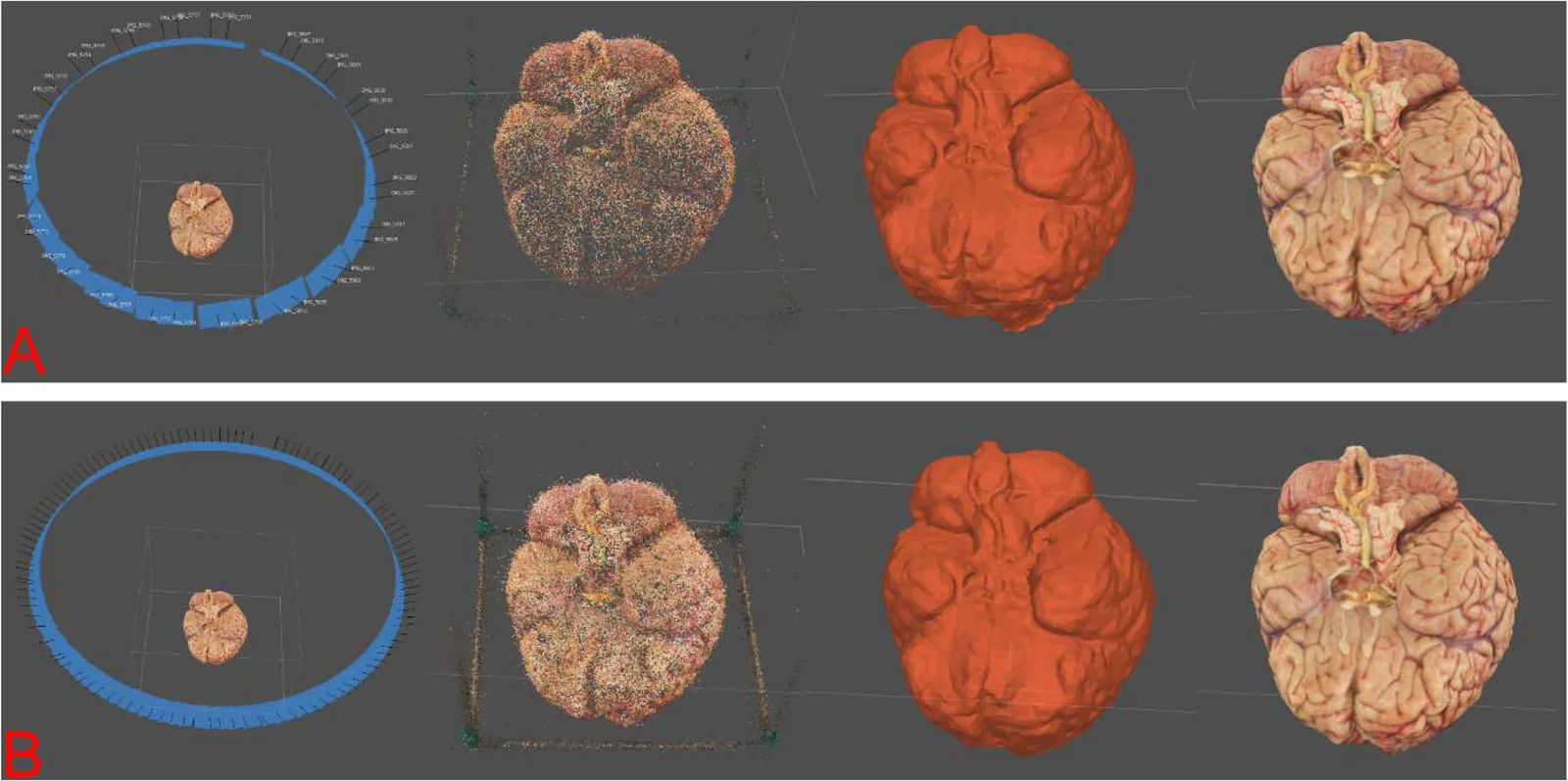
Comparison of reconstructions using different photo counts. Row (**A**) shows models generated from 40 photos, and row (**B**) from 120 photos both on medium accuracy and C angle. From left to right: (1) camera capture angles (blue rectangles) surrounding the model; (2) matched-feature sparse point cloud, where the 40-photo configuration shows lower density and detail; (3) mesh view; and (4) textured model. Despite differences in sparse cloud density, no visible differences are observed between configurations in mesh or texture quality

One of the most notable findings of this study is that the relationship between photo count and CROR outcomes is negligible when masking is not specified. Under these conditions, the probability of achieving a CROR remains essentially unchanged whether 120 or 30 photos are used. However, when masking is applied to detected features, this relationship shifts from negligible to moderate, with higher photo counts (particularly 92 and 120 images) associated with improved outcomes. Furthermore, although matched feature density increases with higher photo counts, this does not translate into substantial differences in the final mesh quality when comparing high count versus low count photo configurations (Fig. 36).

Importantly, this improvement comes at a substantial computational and acquisition cost. Reducing the number of photos from 120 to 40 decreases the average processing time from 2.78 min to 1.00 min, approximately a threefold reduction. In addition, given an acquisition time of ~ 2.5 s per image, reducing the photo count by 66% translates to ~ 13 min saved per dataset during image capture alone. These findings highlight a critical trade-off between reconstruction performance and efficiency, emphasizing the importance of optimizing both photo count and masking strategy.

This reduction also leads to savings in mask creation, which could otherwise require approximately 13 h of manual labor if masks were created for every photo (at an average of 2.5 min per mask by experienced personnel). Additionally, it implies reduced computational storage requirements. Unfortunately, our study may have encountered a floor effect, making it impossible to determine the minimum number of photos needed per set without compromising the likelihood of creating an CROR. A follow-up study to address this question would be a valuable continuation of the present work.

Lastly, no advantage was found in increasing the photo density in the frontal and occipital poles, indicating that such adjustments do not improve the likelihood of obtaining a correct result.

It was observed that applying the mask to key points has a strong positive correlation with achieving an CROR, and there is no difference in processing time compared to not applying masks. Conversely, not using masks does not correlate with obtaining a satisfactory cloud. When the type of mask application is unspecified, there is a weak correlation between the number of masks per image and no significant difference in rendering time. However, in the subgroup where masks are applied exclusively to detected features, there is a moderate positive correlation between creating a mask for each image and obtaining a satisfactory cloud.

We recommend initially attempting rendering without masks to save the time required for their creation. If alignment results are unsatisfactory, we suggest creating one mask per image and applying these to detected features. There seems to be an all-or-nothing effect, where benefits are observed only when either a mask is created for every image, or no masks are created at all. Creating an intermediate number of masks offers no advantages.

In our results, there was a negative correlation between the use of applying masks to tie points and achieving satisfactory alignment. However, it is important to note that in our study, this option was not used as recommended. The intended approach for applying masks to tie points involves taking a photo of the background without the object and then proceeding to take photos of the object of interest with the same background. When processing the photos, a single mask can be created covering the entire background photo without the object, ensuring that any key points identified in that background are ignored in subsequent photos.

The theoretical advantage of this method is that only one mask is needed for the entire set, significantly reducing time and effort in mask creation. It would be prudent to conduct further studies to determine if our results hold when applying masks to tie points as recommended.

It is worth noting that, for this study, the masks were created by strictly delimiting the edge of the object of interest, which is a labor-intensive process. However, with updates to Metashape that allowed us to visualize the key points generated in each photo, we observed that creating a mask as a rectangle enclosing the object of interest appears to have no interference with key point generation. This approach has the added advantage of being significantly less time-consuming to create masks.

Further studies are needed to determine whether these findings hold when using simplified masking strategies. Our results suggest that most matched features are observed in only two to three images, indicating that Metashape primarily establishes correspondences between adjacent photographs. Consequently, sparse masking strategies (e.g., one mask every seven images or fewer) may be ineffective or outside the practical scope of the alignment process. Future work should therefore evaluate denser masking approaches, such as applying masks every two to three images.

Additionally, given that masking improves both the probability of successful reconstruction and model quality, efforts should be directed toward automating this step, particularly through AI-based image segmentation to generate masks efficiently and consistently.

Regarding both photo count and masking strategy, no significant differences were observed between MROR/MaROR and CROR across quantitative metrics, including RMS vertex error relative to ground truth. However, both MROR/MaROR and CROR consistently exhibited higher numbers of matched features, photo alignment ratios, and valid projections per photo compared to CpROR and CnROR.

Our results suggest that the best cloud outcomes are obtained when selecting either the Highest or Medium precision options, skipping the High precision setting. Despite conducting multiple post-hoc analyses within subgroups, we did not find results that could explain this bimodal distribution. Now, it seems that certain configurations, currently unknown, benefit from the highest precision, while others, also unknown, benefit from medium precision. Further studies are needed to clarify what these configurations might be.

In the meantime, we recommend using Medium precision to process photos, as it takes 2 to 3 times less time than the Highest precision option.

The highest, high, and medium accuracy settings demonstrated comparable reliability, showing similar values in matched features, RMS point error per matched feature, number of valid projections, and RMS vertex distance relative to reference object. In contrast, this reliability decreased at low and lowest accuracy settings, where performance across these metrics deteriorated.

A limitation of this study lies in the construction of the reference models, which were generated through supervised photogrammetry to ensure high quality but remain inherently subject to observer bias. Consequently, measurements of RMS vertex distance relative to the reference object should be interpreted with caution. An ideal approach would involve comparison against a true gold-standard modality, such as magnetic resonance imaging (MRI); however, practical constraints in time and cost limited its use in this study. Future work should incorporate such reference standards to improve the robustness and objectivity of accuracy assessments.

Although most of the time repeating the alignment five times within the same configuration shows no variability in the cloud evaluation, 1 in 3 groups does exhibit significant variability despite identical configurations. This was also seen in the quantitative data where only the valid projections per matched feature didn’t show variance between the different renders. This suggests that Metashape introduces a certain degree of randomization, leading to different results with the same input.

Based on this, we recommend that, in cases where the initial alignment results in unsatisfactory clouds, it may be beneficial to repeat the alignment in search of a suitable cloud. This is particularly important when access to the object of interest for retaking photos is no longer possible, as was the case in our study.

## Conclusions

A total of 12,600 configuration combinations and 63,000 alignments were conducted, allowing us to determine that alignments in which the point cloud accurately represents the reference object exhibit a moderate correlation with a final model deemed satisfactory. Based on our findings, we recommend an optimized imaging parameters consisting of four sets of photographs: two sets taken at the anatomical position at the same level as the brain and approximately 30 cm above it, with a camera tilt of approximately 30 degrees; and two additional sets with the same configurations but with the brain’s basal side facing upwards.

The brain should ideally be rotated 3° between each photograph, yielding 120 images per set. However, when time constraints are a priority, reducing the acquisition to 40 images per set is a viable alternative, resulting in approximately a one-third reduction in the probability of obtaining a satisfactory model, while substantially decreasing both image acquisition and processing time.

For initial processing, we recommend generating the 3D models in Agisoft Metashape without applying masks and using a medium-accuracy setting. However, if the alignment fails, an alternative approach involves applying one mask per image, delineating the brain’s borders, and utilizing key point-based masking.

To our knowledge, this is the first study to systematically analyze both imaging and software configurations for optimizing 3D photogrammetry of brain specimens using a turntable. Furthermore, it provides specific technical recommendations for neuroanatomical photogrammetry. Future studies are needed to determine the minimum number of images required to maintain model quality without compromising accuracy.

## Supplementary Information

Below is the link to the electronic supplementary material.

Supplementary Material 1

## Data Availability

The brain photogrammetry images, an Excel file containing render data, and the Python scripts used in this study are provided through this link: https://drive.google.com/drive/folders/1OiKuWlGidyTkaYarhRJcYvk8r4SlaiYs? usp=sharing . Due to storage limitations, the Metashape files containing the rendered models are available upon request. To obtain these files, please contact the corresponding authors, C.A.R. or A.V., via email.

## Abbreviations

1/1: One mask on each photo
1/7: Ones mask every seven photos
1/14: One mask every 14 photos
1/28: One mask every 28 photos
AC: Complete Alignment
AI: Incorrect Alignment
AP: Partial Alignment
CnROR: Cloud does not Represents Object of Reference
CpROR: Cloud partially Represents Object of Reference
GNA: Antioquia Neuroscience Group
MaROR: Model approximately Represents Object of Reference
MnROR: Model does not Represents Object of Reference
MpROR: Model partially Represents Object of Reference
MROR: Model Represents Object of Reference
SD: Standard Deviation
SfM: Structure from motion
RMS: Root Mean Square
