# Supplementary material for: Optimizing Photogrammetry Parameters for 3D Reconstruction of Fresh Brain Specimens
Source: Neuroinformatics. 2026 Jul 25;24(3):50. doi: 10.1007/s12021-026-09789-y (PMC13401559; doi:10.1007/s12021-026-09789-y)
Supplement: Supplementary file 1 — Supplementary Material 1 [file 12021_2026_9789_MOESM1_ESM.docx]

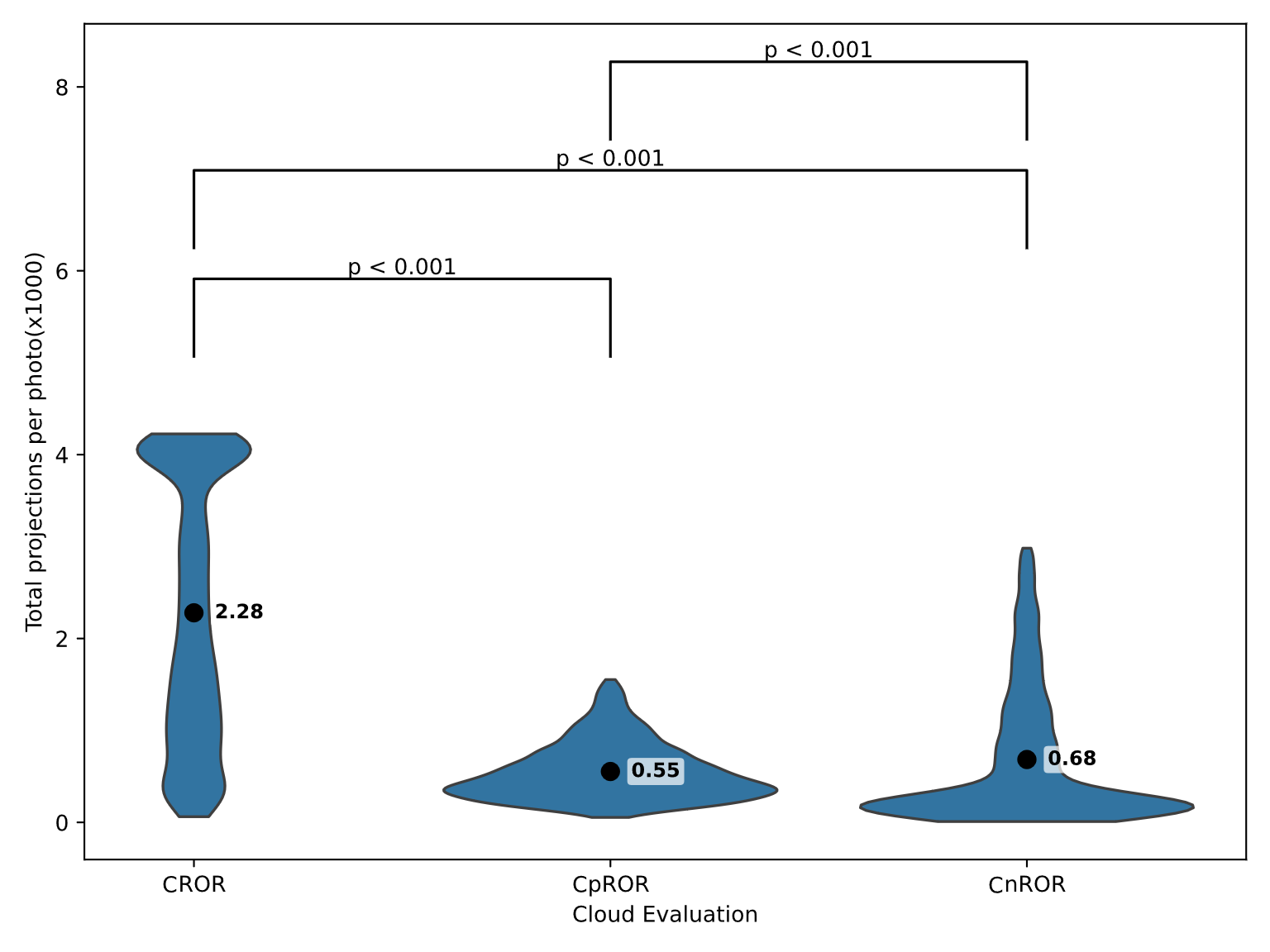

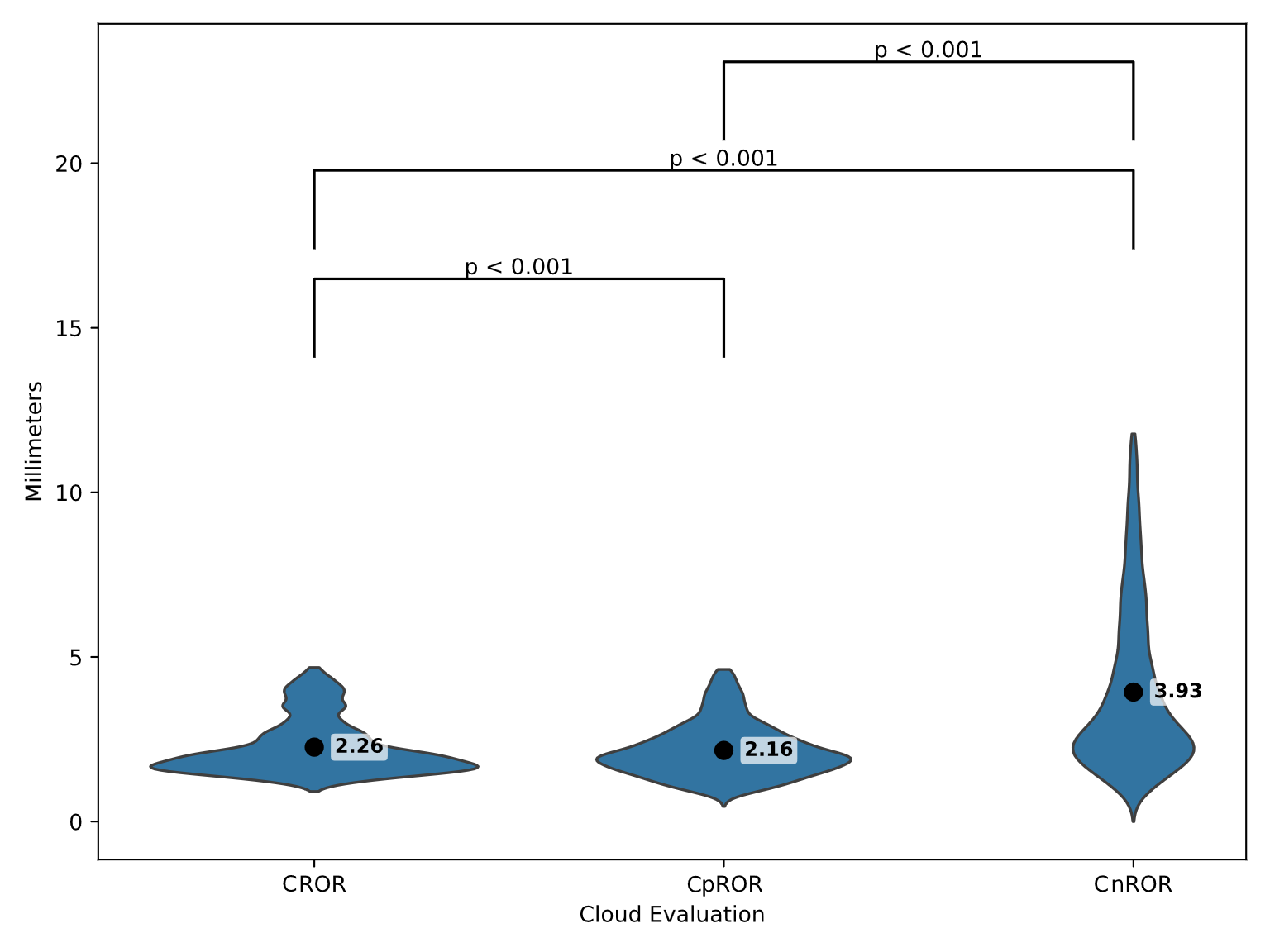


Fig. S4 Number of total projections per photo by cloud evaluation. CROR showed the widest distribution and highest values (mean = 2,280; median = 2,131; range = 62–4,225; SD = 1,425). CpROR and CnROR displayed narrower, pyramid-shaped distributions, with CpROR having a broader base. Their means were substantially lower (553 and 684, respectively), with ranges of 54–1,553 (CpROR) and 9–2,983 (CnROR), and lower medians (479 and 299) and variability (SD 312 and 737). Differences among all groups were statistically significant (p < 0.001).

Fig. S3 Number of valid projections per matched feature by cloud evaluation. All groups showed a median of 2 with nearly identical variability (SD = 0.42–0.44 for CROR, CpROR, and CnROR). Due to the high similarity of distributions, statistical comparisons between groups could not be meaningfully computed.

Fig. S2 RMS of point error per matched feature by cloud evaluation. CROR and CpROR showed nearly identical distributions, with similar means (2.26 vs. 2.16 mm), medians (1.98 vs. 2.00 mm), standard deviations (0.84 vs. 0.81 mm), and maximum values (~4.6–4.7 mm), differing mainly in minimum values (0.91 mm for CROR vs. 0.46 mm for CpROR). In contrast, CnROR exhibited a much broader distribution, ranging from 0 mm (models without matched features) to 11.77 mm, with a higher mean (3.93 mm) and greater variability (SD = 2.40 mm). Differences among all groups were statistically significant (p < 0.001).


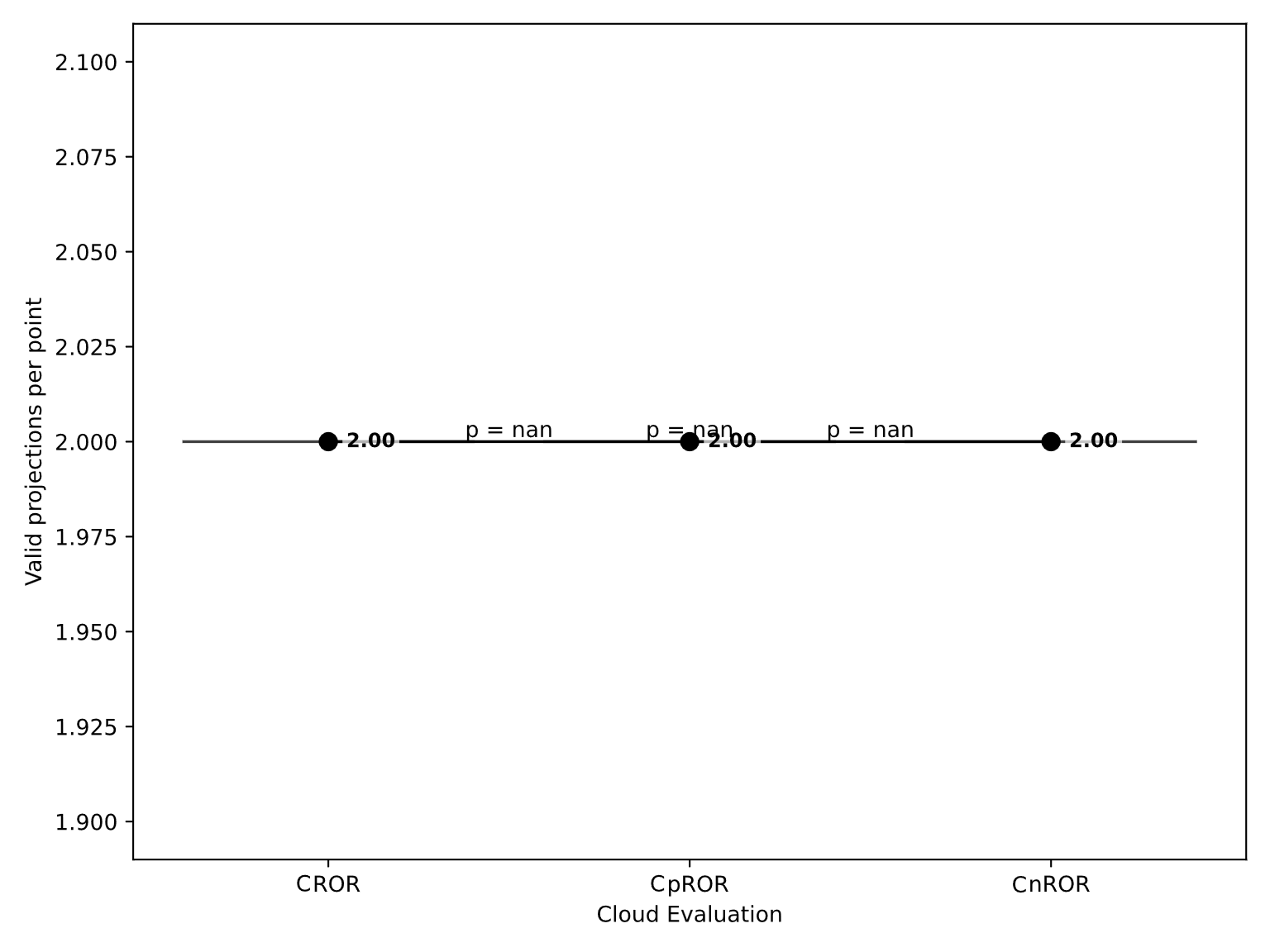

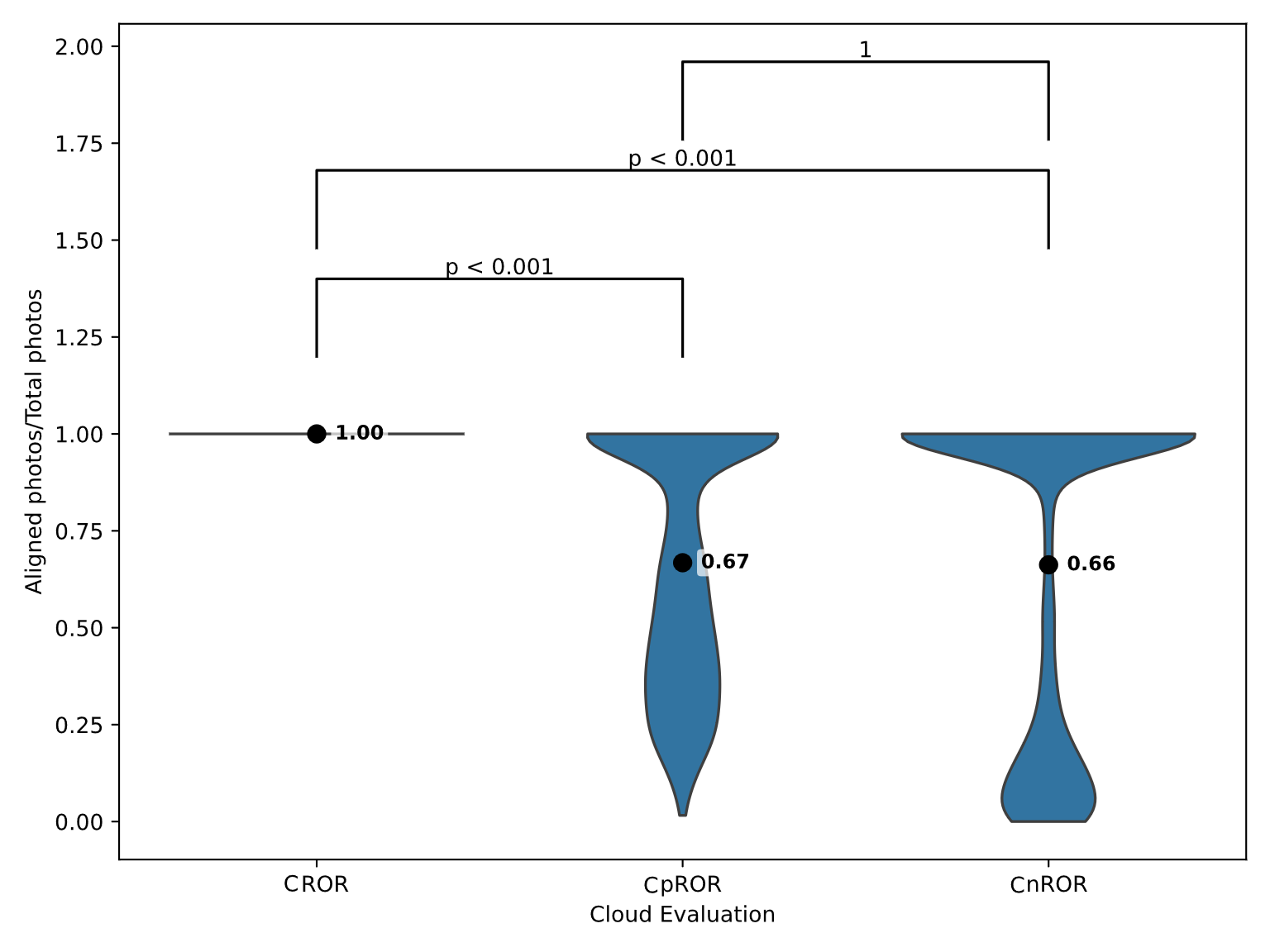


Fig. S1 Photo alignment ratio by cloud evaluation. CROR exhibited a near-perfect alignment ratio (mean = 1.0; SD = 0.01) and differed significantly from both CpROR and CnROR (p < 0.001). In contrast, CpROR and CnROR showed lower and comparable ratios (means = 0.66–0.67; p = 1), with bimodal distributions and greater variability (SD = 0.31 and 0.41, respectively).


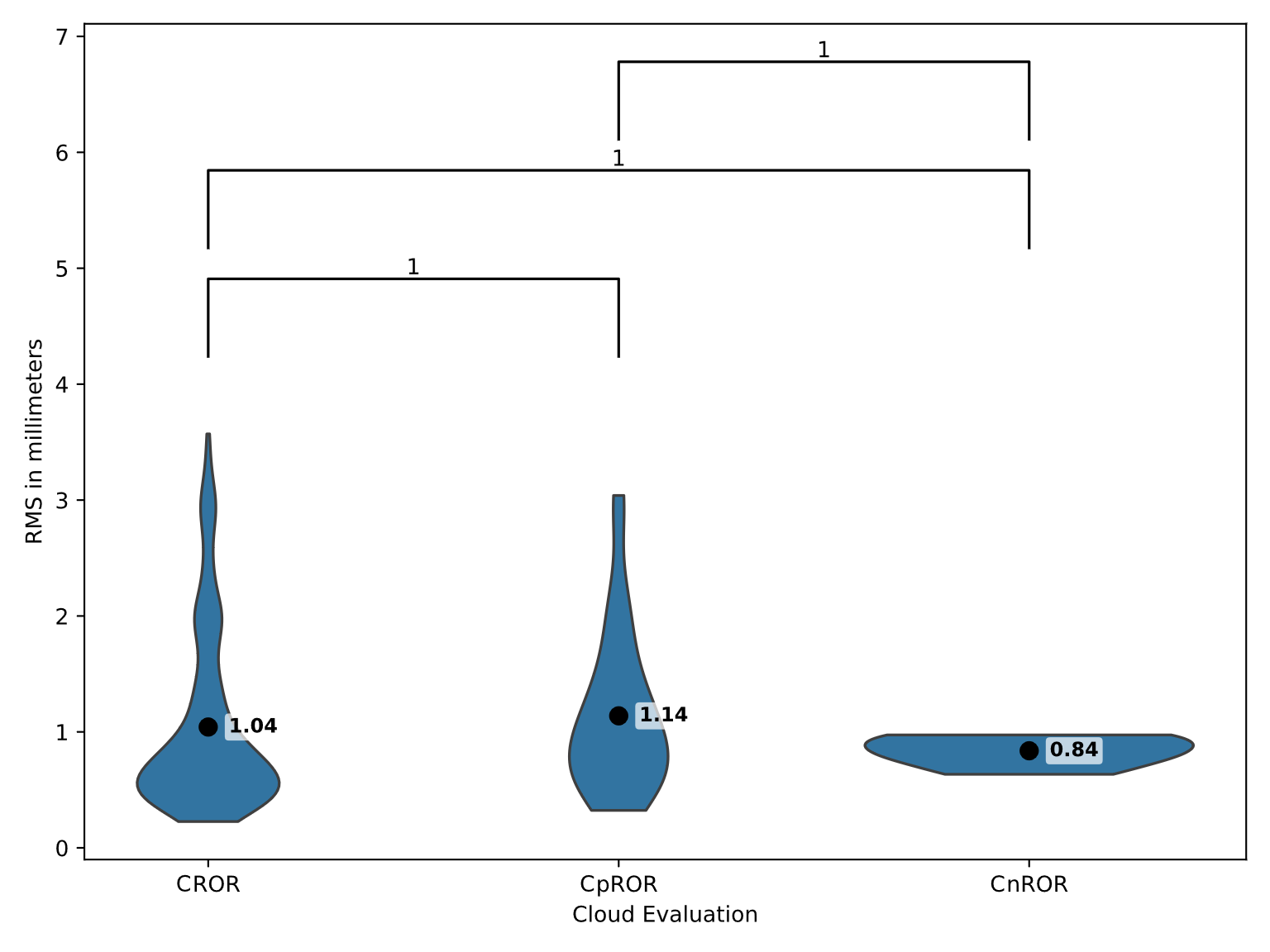

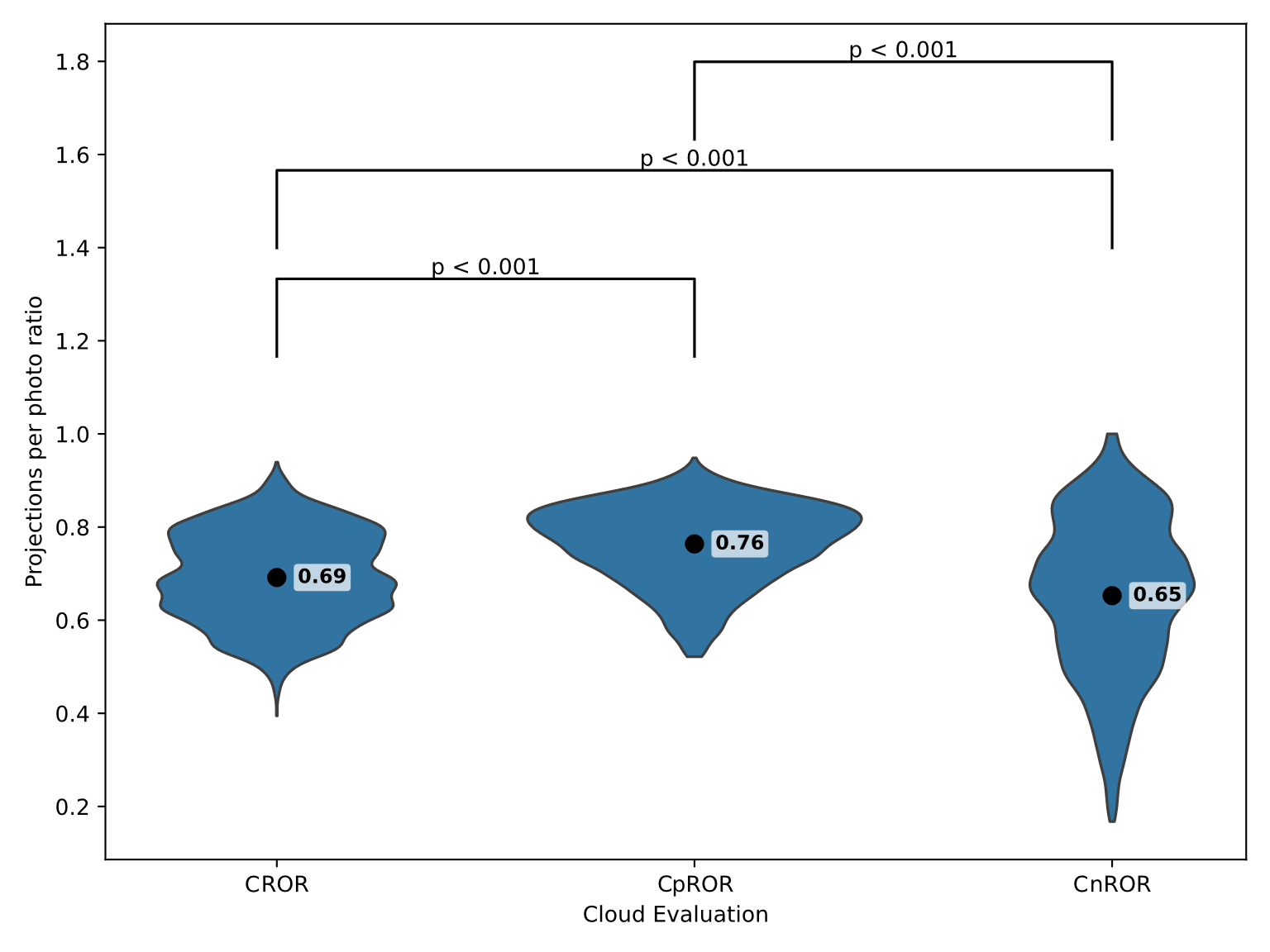


Fig. S6 RMS vertex distance to reference model by cloud evaluation. No statistically significant differences were observed among CROR, CpROR, and CnROR (all p = 1). CROR showed a mean of 1.04 mm (median = 0.72; SD = 0.77; range 0.14–3.57 mm), while CpROR had similar values (mean = 1.14 mm; median = 0.96; SD = 0.67; range 0.32–3.04 mm). CnROR exhibited a narrower distribution (mean = 0.84 mm; median = 0.83; SD = 0.14; range 0.63–0.97 mm), likely reflecting its small sample size.

Fig. S5 Valid-to-total projections ratio by cloud evaluation. CROR showed a mean and median of 0.69 (SD = 0.10; IQR: 0.62–0.77). CpROR had higher and more consistent ratios (mean = 0.76; median = 0.78; SD = 0.08; IQR: 0.71–0.83). In contrast, CnROR had similar central values to CROR (mean = 0.65; median = 0.67) but a wider distribution (SD = 0.17; IQR: 0.54–0.78). Differences among all groups were statistically significant (p < 0.001).


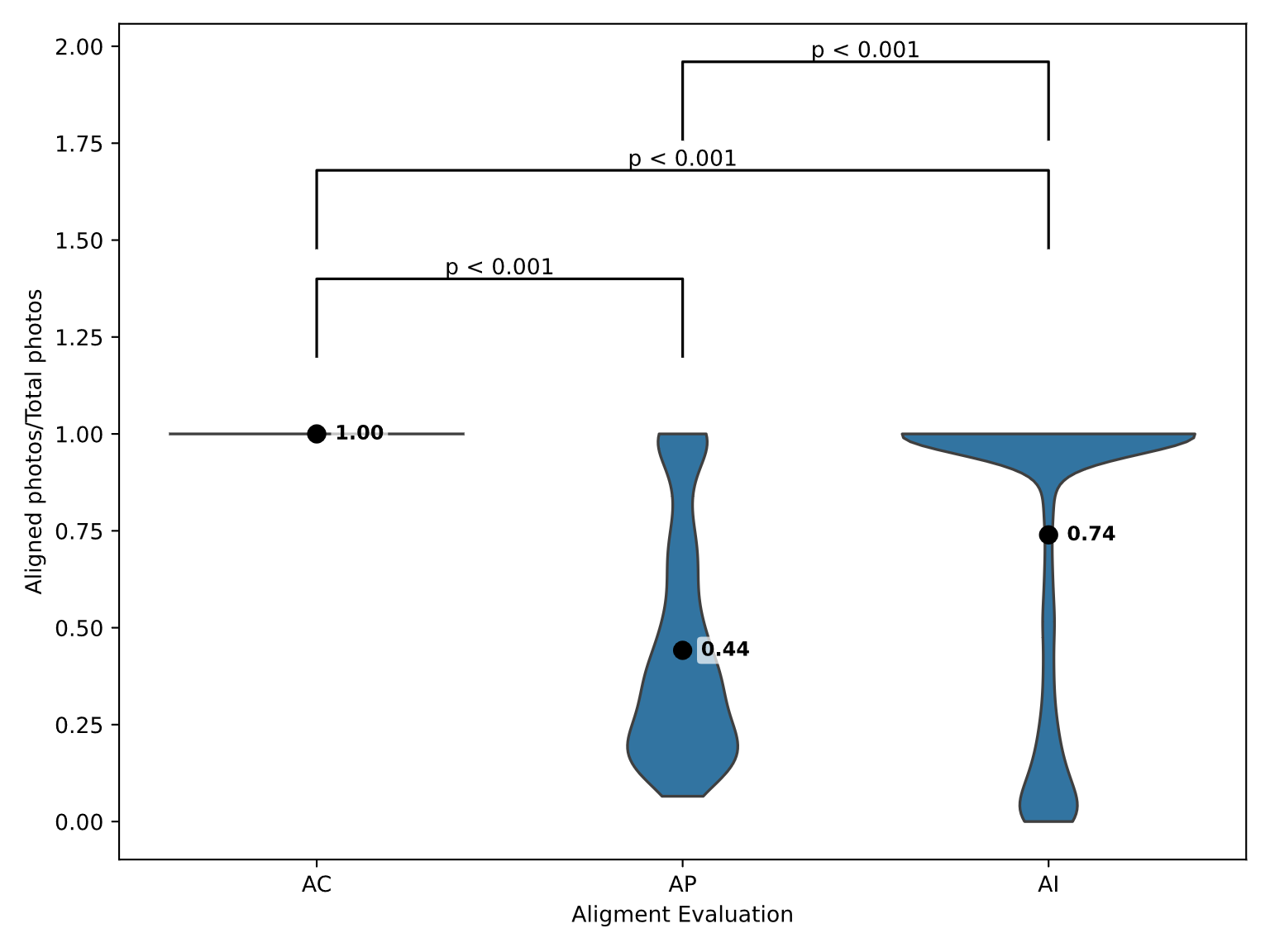

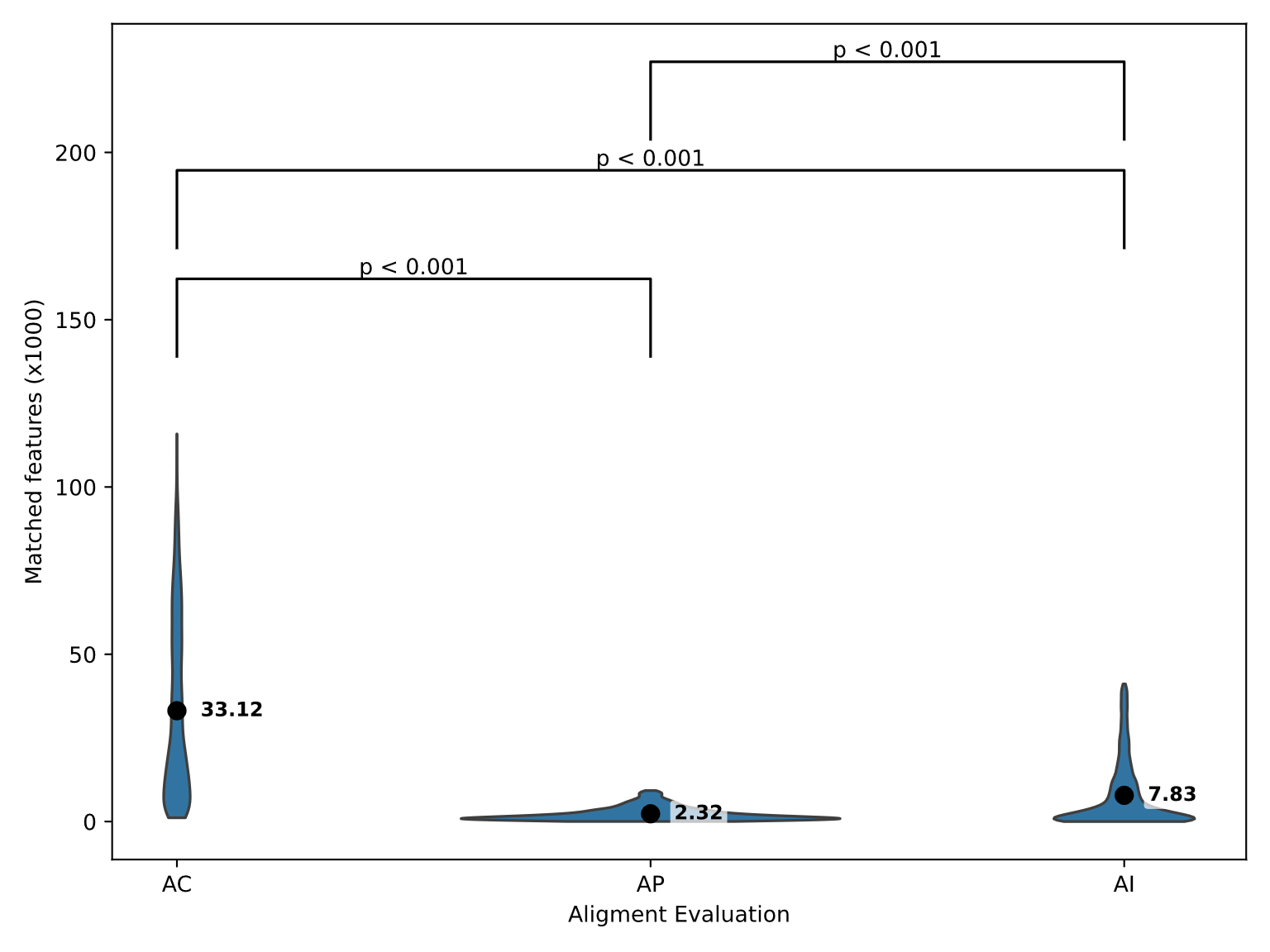


Fig. S8 Photo alignment ratio by alignment evaluation. AC showed perfect alignment (mean = 1.0; SD = 0). AP had substantially lower ratios (mean = 0.44; median = 0.36; SD = 0.29; range 0.07–1), while AI exhibited intermediate but highly variable values (mean = 0.74; median = 1.0; SD = 0.39; range 0–1). Differences among all groups were statistically significant (p < 0.001). Differences among all groups were statistically significant (p < 0.001).

Fig. S7 Number of matched features by alignment evaluation. AC showed the highest feature counts (mean = 33,123; median = 25,071; range 1,136–115,864). AP exhibited the lowest values (mean = 2,324; median = 1,603; minimum = 28), while AI presented intermediate values (mean = 7,832; median = 3,342), including a substantial proportion of models with no matched features. Differences among all groups were statistically significant (p < 0.001).


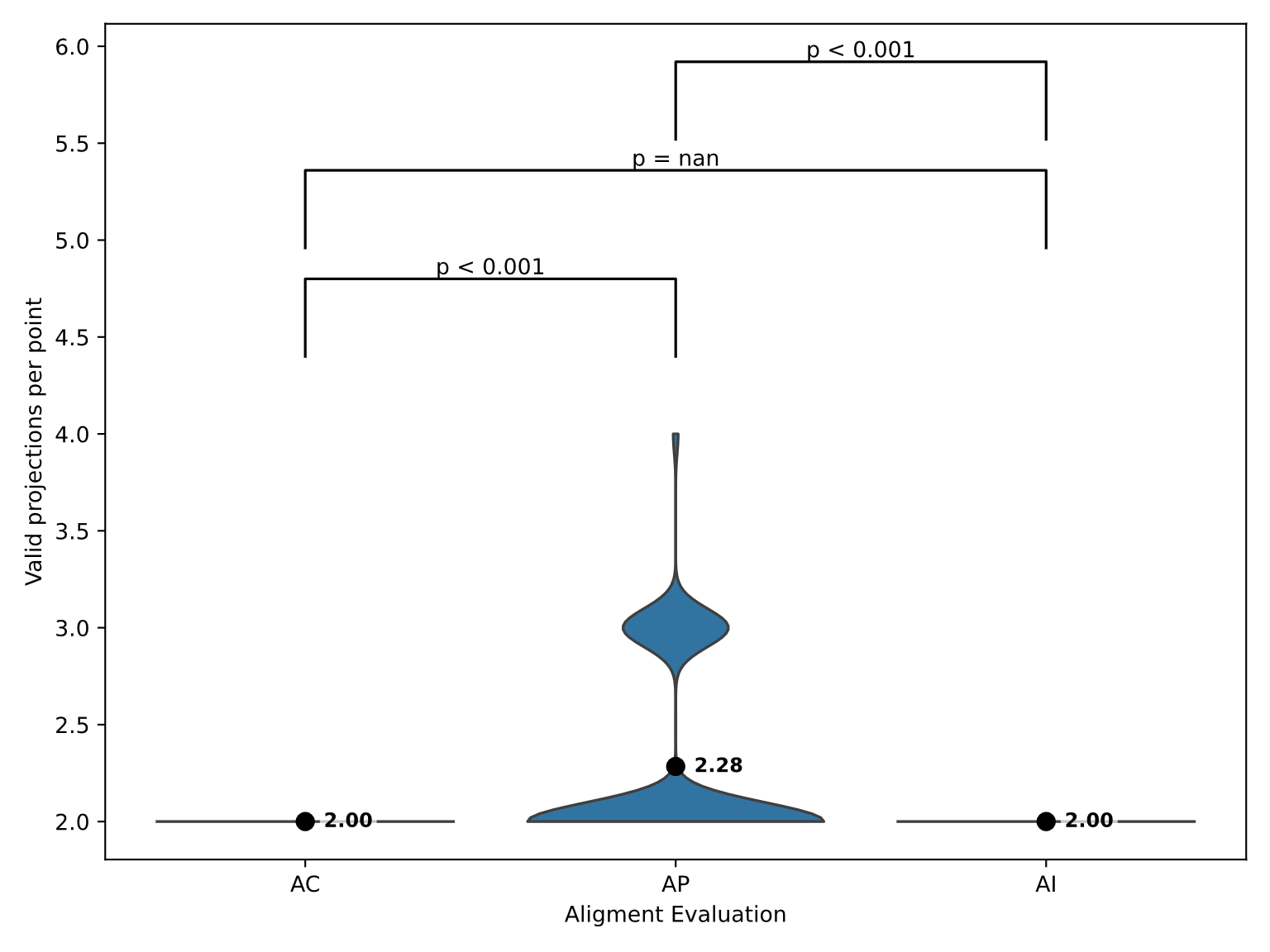

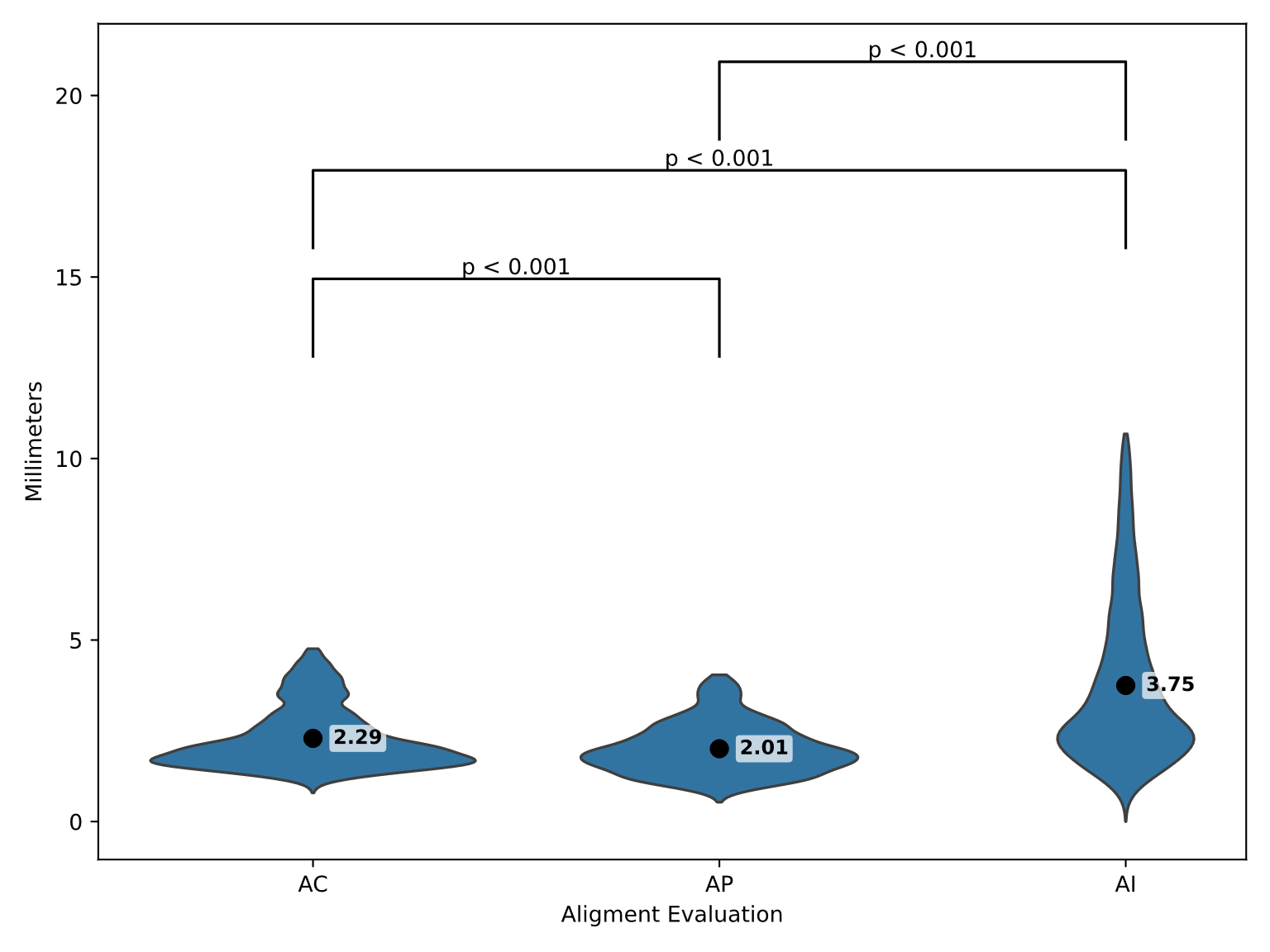


Fig. S10 Number of valid projections per matched feature point by alignment evaluation. AC and AI showed constant values (mean = median = 2; SD = 0; range = 2–2), whereas AP exhibited slightly higher and more variable values (mean = 2.28; median = 2; SD = 0.48; range 2–4). Differences between AP and both AC and AI were statistically significant (p < 0.001), while no comparison could be computed between AC and AI due to identical distributions.

Fig. S9 RMS point error per matched feature by alignment evaluation. AC and AP showed similar error distributions (AC: mean = 2.29 mm; median = 2.03; SD = 0.84; range 0.79–4.76 mm; AP: mean = 2.01 mm; median = 1.89; SD = 0.70; range 0.53–4.04 mm), whereas AI exhibited substantially higher and more variable errors (mean = 3.75 mm; median = 3.04; SD = 2.19; range 0–10.68 mm). Differences among all groups were statistically significant (p < 0.001).


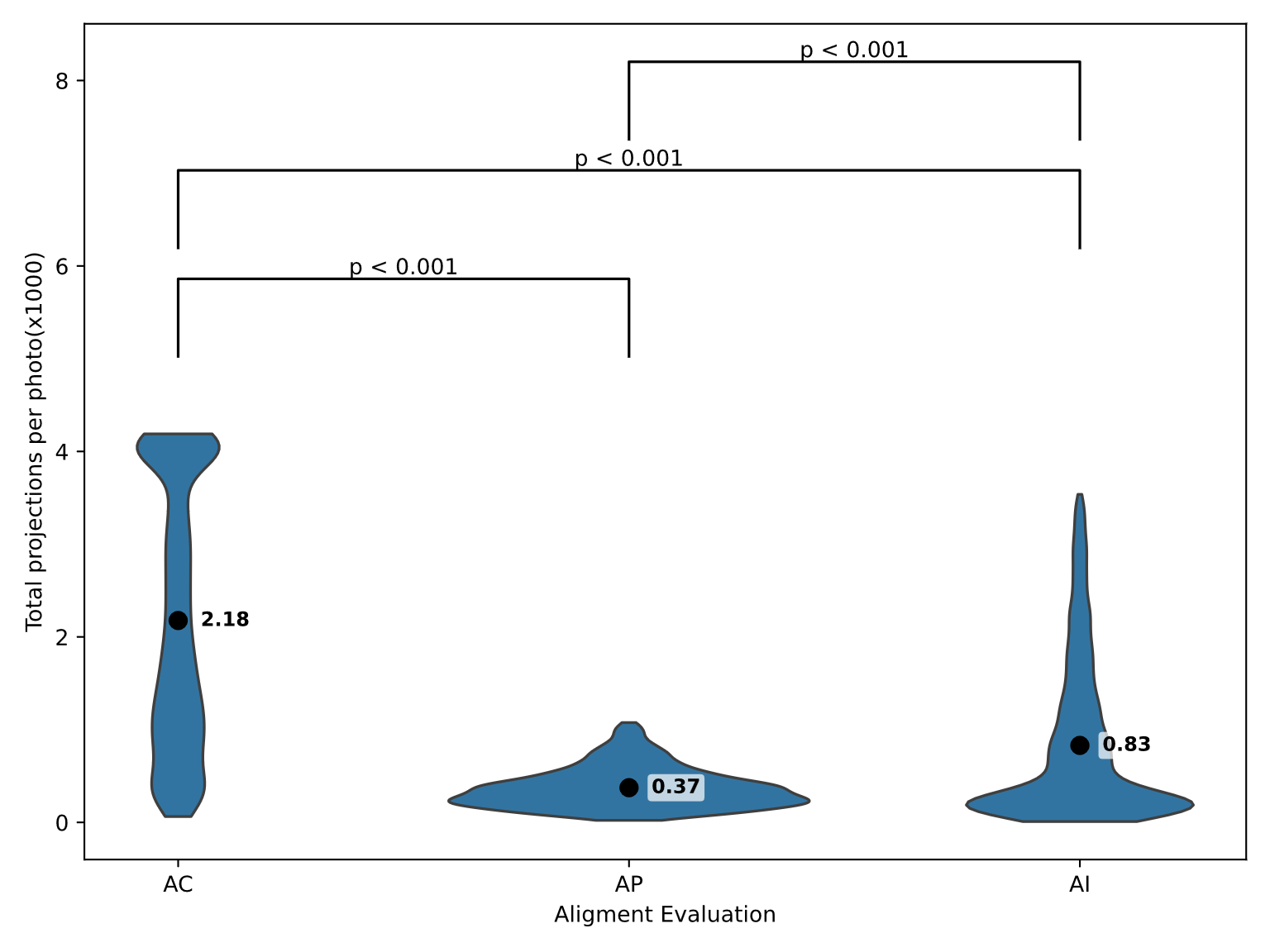

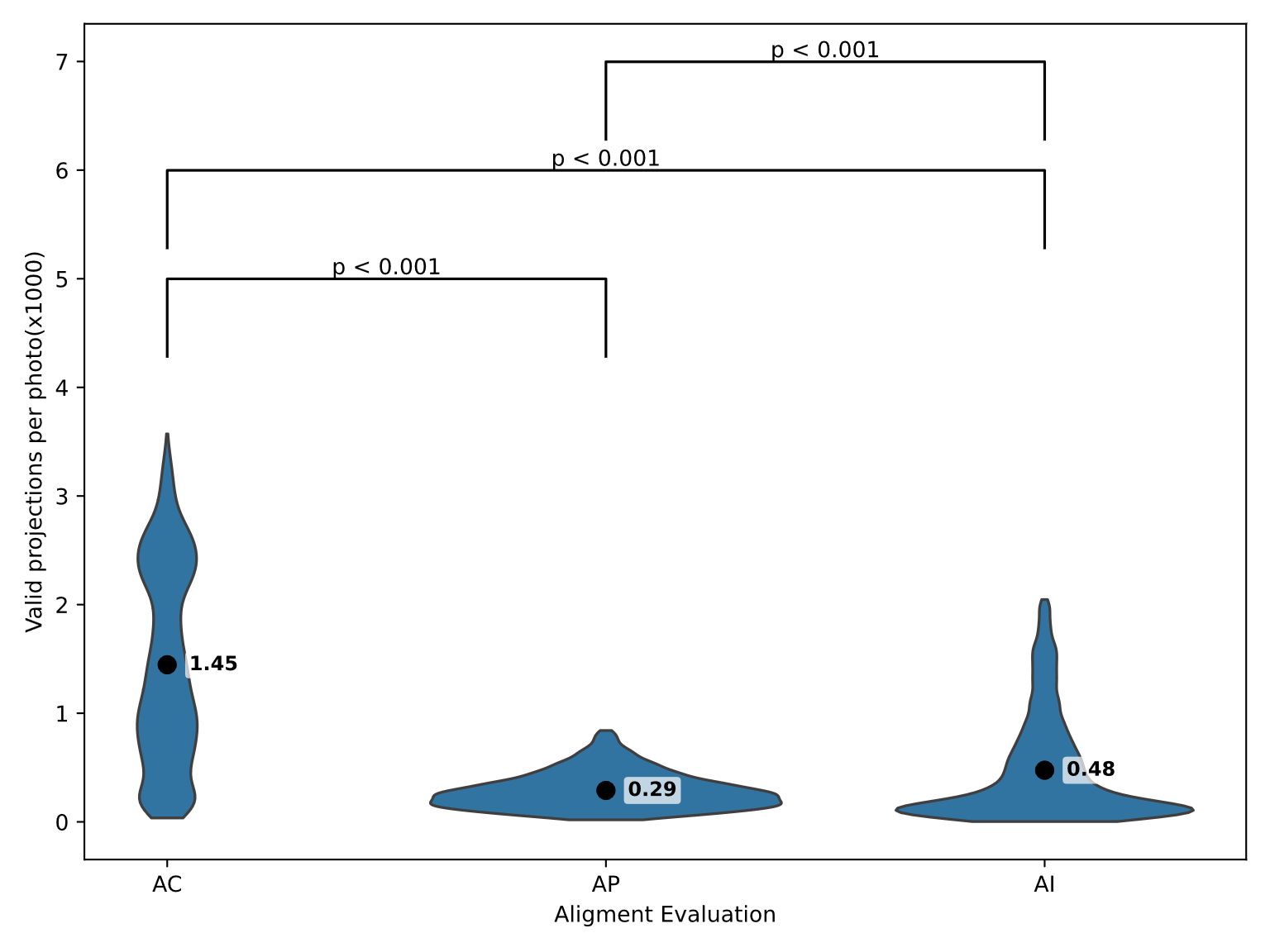


Fig. S12 Median number of total projections per photo by alignment evaluation. AC showed the highest values (mean = 2,177; median = 1,902.5; SD = 1,406; range 63–4,189), followed by AI (mean = 830; median = 444.5; SD = 855; range = 9–3,537), while AP had the lowest values (mean = 374; median = 333; SD = 221; range = 22–1,076). Differences among all groups were statistically significant (p < 0.001).

Fig. S11 Median number of valid projections per photo by alignment evaluation. AC showed the highest values (mean = 1,446; median = 1,302; SD = 906; range 36–3,571.5), followed by AI (mean = 475; median = 271; SD = 483; range = 3–2,046.5), while AP had the lowest values (mean = 289; median = 259; SD = 169; range = 19–841). Differences among all groups were statistically significant (p < 0.001).


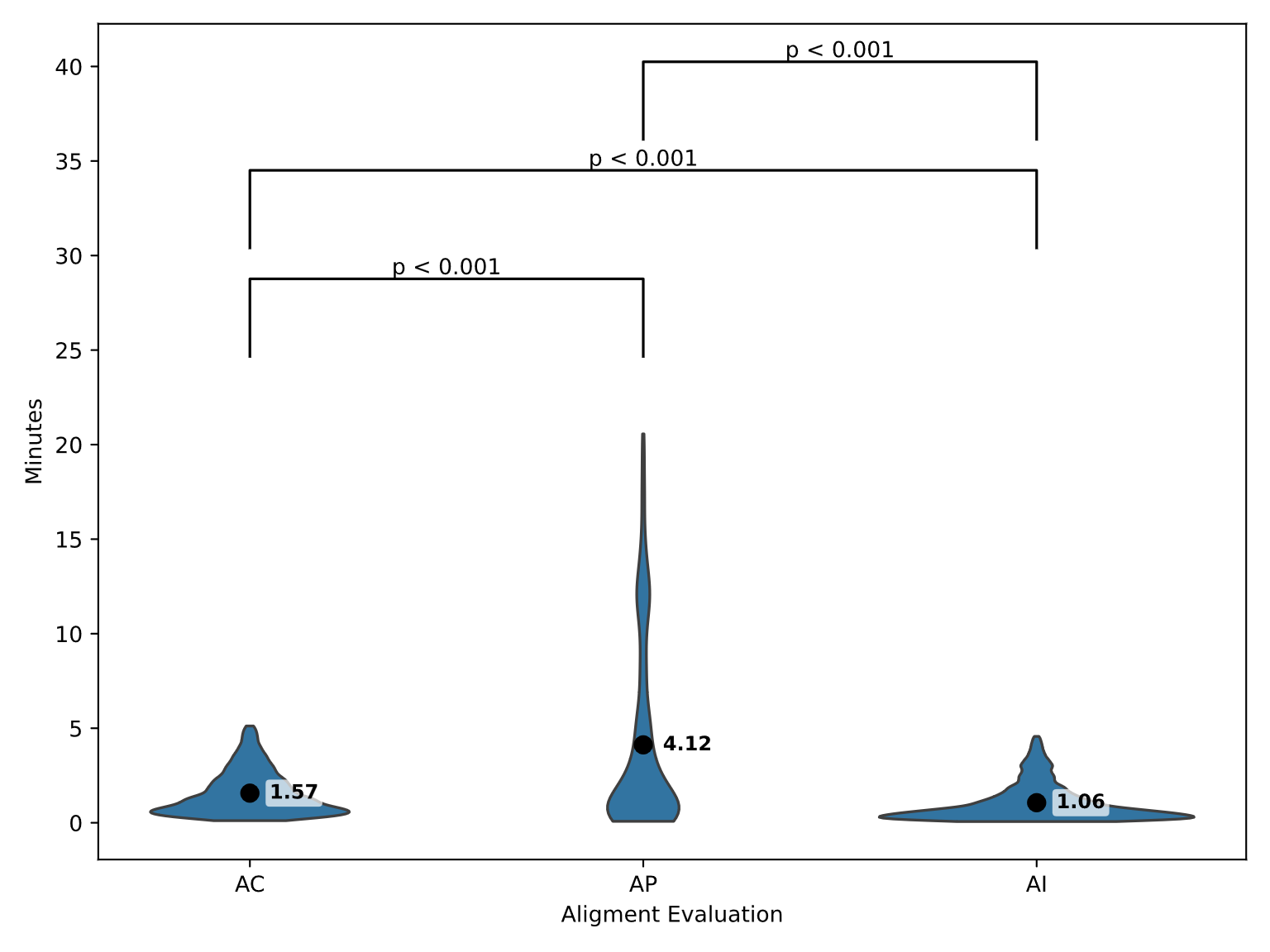

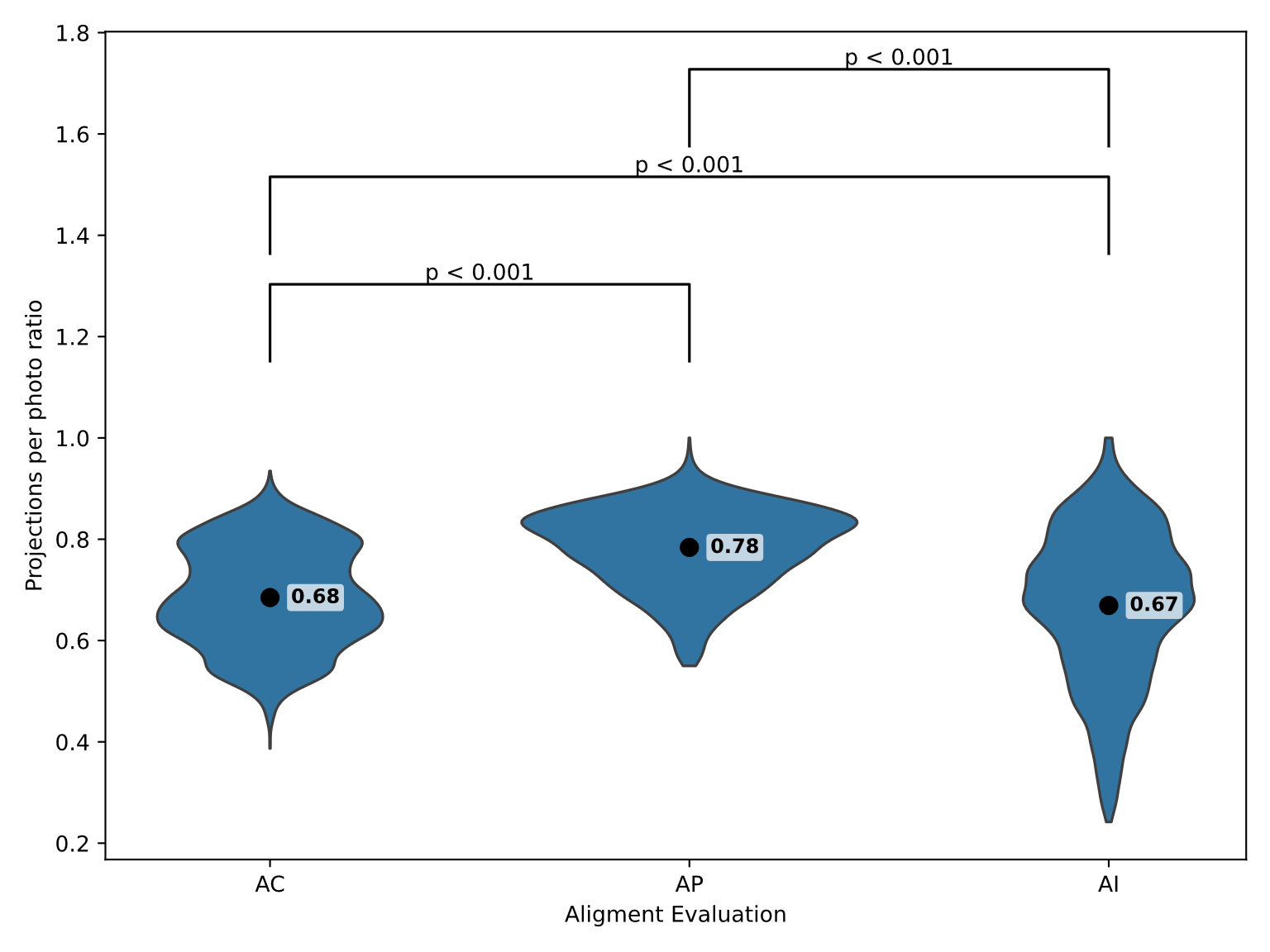


Fig. S14 SfM processing time by alignment evaluation. AP showed the longest and most variable processing times (mean = 4.12 min; median = 1.73; SD = 4.95; range 0.08–20.57), whereas AC (mean = 1.57 min; median = 1.24; SD = 1.16; range 0.11–5.12) and AI (mean = 1.06 min; median = 0.70; SD = 0.98; range 0.06–4.57) were shorter and more consistent. Differences among all groups were statistically significant (p < 0.001).

Fig. S13 Median projections ratio per photo by alignment evaluation. AP showed the highest and most consistent ratios (mean = 0.78; median = 0.80; SD = 0.08; range 0.55–1.0), followed by AC (mean = 0.68; median = 0.68; SD = 0.10; range 0.39–0.93), while AI exhibited similar central values but greater variability (mean = 0.67; median = 0.69; SD = 0.15; range 0.24–1.0). Differences among all groups were statistically significant (p < 0.001).


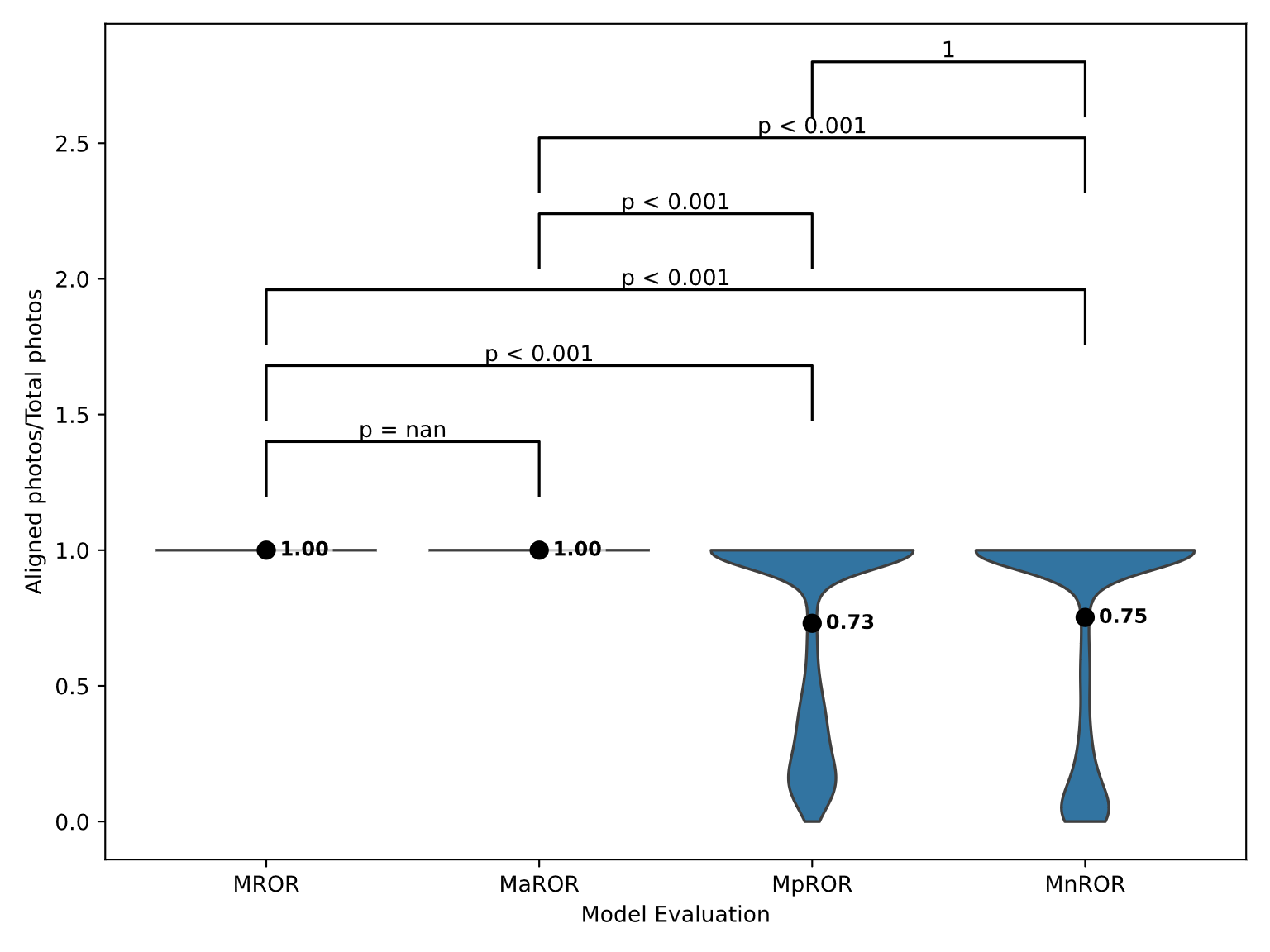

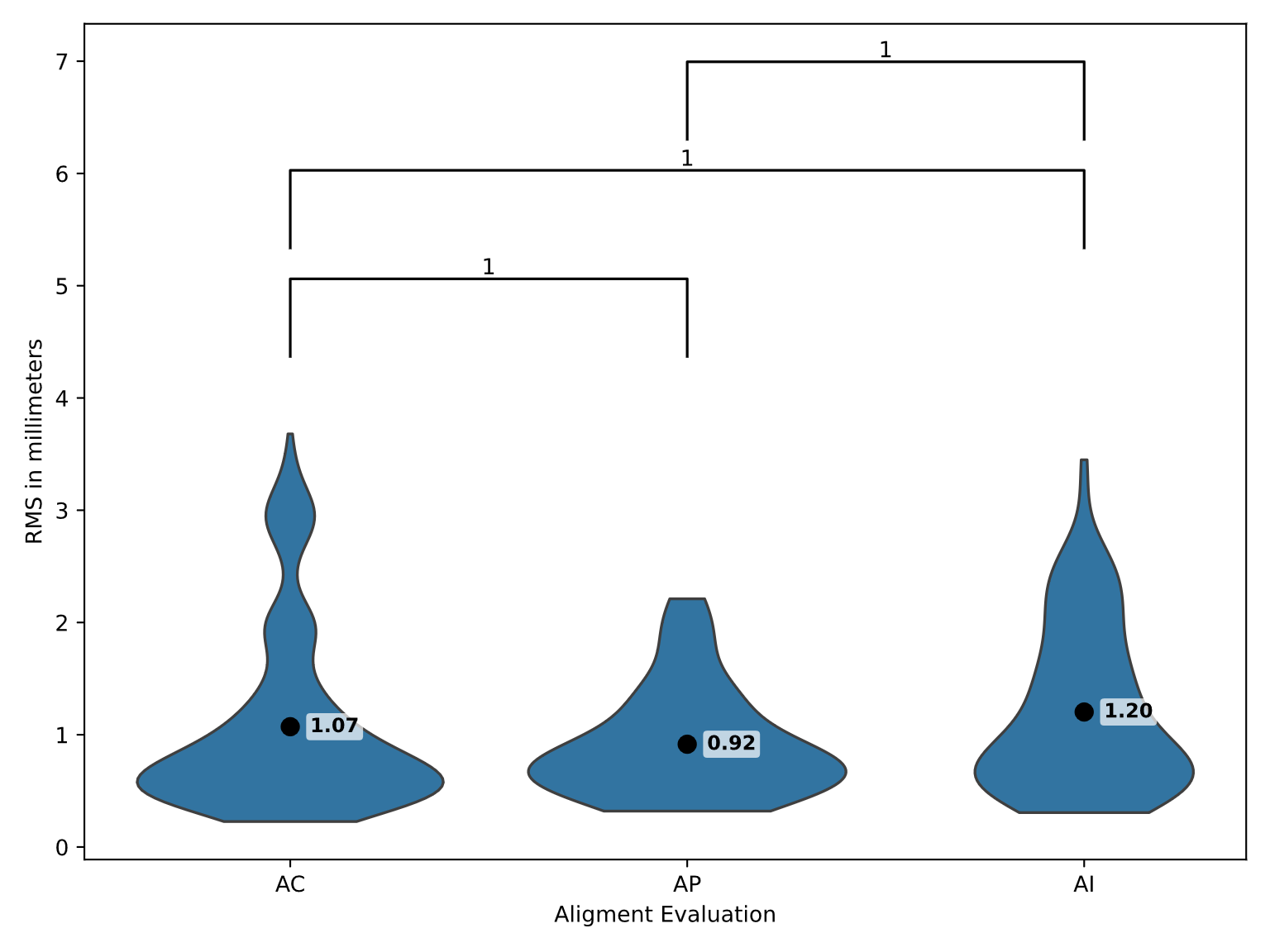


Fig. S16 Photo alignment ratio by final model evaluation. MROR and MaROR showed perfect alignment (mean = median = min = max = 1; SD = 0), preventing statistical comparison between them due to identical distributions. In contrast, MpROR (mean = 0.73; median = 1; SD = 0.36; range 0–1) and MnROR (mean = 0.75; median = 1; SD = 0.38; range 0–1) exhibited lower and more variable ratios. Both MROR and MaROR differed significantly from MpROR and MnROR (p < 0.001), while no differences were observed between MpROR and MnROR (p = 1).

Fig. S15 RMS vertex distance to reference model by alignment evaluation. AC (mean = 1.07 mm; median = 0.74; SD = 0.81; range 0.23–3.68), AP (mean = 0.92 mm; median = 0.76; SD = 0.48; range 0.32–2.21), and AI (mean = 1.20 mm; median = 0.93; SD = 0.72; range = 0.31–3.45) showed comparable distributions, with no statistically significant differences between groups (all p = 1).


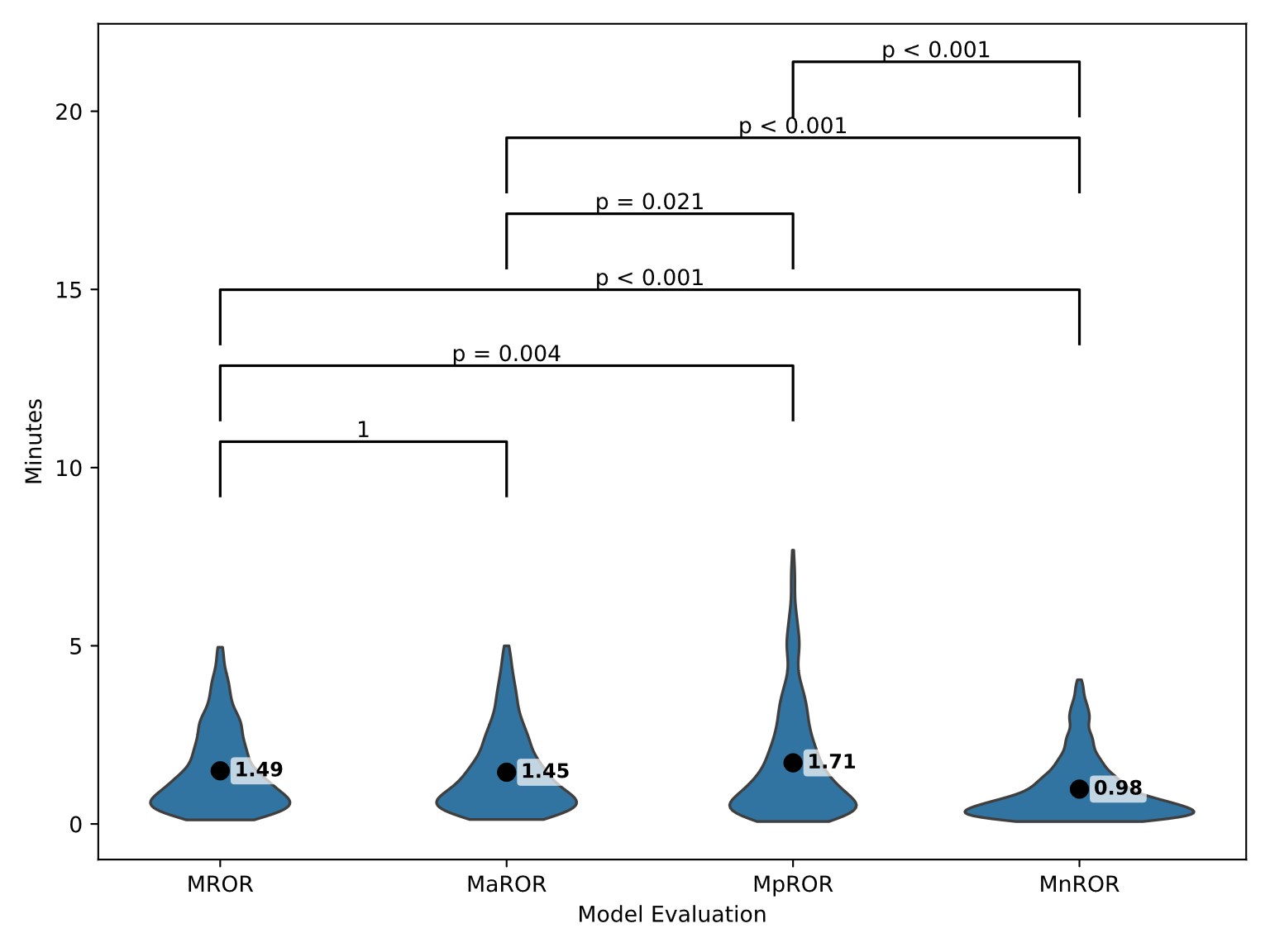

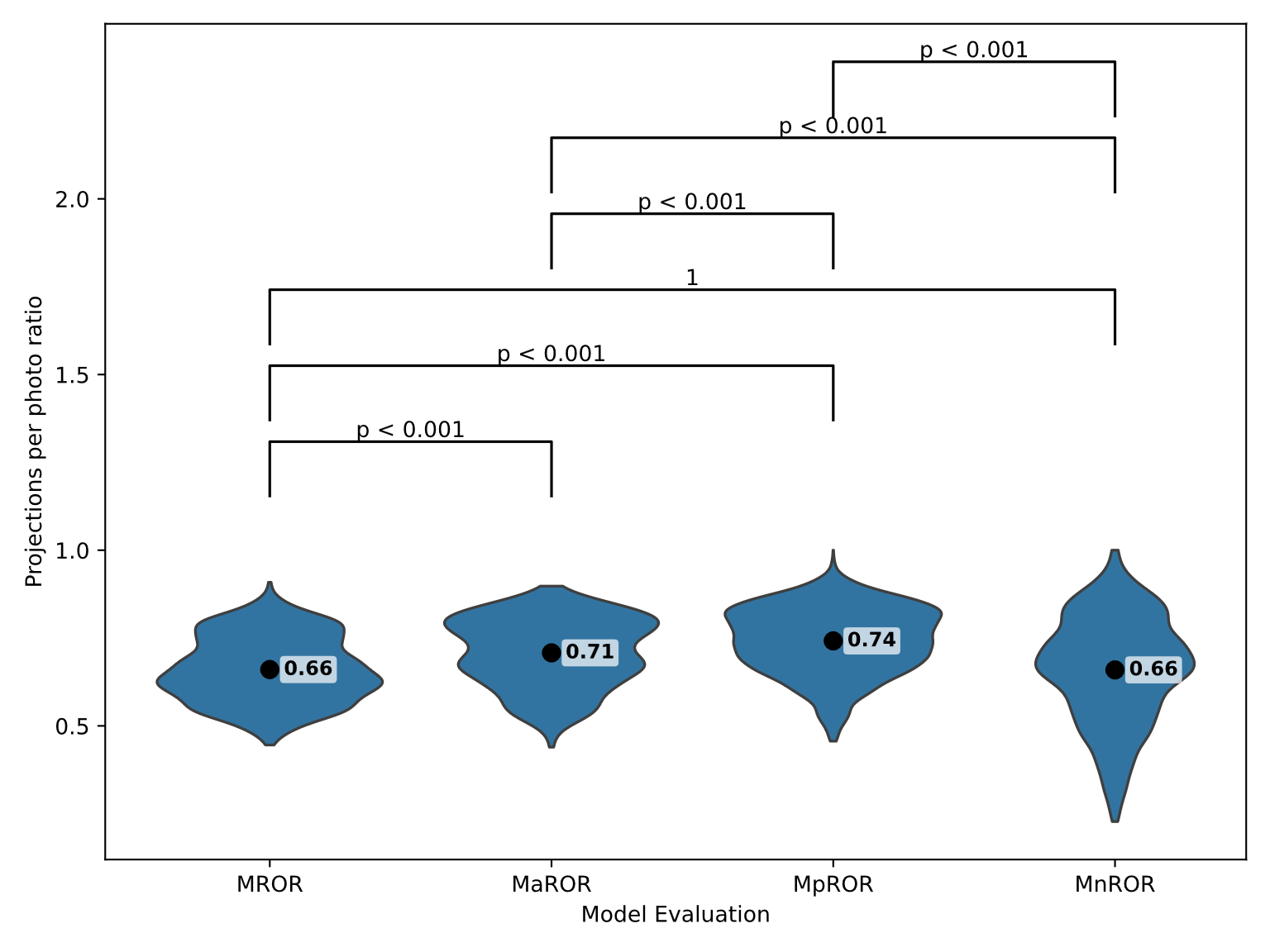

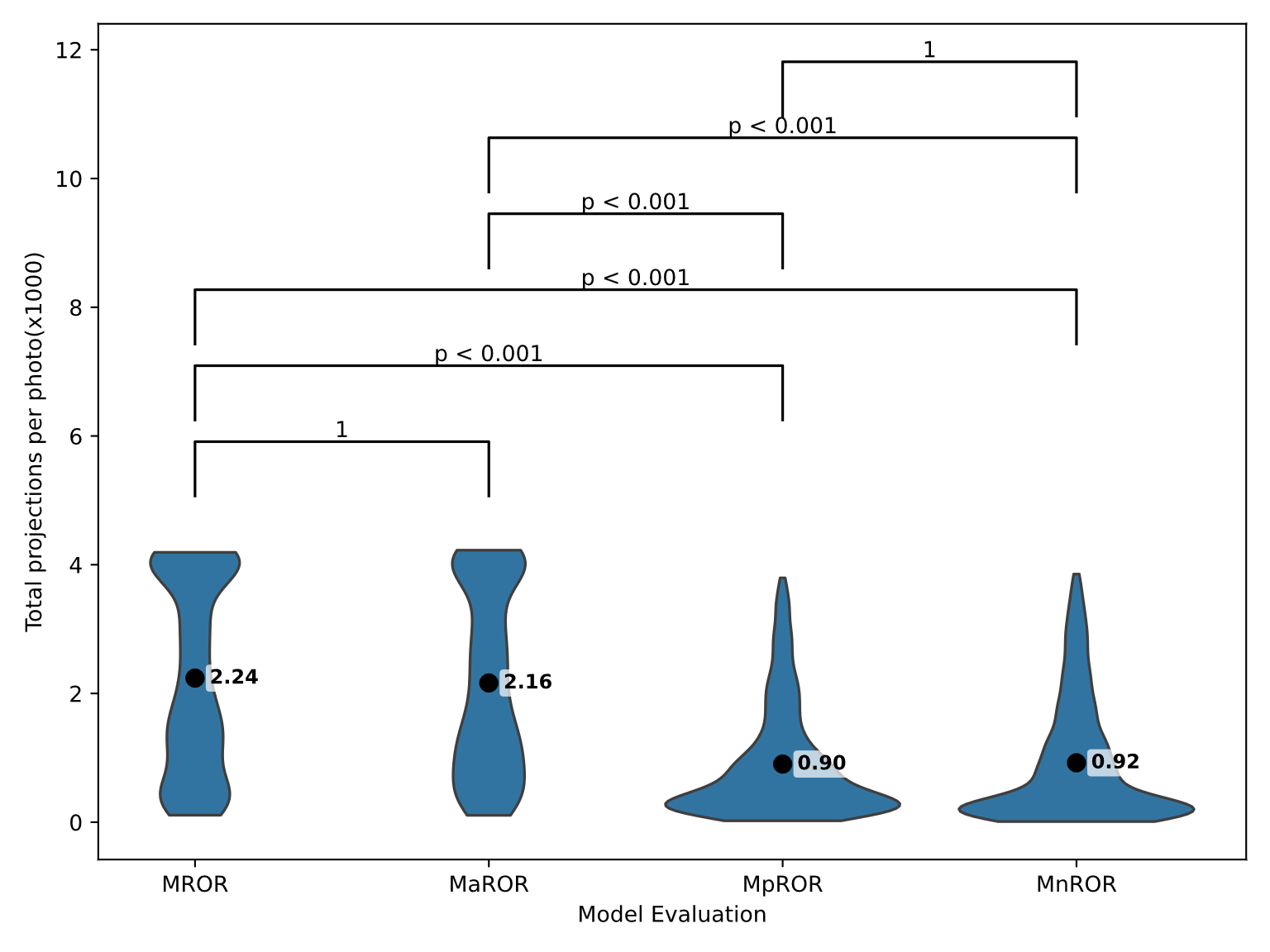

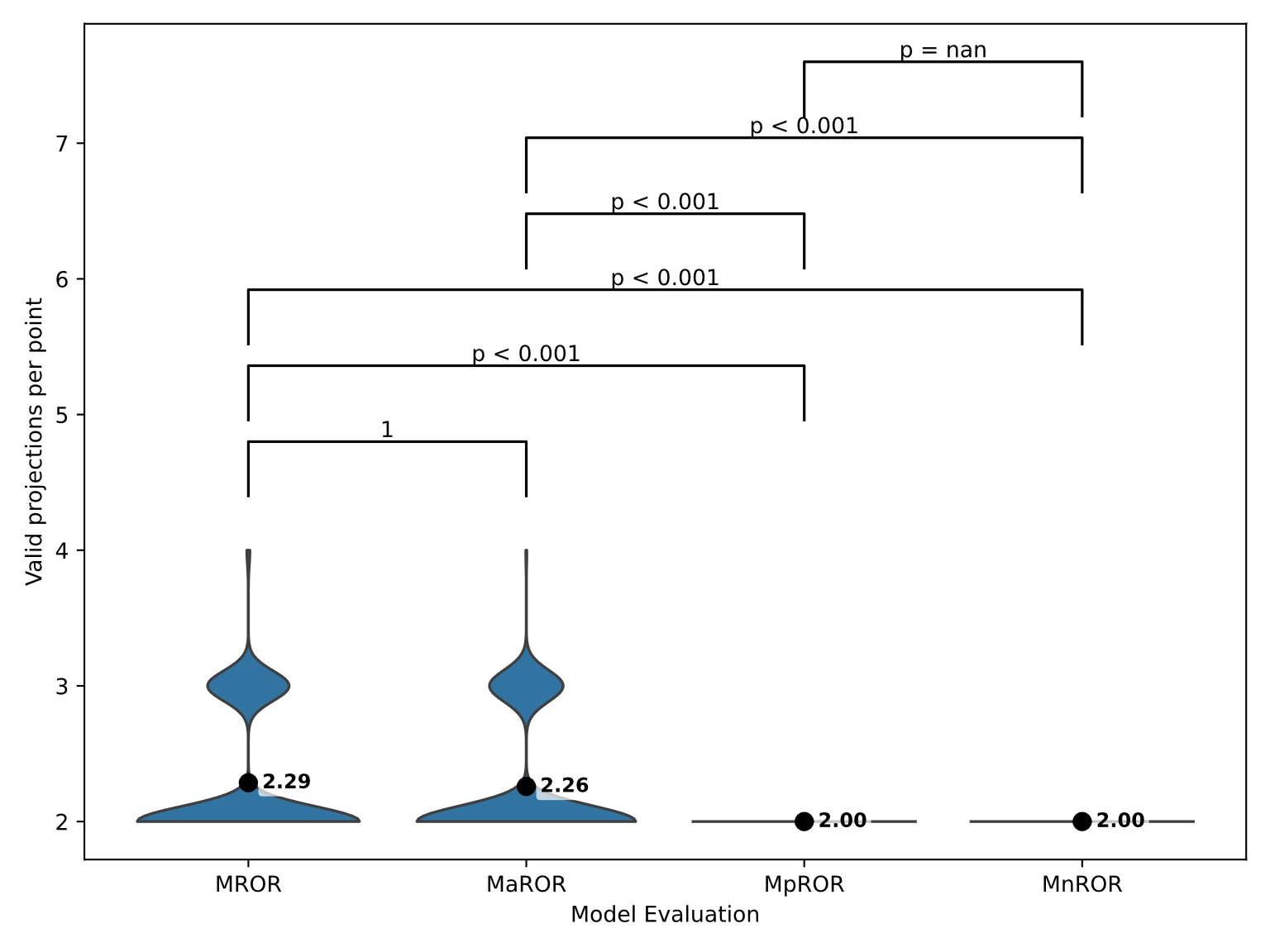


Fig. S20 SfM processing time by final model evaluation. MROR and MaROR showed similar processing times (means = 1.49 and 1.45 min; medians = 1.18 and 1.11; SD = 1.12 and 1.11; range = 0.11–5.0), with no significant differences between them (p = 1). MpROR required slightly longer and more variable processing (mean = 1.71 min; SD = 1.56; range = 0.07–7.68), while MnROR showed the shortest times (mean = 0.98 min; median = 0.67; SD = 0.87; range = 0.07–4.04). MROR and MaROR differed significantly from MpROR and MnROR (p ≤ 0.004), and all remaining pairwise comparisons were statistically significant (p < 0.05).

Fig. S19 Median projections ratio per photo by final model evaluation. MROR showed lower ratios (mean = 0.66; median = 0.65; SD = 0.09; range = 0.45–0.91), while MaROR (mean = 0.71; median = 0.72; SD = 0.10) and MpROR (mean = 0.74; median = 0.75; SD = 0.10; range 0.46–1.0) exhibited progressively higher values. MnROR showed similar central values to MROR (mean = 0.66; median = 0.67) but greater variability (SD = 0.15; range 0.23–1.0). MROR did not differ from MnROR (p = 1) but differed significantly from MaROR and MpROR (p < 0.001). All remaining pairwise comparisons were statistically significant (p < 0.001).

Fig. S18 Median of total projections per photo by final model evaluation. MROR and MaROR showed similarly high values (means = 2,238 and 2,163; medians = 1,992 and 1,914; SD = 1,477 and 1,415; range 107–4,226), with no significant differences between them (p = 1). In contrast, MpROR and MnROR had substantially lower values (means = 903 and 923; medians lower; SD = 864 and 942; range 10–3,854) and differed significantly from MROR and MaROR (p < 0.001), while no differences were observed between MpROR and MnROR (p = 1).

Fig. S17 Median number of valid projections per matched feature by final model evaluation. MROR and MaROR showed similar values (means = 2.29 and 2.26; medians = 2; SD = 0.47 and 0.45; range 2–4), with no significant differences between them (p = 1). In contrast, MpROR and MnROR were constant (mean = median = 2; SD = 0; range = 2–2) and differed significantly from MROR and MaROR (p < 0.001). No statistical comparison could be computed between MpROR and MnROR due to identical distributions.


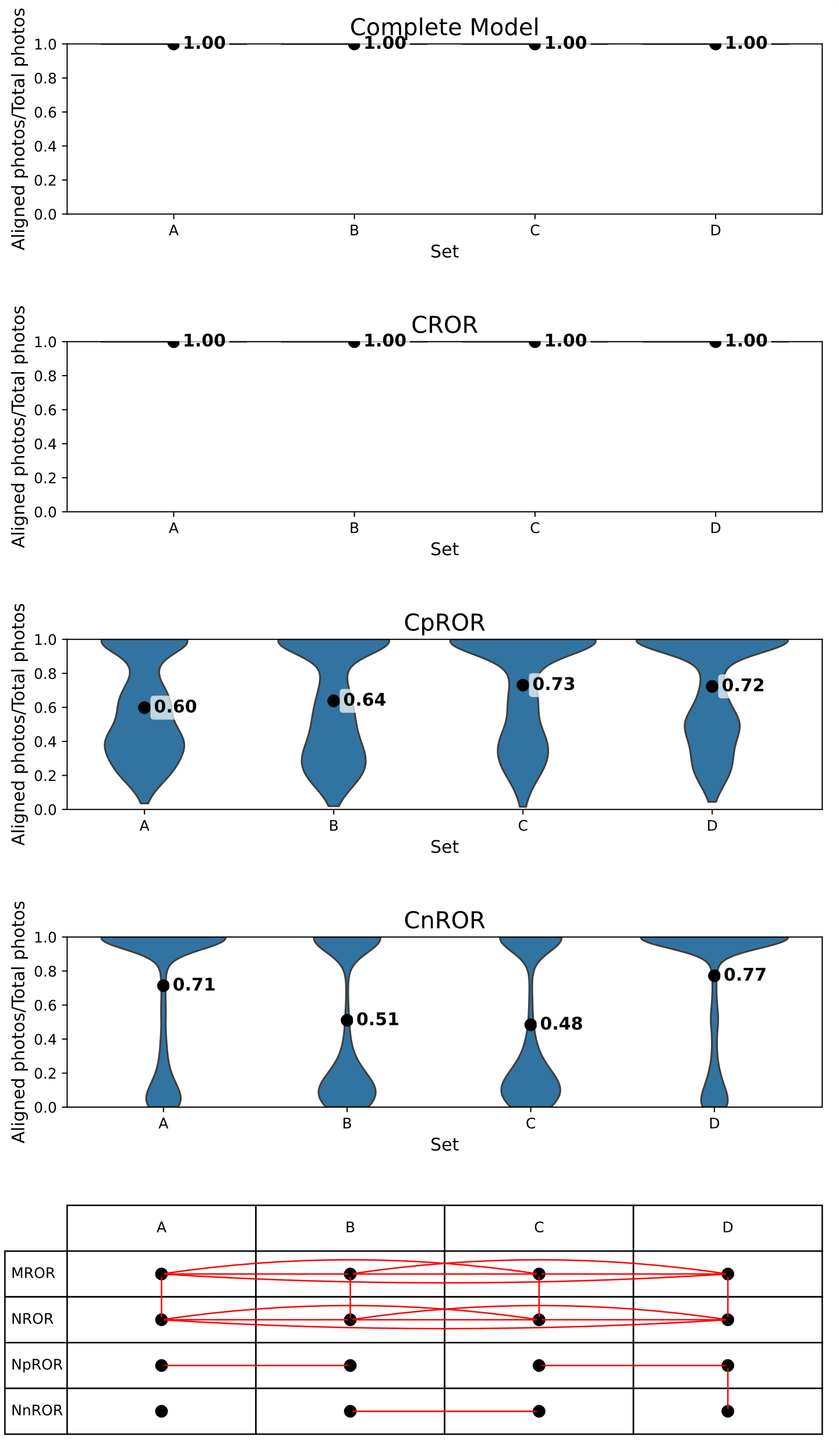

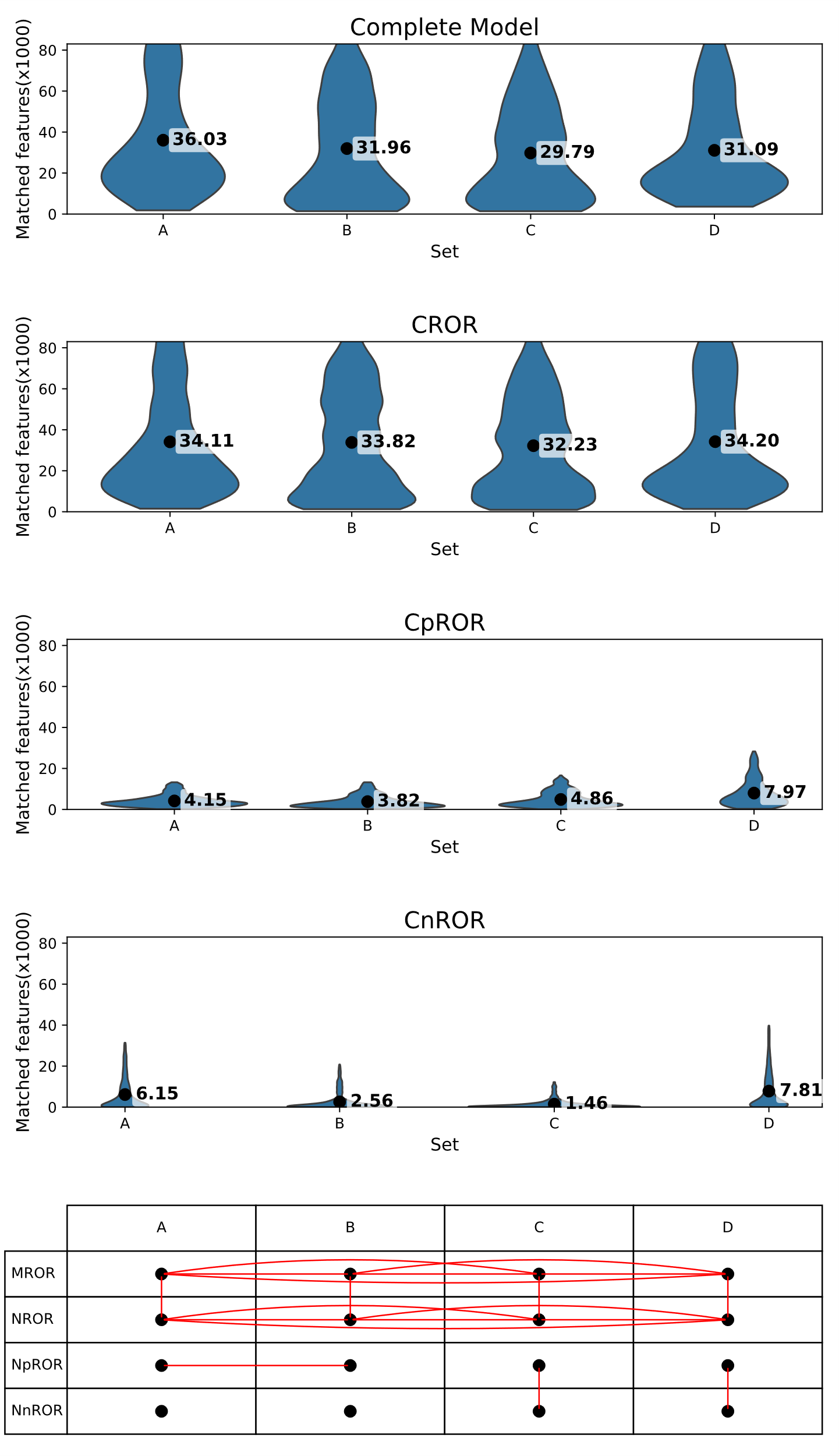


Fig. S22. Photo alignment ratio by photo capture angle. MROR and CROR showed perfect alignment across all angles (mean = 1.0; SD = 0), with no significant differences between groups or angles. In contrast, CpROR and CnROR exhibited bimodal distributions at all angles. CpROR showed means of 0.60–0.73, with peaks around 0.4 and 1.0, while CnROR showed means of 0.48–0.77, with peaks around 0.1 and 1.0. Red lines in the matrix indicate non-significant comparisons (p > 0.05).

Fig. S21. Number of matched features by photo capture angle. MROR and CROR showed comparable matched feature counts across all capture angles, with no significant differences between reconstruction types or angles (means = 29,790–36,029; SD = 22,832–26,766; range = 972–121,617). In contrast, CpROR and CnROR had significantly fewer matched features (p < 0.05), retaining around 5–22% of MROR/CROR values, with slightly higher counts at A and D angles. Red lines in the matrix indicate non-significant comparisons (p > 0.05).


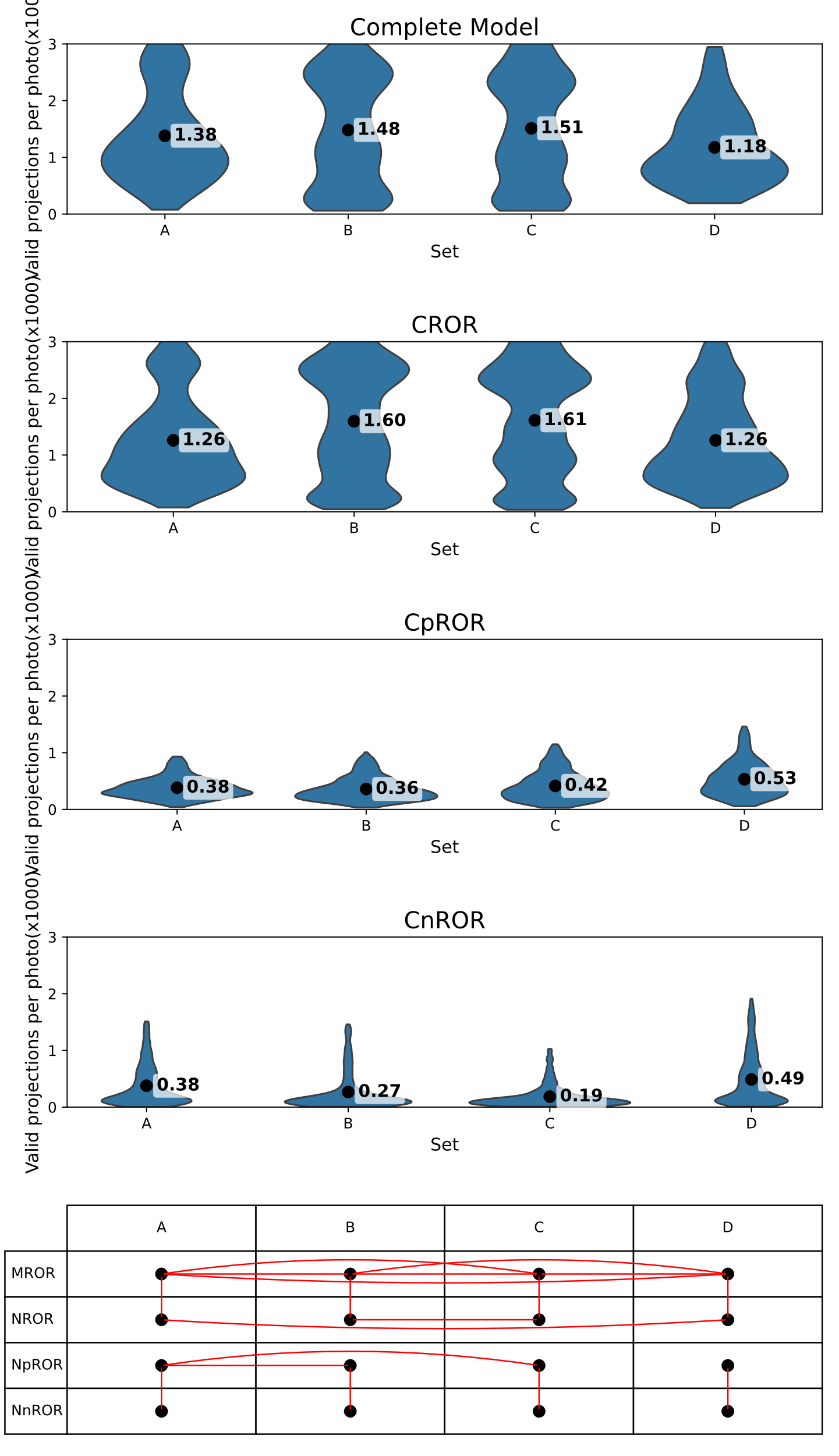

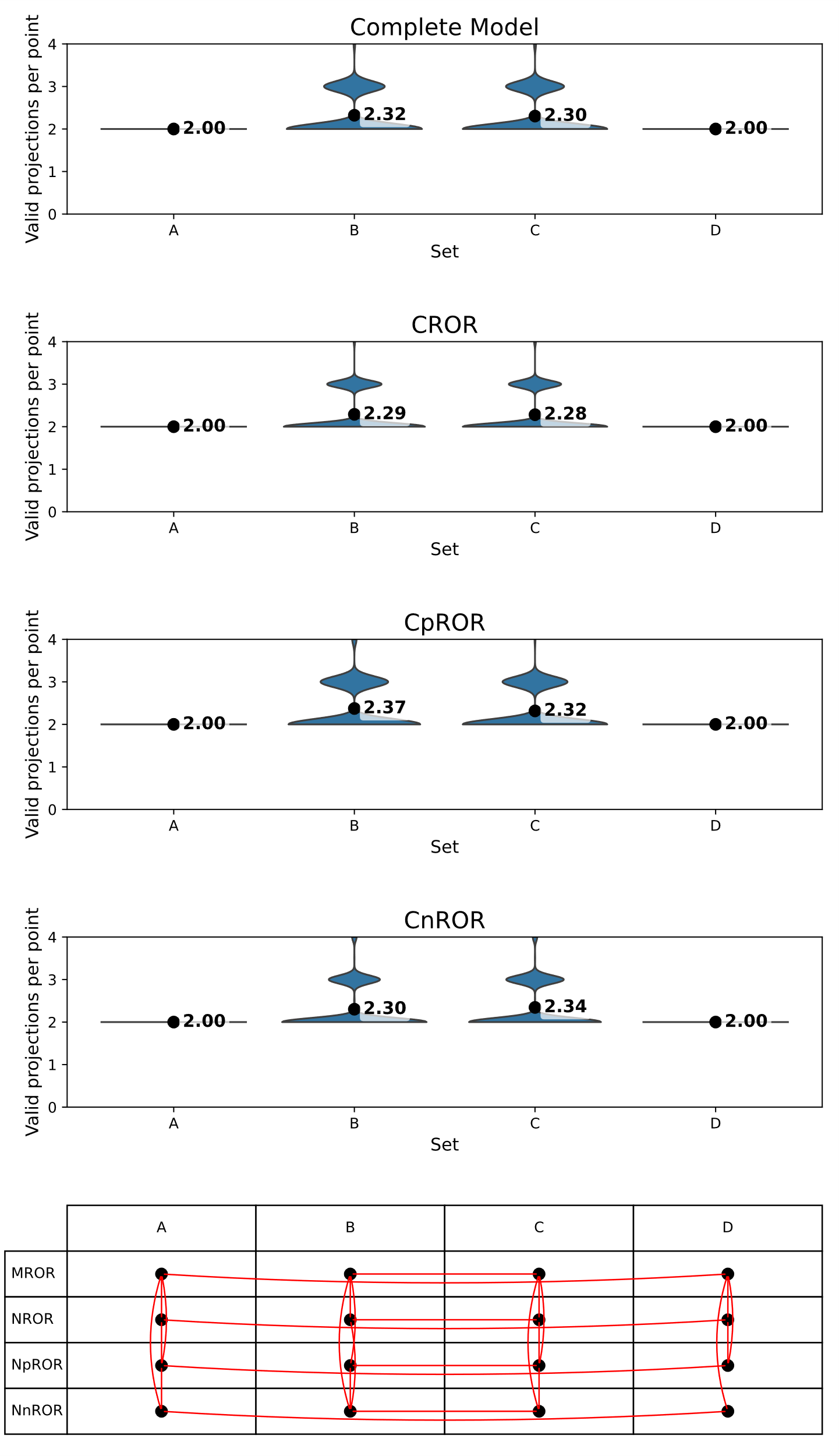


Fig. S23. Number of valid projections per matched feature by photo capture angle. Angles A and D showed identical values (mean = 2; SD = 0), while angles B and C showed slightly higher and more variable values (means = 2.28–2.37; SD = 0.48). No significant differences were observed between A vs. D or B vs. C across groups. Red lines in the matrix indicate non-significant comparisons (p > 0.05).

Fig. S24. Median valid projections per photo by photo capture angle. In MROR and CROR, no significant differences were observed between A vs. D or B vs. C. Angles A and D showed lower values (means = 1,175–1,380; SD = 667–803), while B and C were slightly higher (means = 1,482–1,612; SD = 954–982). No differences were found between MROR and CROR across angles. CpROR and CnROR showed significantly lower values, retaining around 16–32% of MROR/CROR projections. Red lines in the matrix indicate non-significant comparisons (p > 0.05).


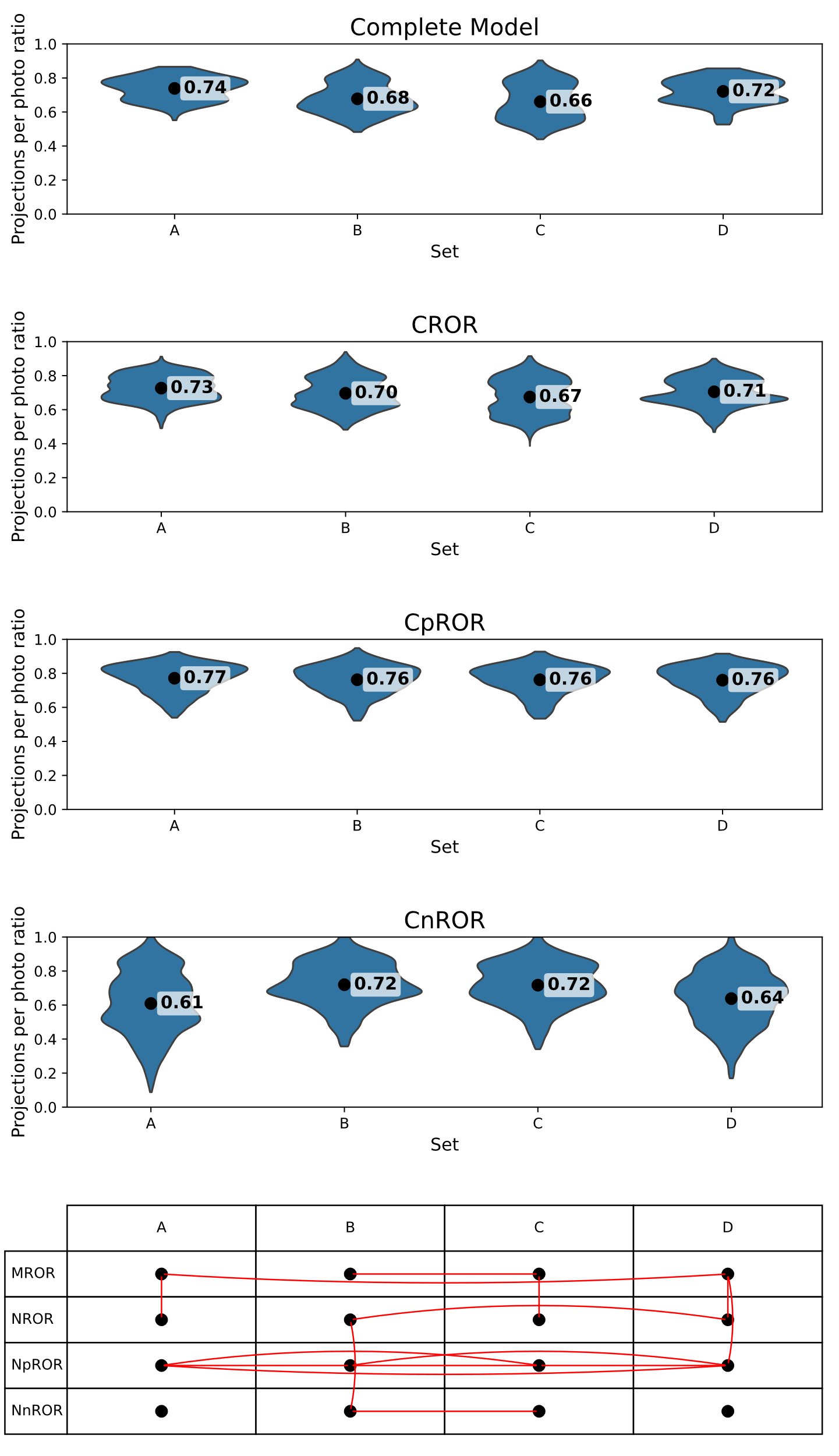

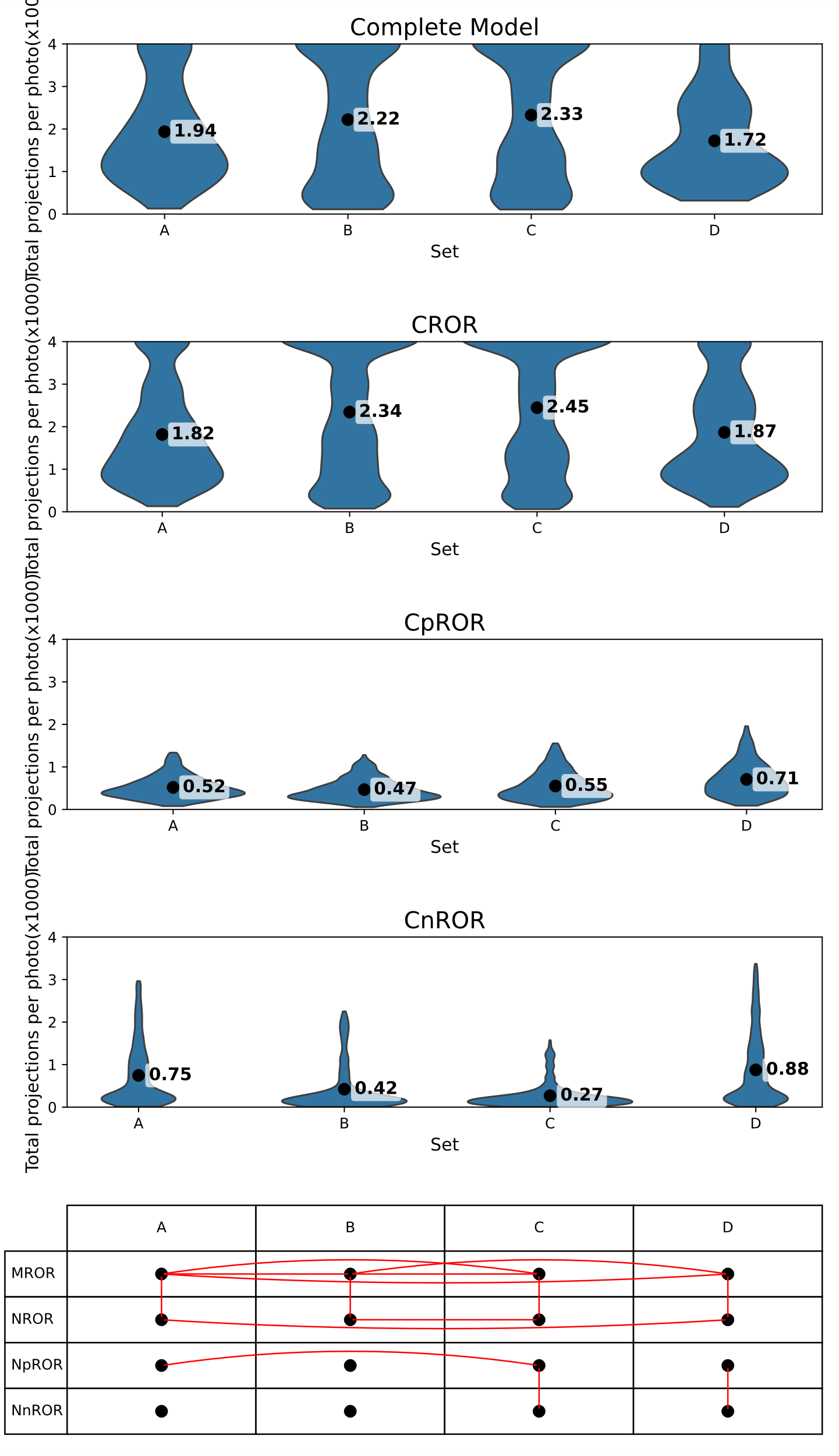


Fig. S25 Median total projections per photo by photo capture angle. In MROR and CROR, no significant differences were observed between A vs. D or B vs. C. Angles A and D showed lower values (means = 1,721–1,936; SD = 1,046–1,169), while B and C were slightly higher (means = 2,219–2,447; SD = 1,463–1,501). No differences were found between MROR and CROR across angles. CpROR and CnROR showed significantly lower values, retaining around 14–35% of MROR/CROR projections. Red lines in the bottom matrix shows no significant correlations (p > 0.05).

Fig. S26. Median projections ratio per photo by photo capture angle. In MROR, no significant differences were observed between A vs. D (means = 0.72–0.74; SD = 0.07; range 0.53–0.86) or B vs. C (means = 0.66–0.68; SD = 0.09–0.11; range = 0.44–0.91). CROR showed a similar pattern, with no differences across angles relative to MROR, except for a minor difference at B. CpROR exhibited consistent ratios across angles (means = 0.76–0.77), while CnROR showed the widest variability (range = 0.09–1.0). Red lines in the matrix indicate non-significant comparisons (p > 0.05).


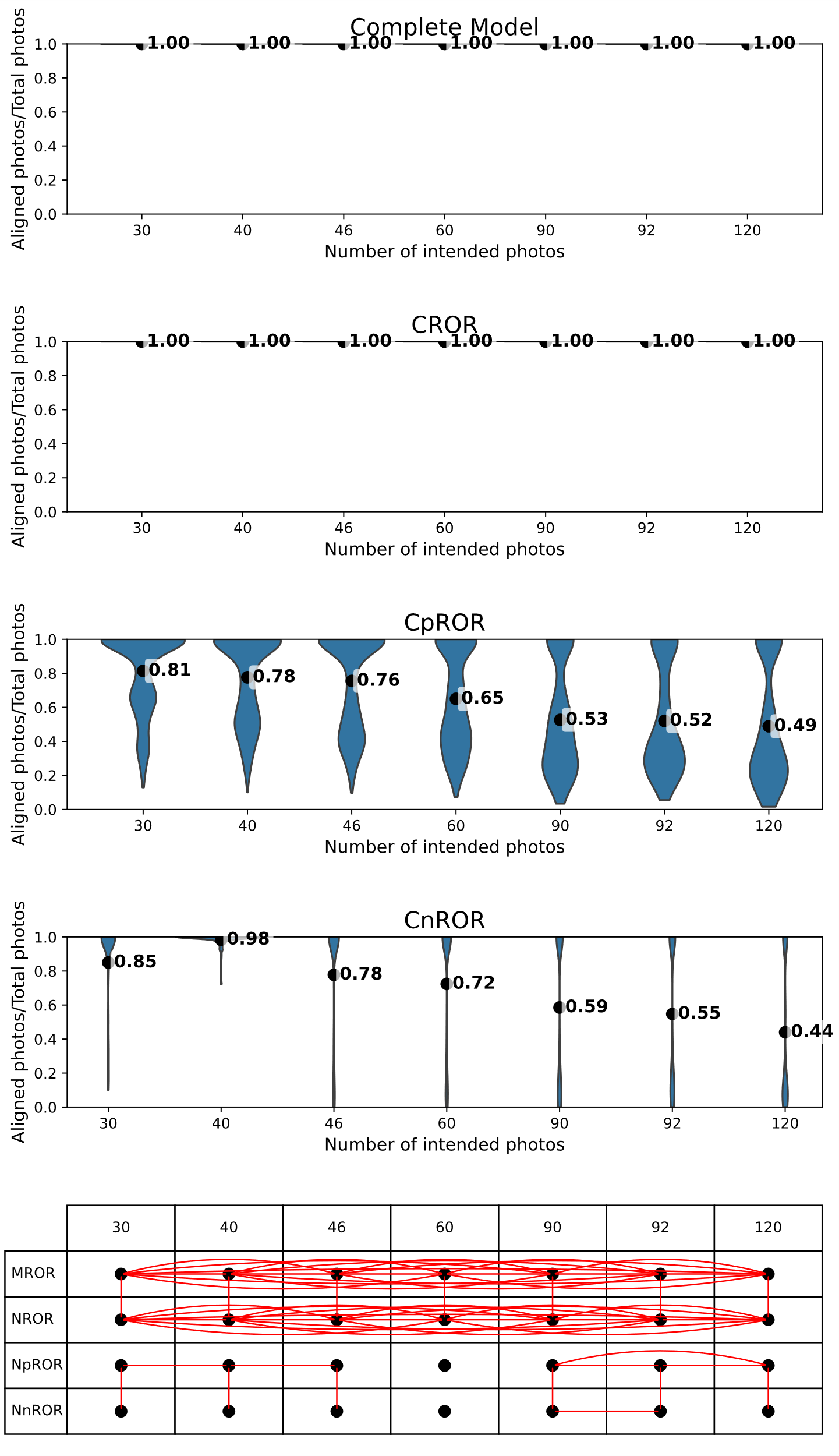

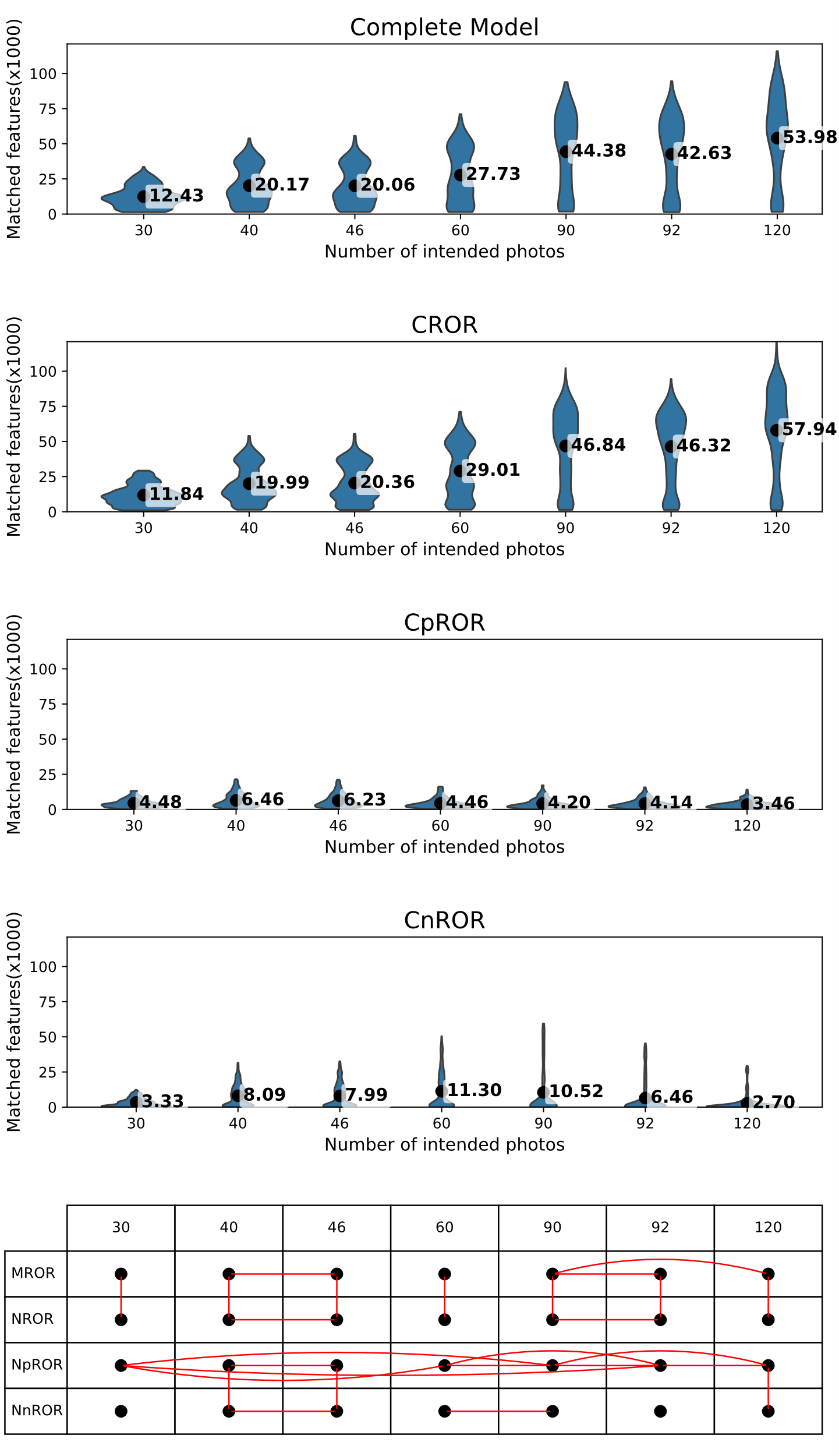


Fig. S28 Photo alignment ratio by number of intended photos. In both the complete model (MROR) and CROR, the alignment ratio remained constant at 1.00 across all photo groups (30–120), with no observed variability (SD = 0; min = max = 1). Due to the absence of variation, statistical comparisons within MROR and CROR could not be computed. Likewise, no statistically significant differences were observed between MROR and CROR (p = 1). Red lines in the bottom matrix shows no significant correlations (p > 0.05).

Fig. S27 Number of matched features by number of intended photos. In the complete model (MROR), mean matched features increased from 12.43 (30 photos) to 53.98 (120 photos), with intermediate values of 20.17 (40), 20.06 (46), 27.73 (60), 44.38 (90), and 42.63 (92). In CROR, a similar increasing trend was observed, ranging from 11.84 (30 photos) to 57.94 (120 photos), with intermediate values of 19.99 (40), 20.36 (46), 29.01 (60), 46.84 (90), and 46.32 (92). Most within-method comparisons were statistically significant (p < 0.001), except between 40–46 and 90–92 in both methods, and between 90–120 in MROR (p = 0.078). No statistically significant differences were found between MROR and CROR across classifications. Red lines in the bottom matrix shows no significant correlations (p > 0.05).


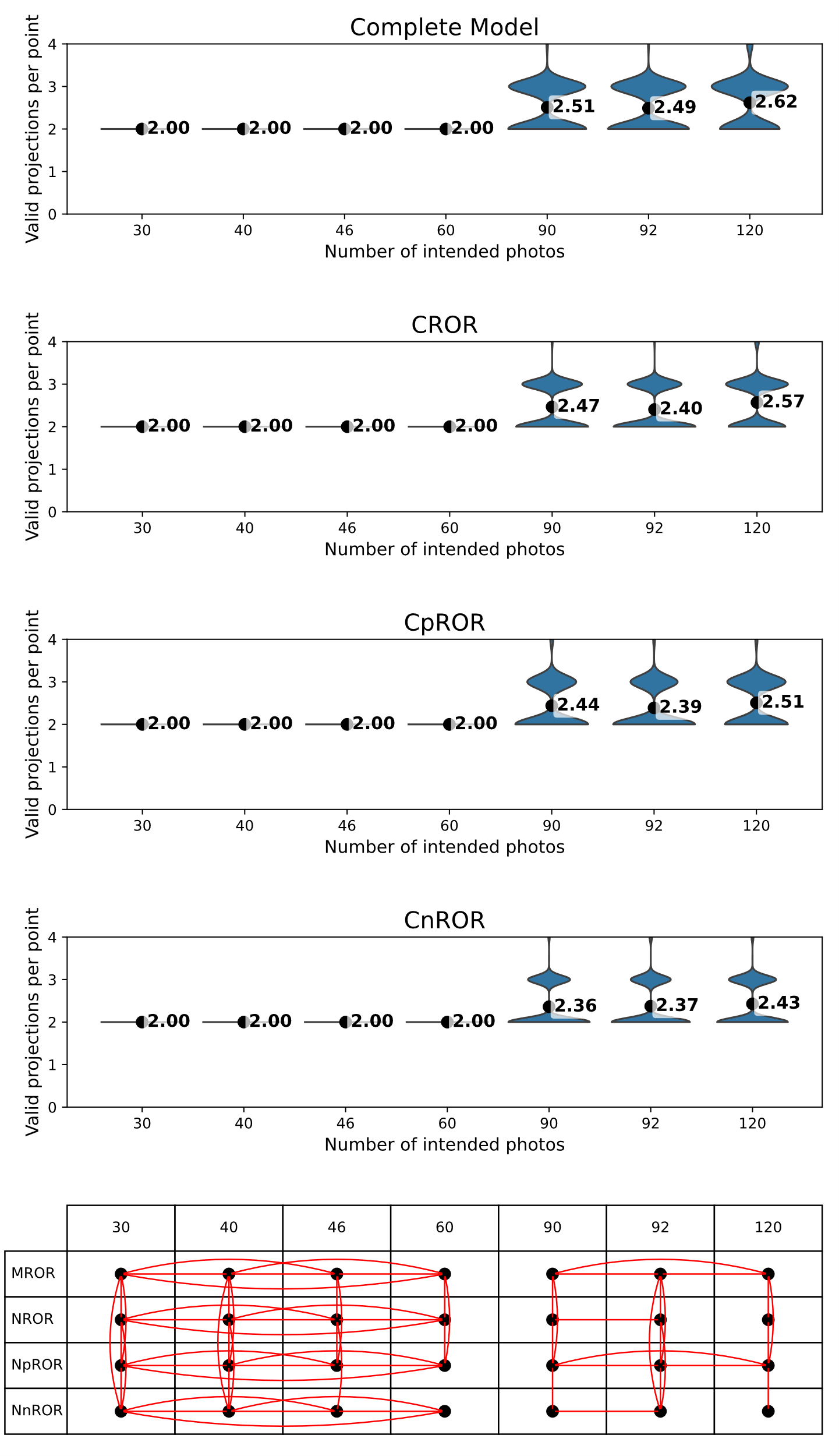

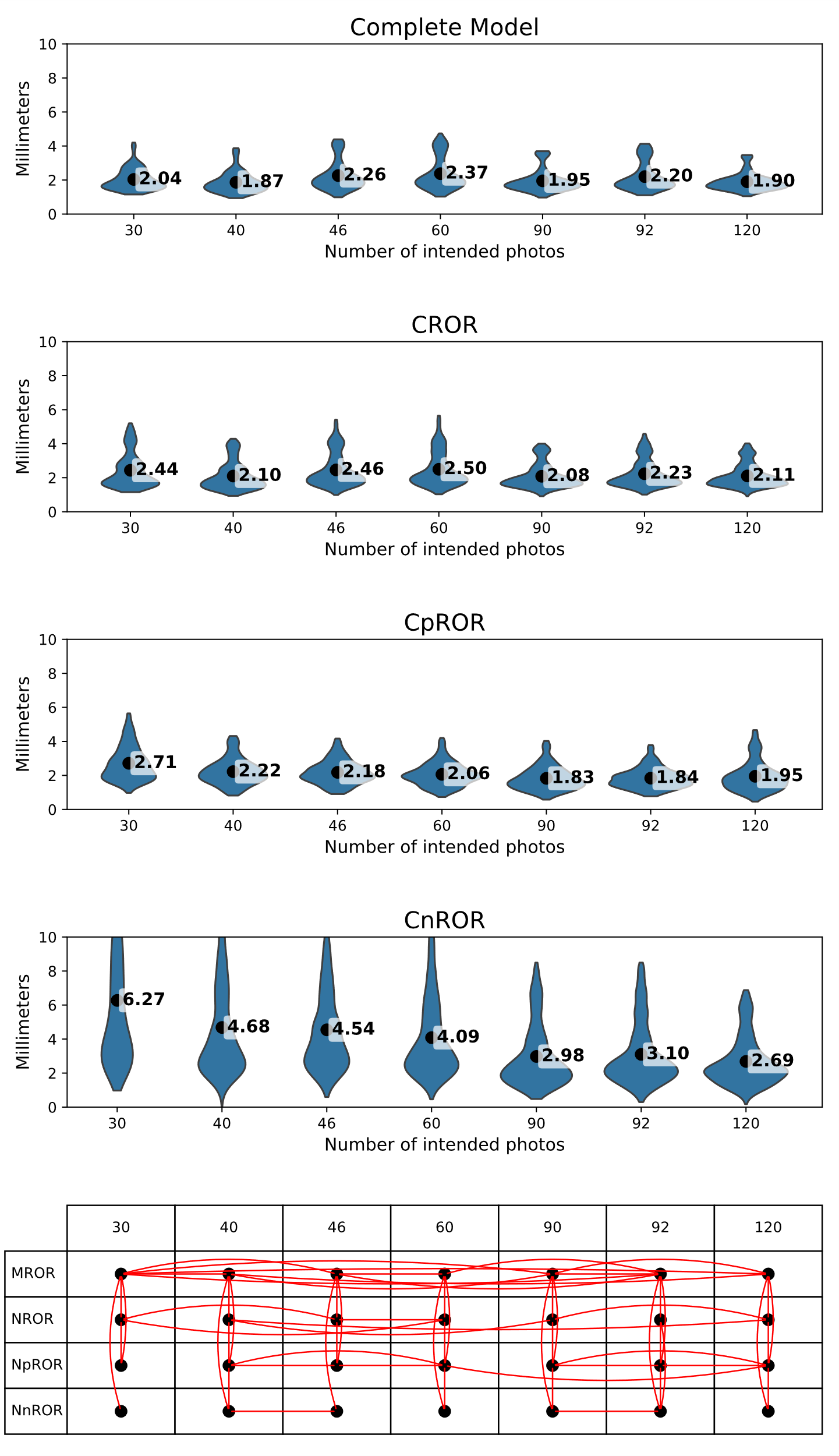


Fig. S30 Median of valid projections per feature point by number of intended photos. In the complete model (MROR), the mean number of valid projections remained constant at 2.00 for lower photo counts (30–60), with no variability (SD = 0), and increased for higher photo counts to 2.51 (90), 2.49 (92), and 2.62 (120). A similar pattern was observed in CROR, where values remained at 2.00 for 30–60 photos and increased to 2.47 (90), 2.40 (92), and 2.57 (120). Within both MROR and CROR, no statistical comparisons could be computed among the lower groups (30–60) due to identical distributions. For higher photo counts, no statistically significant differences were observed between 90–92, 90–120, and 92–120 in MROR, and between 90–92 in CROR (p = 1). Overall, no statistically significant differences were found between MROR and CROR across classifications. Red lines in the bottom matrix shows no significant correlations (p > 0.05).

Fig. S29 RMS of point error per matched feature (mm) by number of intended photos. In the complete model (MROR), mean RMS error remained relatively stable across groups, ranging from 1.87 to 2.37 mm, with a slight peak at 60 photos (2.37 mm) and lower values at 40 (1.87 mm) and 120 (1.90 mm). In CROR, a similar pattern was observed, with mean values between 2.08 and 2.50 mm, reaching the highest value at 60 photos (2.50 mm). Overall, variability was moderate and comparable between methods. Most pairwise comparisons within MROR were not statistically significant, although significant differences were observed in specific comparisons (e.g., 40 vs. 46/60/92; 46 vs. 120; 60 vs. 120; 30 vs. 60). In CROR, most comparisons were statistically significant, with some exceptions (e.g., 30 vs. 46/60; 40 vs. 90/120; 46 vs. 60; 90 vs. 120). No statistically significant differences were found between MROR and CROR across classifications. Red lines in the bottom matrix shows no significant correlations (p > 0.05).


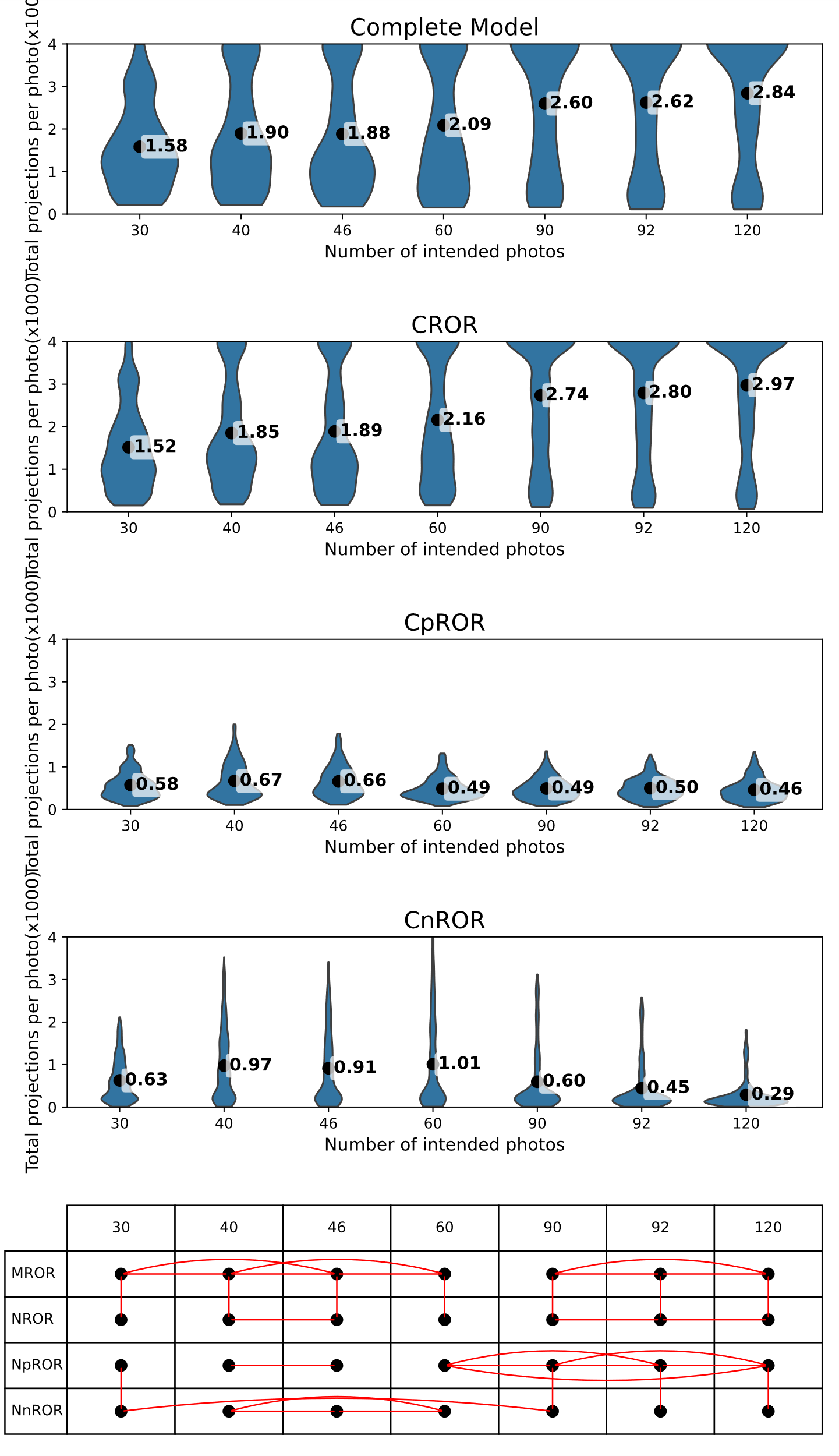

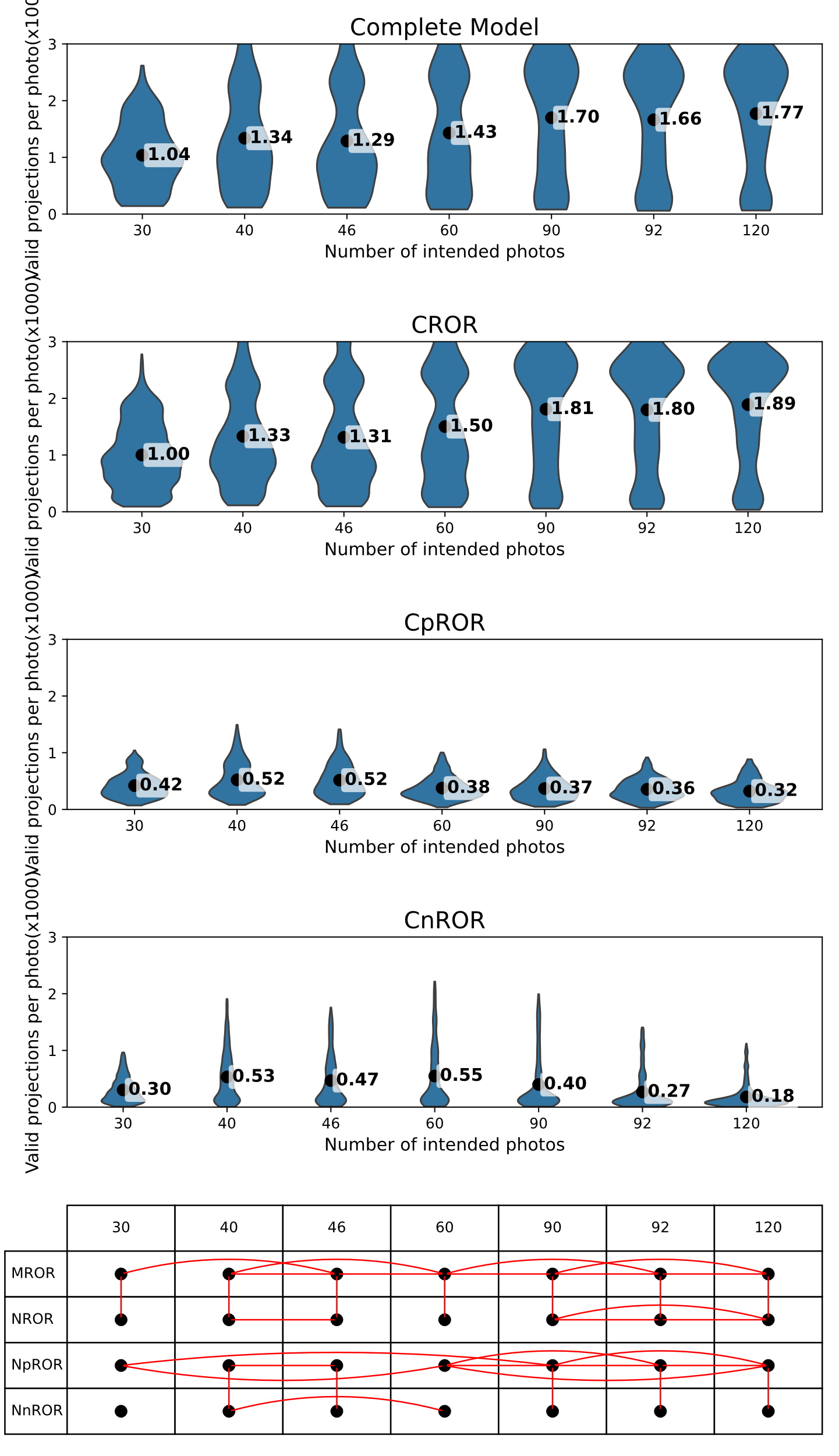


Fig. S32 Median total projections per photo by number of intended photos. In the complete model (MROR), values increased from 1.58 (30 photos) to 2.84 (120 photos), with intermediate values of 1.90 (40), 1.88 (46), 2.09 (60), 2.60 (90), and 2.62 (92). In CROR, a similar increasing trend was observed, ranging from 1.52 (30 photos) to 2.97 (120 photos), with intermediate values of 1.85 (40), 1.89 (46), 2.16 (60), 2.74 (90), and 2.80 (92). Most pairwise comparisons within MROR showed statistically significant differences (p < 0.001), except between adjacent or close groups (e.g., 30–40–46, 40–46–60, 46–60, 60–90–92, and 90–92–120). In CROR, most comparisons were also statistically significant, with exceptions between 40–46, 90–92, and 92–120. No statistically significant differences were observed between MROR and CROR across classifications. Red lines in the bottom matrix shows no significant correlations (p > 0.05).

Fig. S31 Median number of valid projections per photo by number of intended photos. In the complete model (MROR), mean values increased from 1.04 (30 photos) to 1.77 (120 photos), with intermediate values of 1.34 (40), 1.29 (46), 1.43 (60), 1.70 (90), and 1.66 (92). In CROR, a similar increasing trend was observed, ranging from 1.00 (30 photos) to 1.89 (120 photos), with intermediate values of 1.33 (40), 1.31 (46), 1.50 (60), 1.81 (90), and 1.80 (92). Overall, values increased with the number of photos, with moderate variability across groups. Most comparisons within MROR were not statistically significant, except for higher photo counts (e.g., 30 vs. 60/90/92/120; 40 vs. 120; 46 vs. 90; p < 0.001). In CROR, most comparisons were statistically significant, except between 40–46, 90–92, and 92–120. No statistically significant differences were found between MROR and CROR across classifications. Red lines in the bottom matrix shows no significant correlations (p > 0.05).


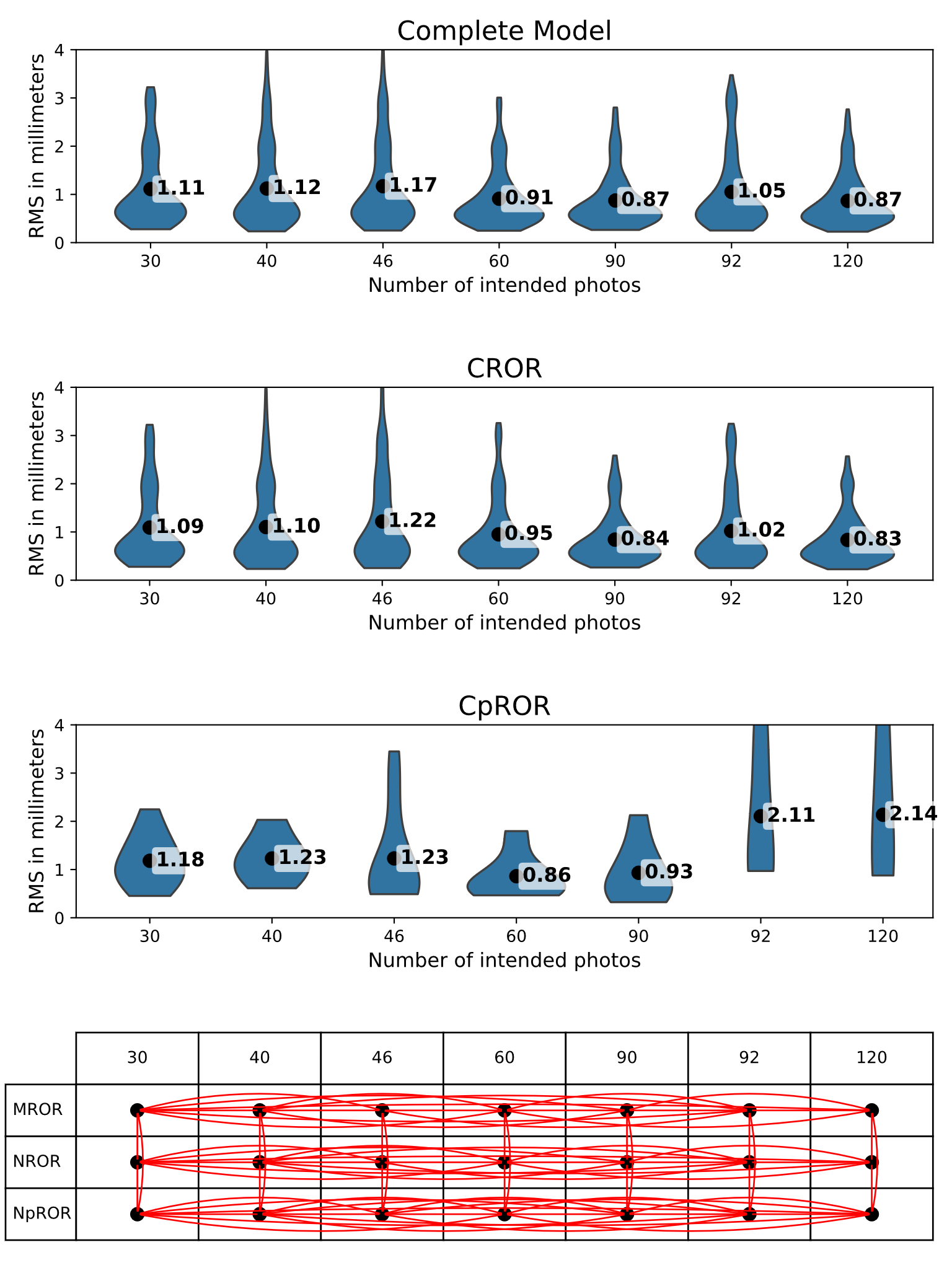

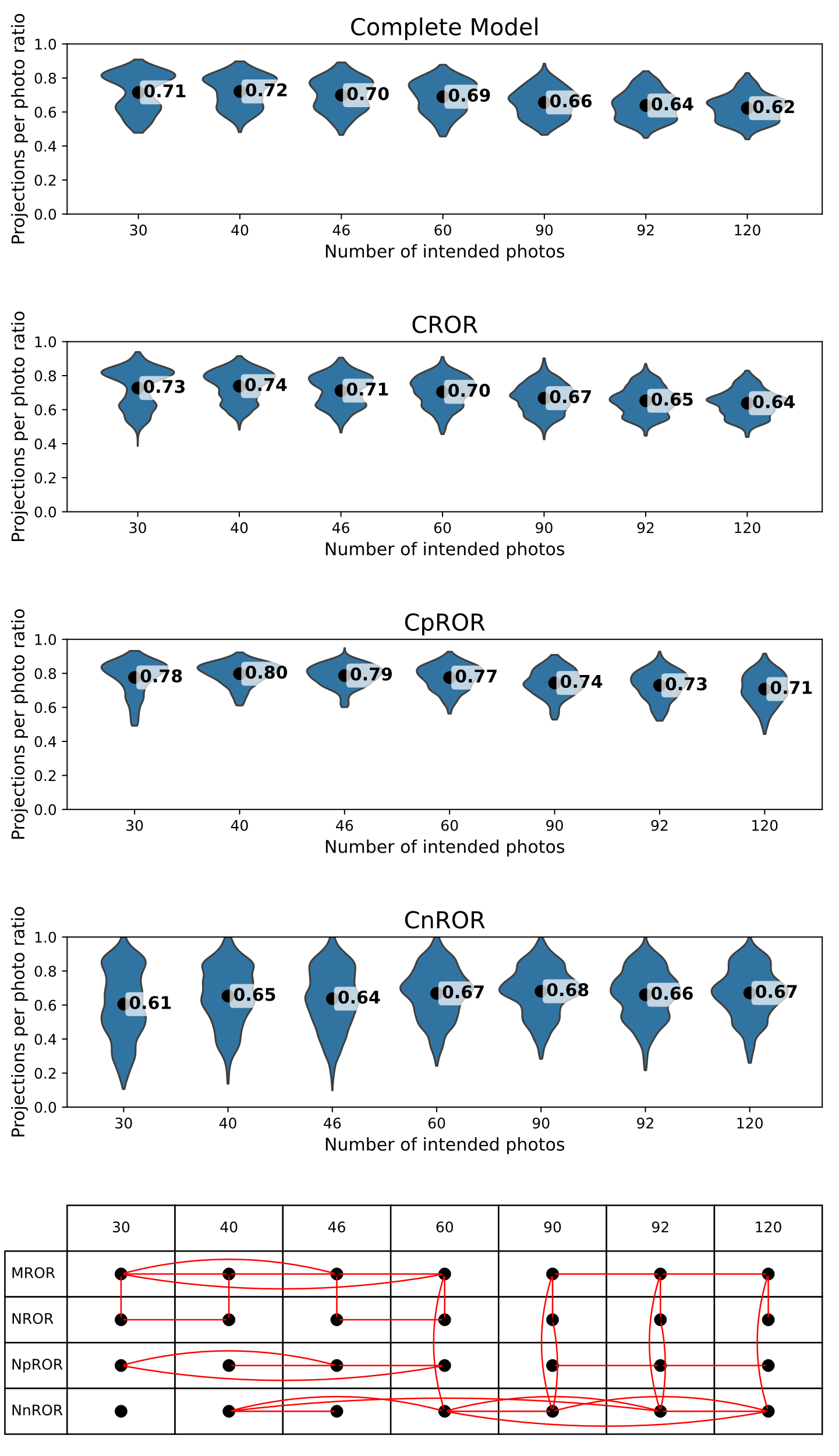


Fig. S34 RMS of vertex distance from ground truth (mm) by number of intended photos. In the complete model (MROR), mean RMS values showed moderate variability, ranging from 0.87 mm to 1.17 mm, with higher values at 46 photos (1.17 mm) and lower values at 90 and 120 photos (0.87 mm). In CROR, a similar pattern was observed, with mean values between 0.83 mm and 1.22 mm, peaking at 46 photos (1.22 mm) and decreasing at higher photo counts. Overall, variability was comparable between methods, with slightly lower values at higher photo counts. Most pairwise comparisons within MROR were not statistically significant, although some significant differences were observed (e.g., 40 vs. 120; 46 vs. 90/120). In CROR, no statistically significant differences were observed across classifications. No statistically significant differences were found between MROR and CROR across all photo groups (p = 1). Red lines in the bottom matrix shows no significant correlations (p > 0.05).

Fig. S33 Median projections per photo ratio by number of intended photos. In the complete model (MROR), mean values showed a gradual decrease as the number of photos increased, from 0.71 (30 photos) to 0.62 (120 photos), with intermediate values of 0.72 (40), 0.70 (46), 0.69 (60), 0.66 (90), and 0.64 (92). A similar decreasing trend was observed in CROR, with values declining from 0.73 (30 photos) to 0.64 (120 photos), and intermediate values of 0.74 (40), 0.71 (46), 0.70 (60), 0.67 (90), and 0.65 (92). Most pairwise comparisons within MROR were statistically significant (p < 0.001), except between closely related groups (e.g., 30–40–46–60, 46–60, 90–92, and 92–120). In CROR, most comparisons were also statistically significant, with exceptions between 30–40 and 46–60. No statistically significant differences were observed between MROR and CROR across classifications. Red lines in the bottom matrix shows no significant correlations (p > 0.05).


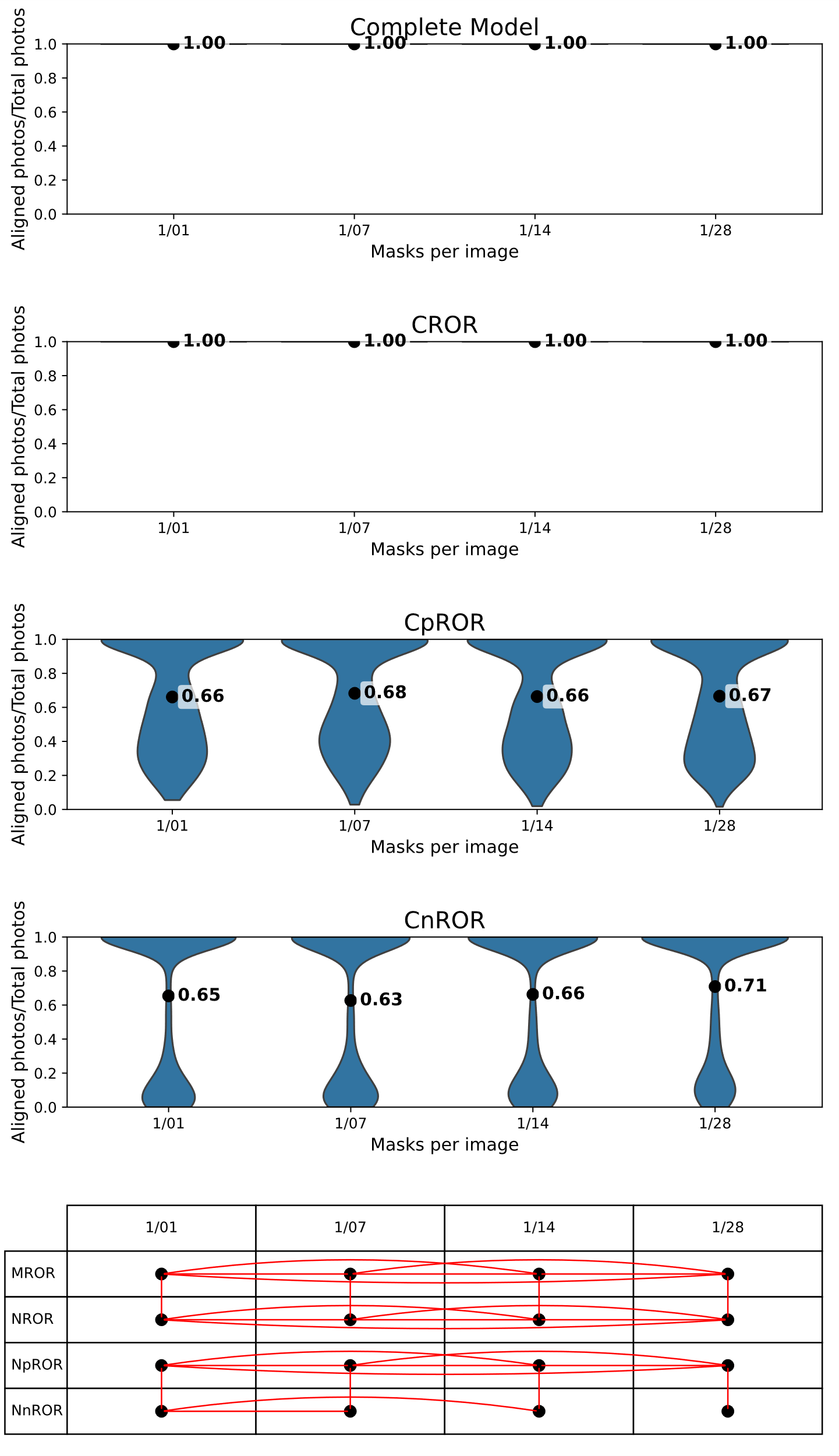

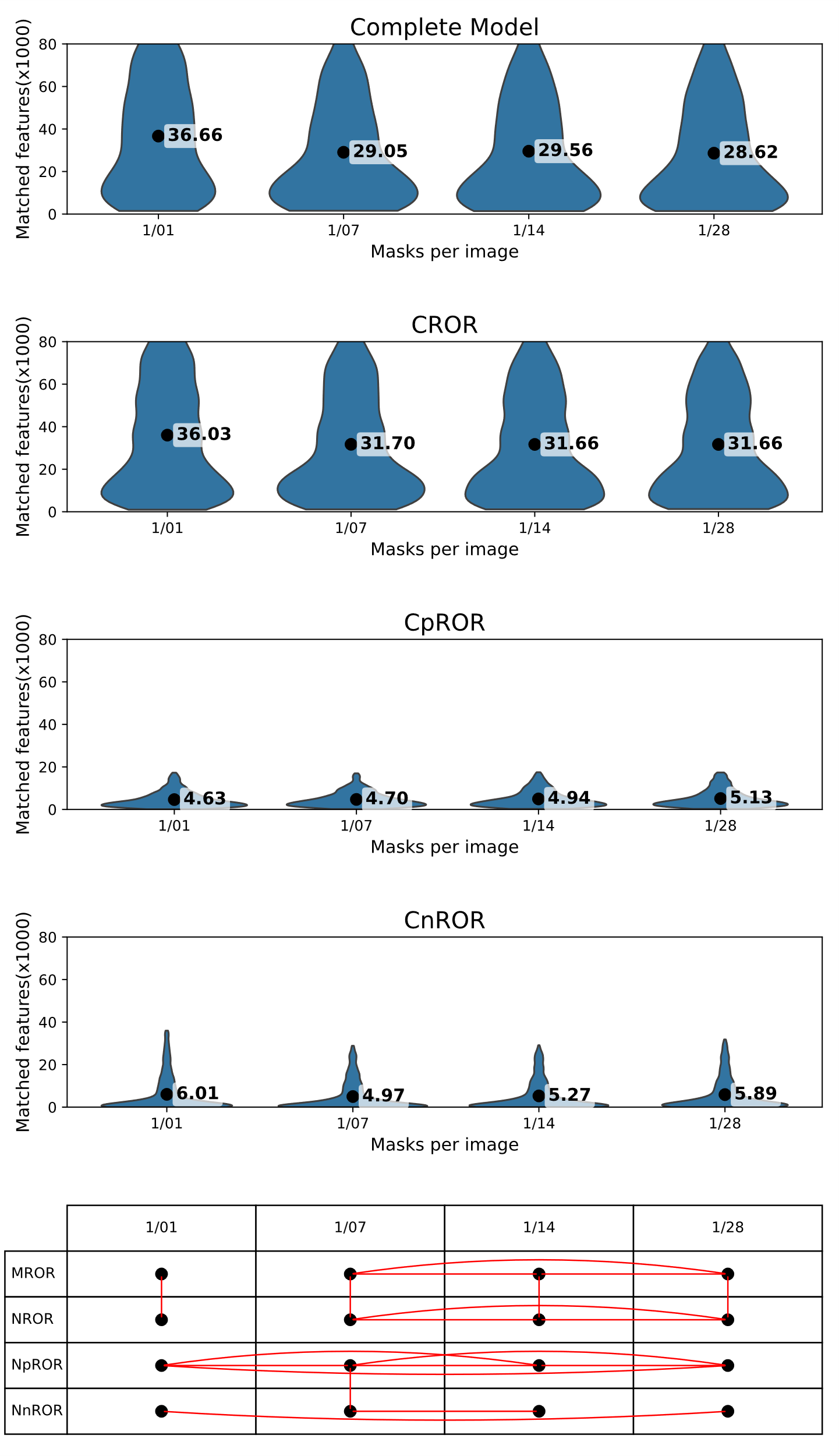


Fig. S36 Photo alignment ratio by masks per image. In both the complete model (MROR) and CROR, the alignment ratio remained constant at 1.00 across all mask configurations (1/01–1/28), with no observed variability (SD = 0; min = max = 1). Due to the absence of variation, statistical comparisons within MROR and CROR could not be computed. Likewise, no statistically significant differences were observed between MROR and CROR (p = 1). Red lines in the bottom matrix shows no significant correlations (p > 0.05).

Fig. S35 Number of matched features by masks per image. In the complete model (MROR), mean values decreased from 36.66 (1/01) to 28.62 (1/28), with intermediate values of 29.05 (1/07) and 29.56 (1/14). In CROR, values remained relatively stable across mask ratios, ranging from 31.66 to 36.03, with the highest value at 1/01 (36.03) and similar values at 1/07 (31.70), 1/14 (31.66), and 1/28 (31.66). Most pairwise comparisons within MROR and CROR were statistically significant (p < 0.001), except between 1/07–1/14, 1/07–1/28, and 1/14–1/28, where no statistically significant differences were observed (p = 1). No statistically significant differences were found between MROR and CROR across classifications. Red lines in the bottom matrix shows no significant correlations (p > 0.05).


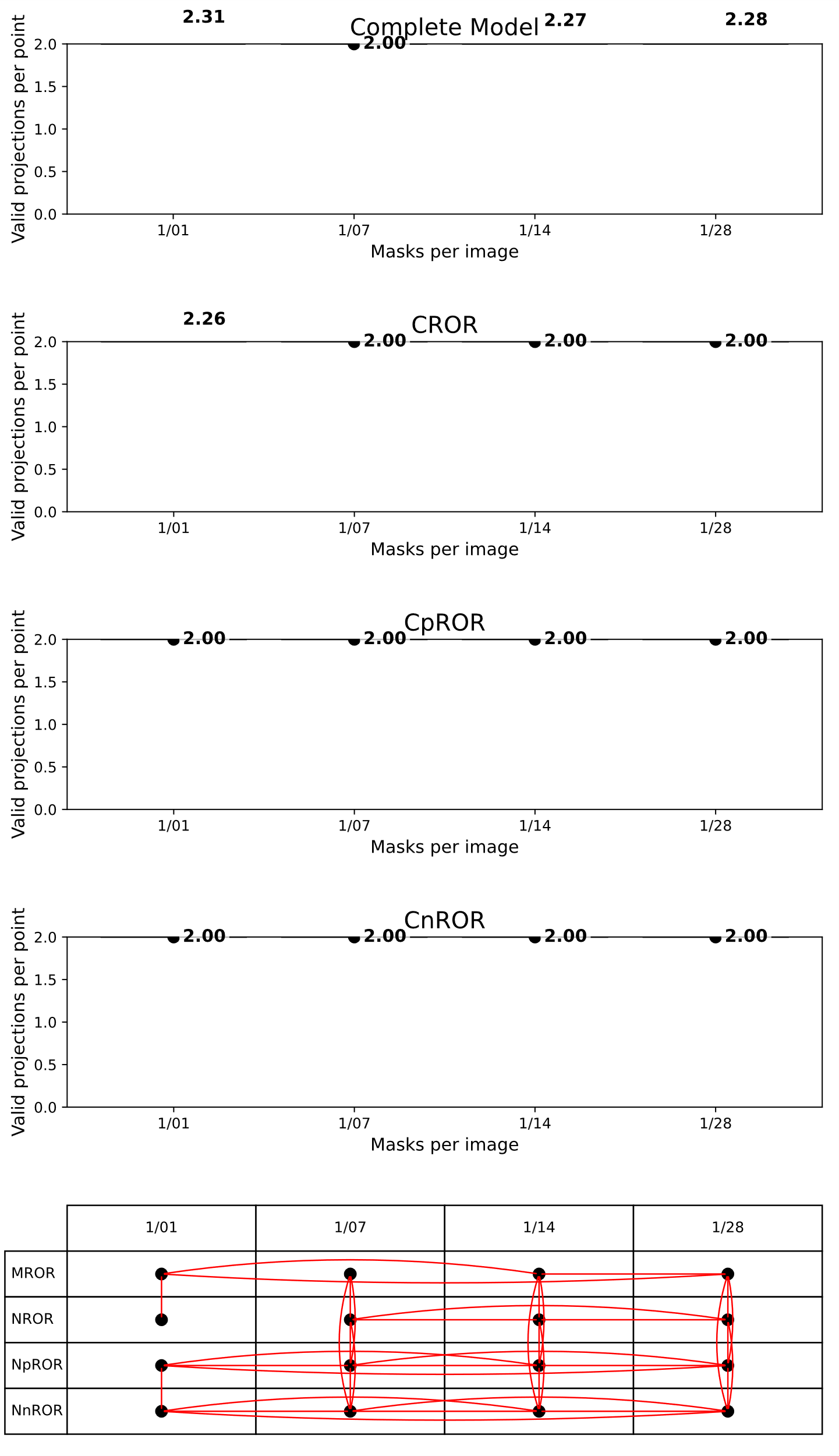

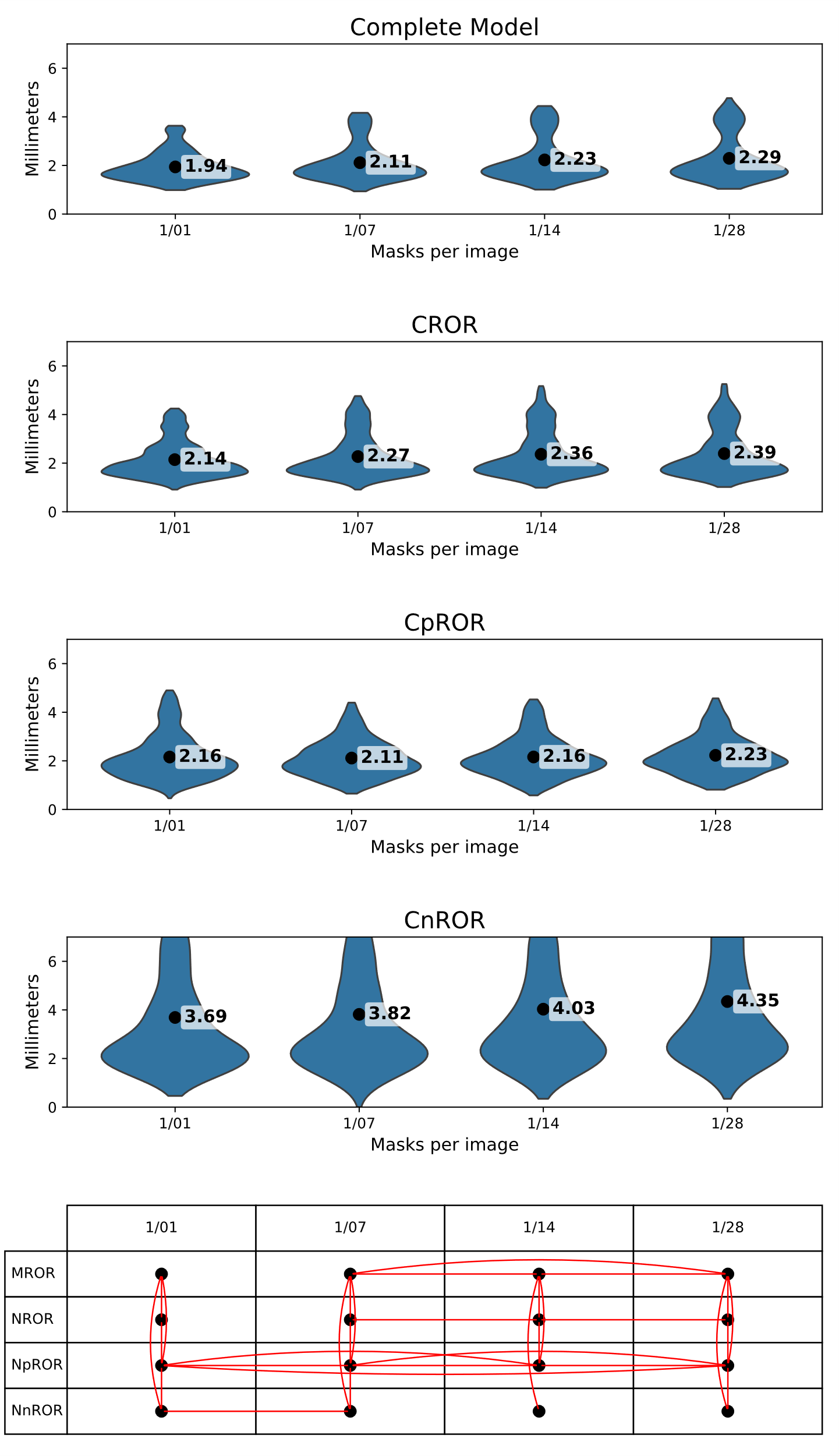


Fig. S38 Median of valid projections per feature point by masks per image. In the complete model (MROR), mean values were 2.31 (1/01), 2.00 (1/07), 2.27 (1/14), and 2.28 (1/28), with no variability at 1/07 (SD = 0; min = max = 2). In CROR, the mean value was 2.26 at 1/01, while remaining constant at 2.00 for 1/07, 1/14, and 1/28, also with no variability (SD = 0). Within MROR, statistically significant differences were observed between 1/01–1/07 and 1/07–1/14/1/28 (p < 0.001), whereas other comparisons showed no significant differences (p = 1). In CROR, significant differences were found between 1/01 and the remaining configurations, while comparisons among 1/07, 1/14, and 1/28 could not be computed due to identical distributions. No statistically significant differences were observed between MROR and CROR across classifications. Red lines in the bottom matrix shows no significant correlations (p > 0.05).

Fig. S37 RMS of point error per matched feature (mm) by masks per image. In the complete model (MROR), mean RMS values increased slightly with mask density, from 1.94 mm (1/01) to 2.29 mm (1/28), with intermediate values of 2.11 mm (1/07) and 2.23 mm (1/14). A similar trend was observed in CROR, where values increased from 2.14 mm (1/01) to 2.39 mm (1/28), with intermediate values of 2.27 mm (1/07) and 2.36 mm (1/14). Overall, variability increased moderately with higher mask ratios. Most pairwise comparisons within MROR and CROR were statistically significant (p < 0.001), except between adjacent higher mask configurations (e.g., 1/07–1/14, 1/07–1/28, and 1/14–1/28 in MROR; 1/07–1/14 and 1/14–1/28 in CROR). No statistically significant differences were observed between MROR and CROR across classifications. Red lines in the bottom matrix shows no significant correlations (p > 0.05).


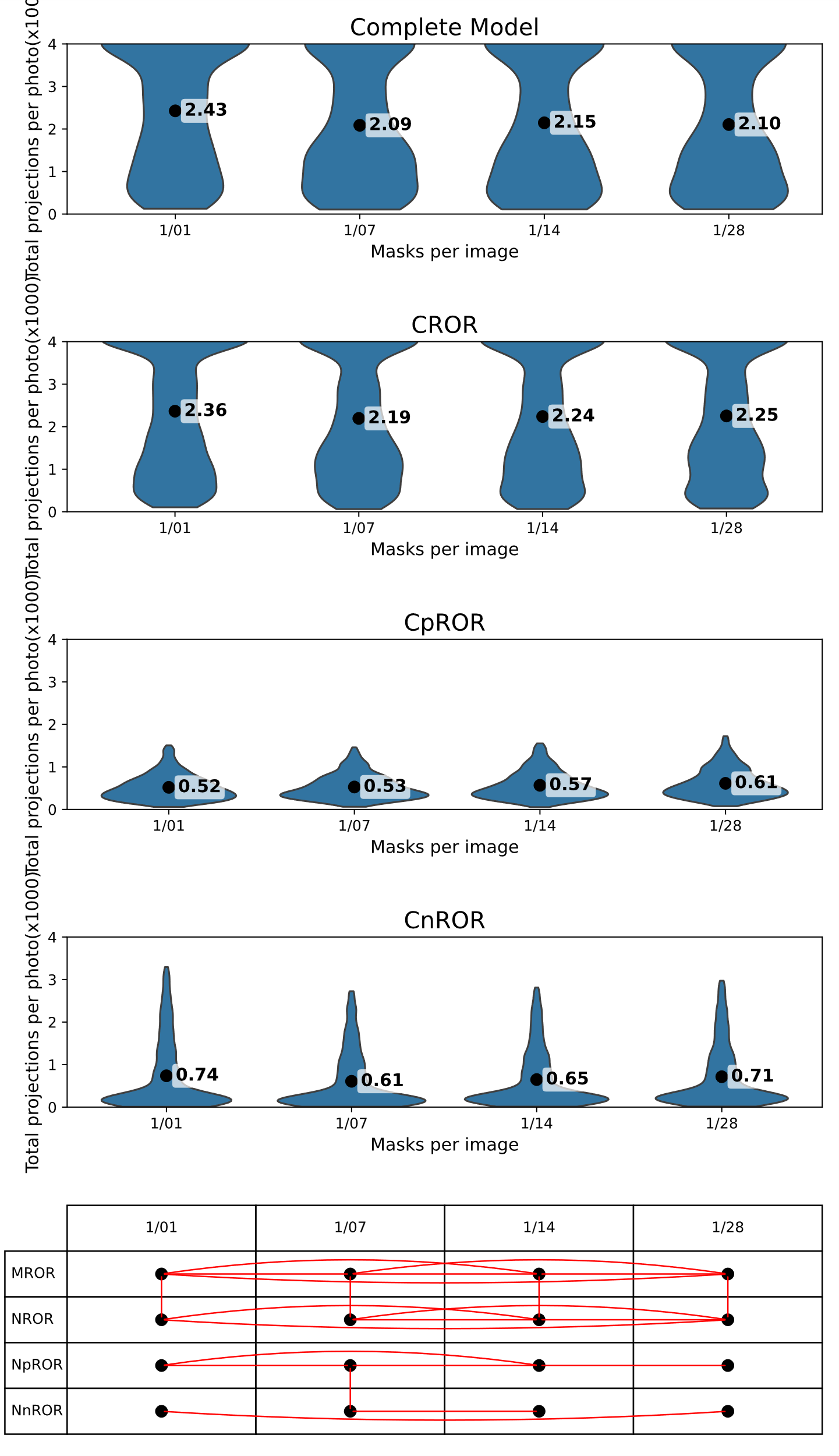

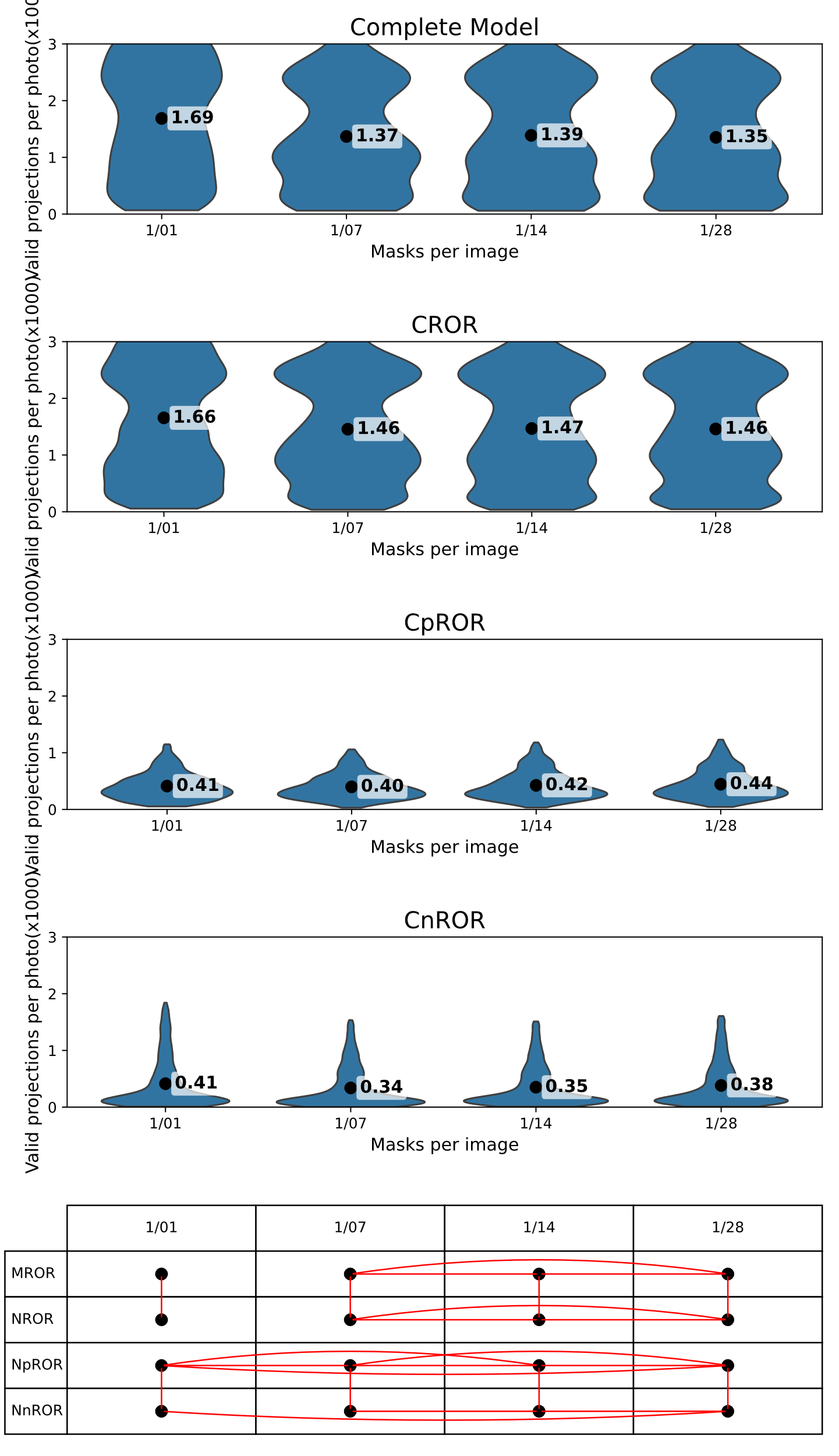


Fig. S40 Median total projections per photo by masks per image. In the complete model (MROR), mean values showed a slight decrease from 2.43 (1/01) to 2.10 (1/28), with intermediate values of 2.09 (1/07) and 2.15 (1/14). In CROR, values remained relatively stable, ranging from 2.19 to 2.36, with a slight increase toward 1/28 (2.25). Most pairwise comparisons within MROR were not statistically significant (p = 1), with only minor differences observed (e.g., 1/01 vs. 1/07). In CROR, a statistically significant difference was observed between 1/01–1/07 (p < 0.001), while other comparisons were not significant. No statistically significant differences were found between MROR and CROR across classifications. Red lines in the bottom matrix shows no significant correlations (p > 0.05).

Fig. S39 Median of valid projections per photo by masks per image. In the complete model (MROR), mean values decreased from 1.69 (1/01) to 1.35 (1/28), with intermediate values of 1.37 (1/07) and 1.39 (1/14). In CROR, a similar pattern was observed, with values decreasing from 1.66 (1/01) to approximately 1.46 (1/07–1/28). Most statistically significant differences within both MROR and CROR were observed when comparing 1/01 with the remaining configurations (p < 0.001), whereas no significant differences were found among 1/07, 1/14, and 1/28 (p = 1). No statistically significant differences were observed between MROR and CROR across classifications. Red lines in the bottom matrix shows no significant correlations (p > 0.05).


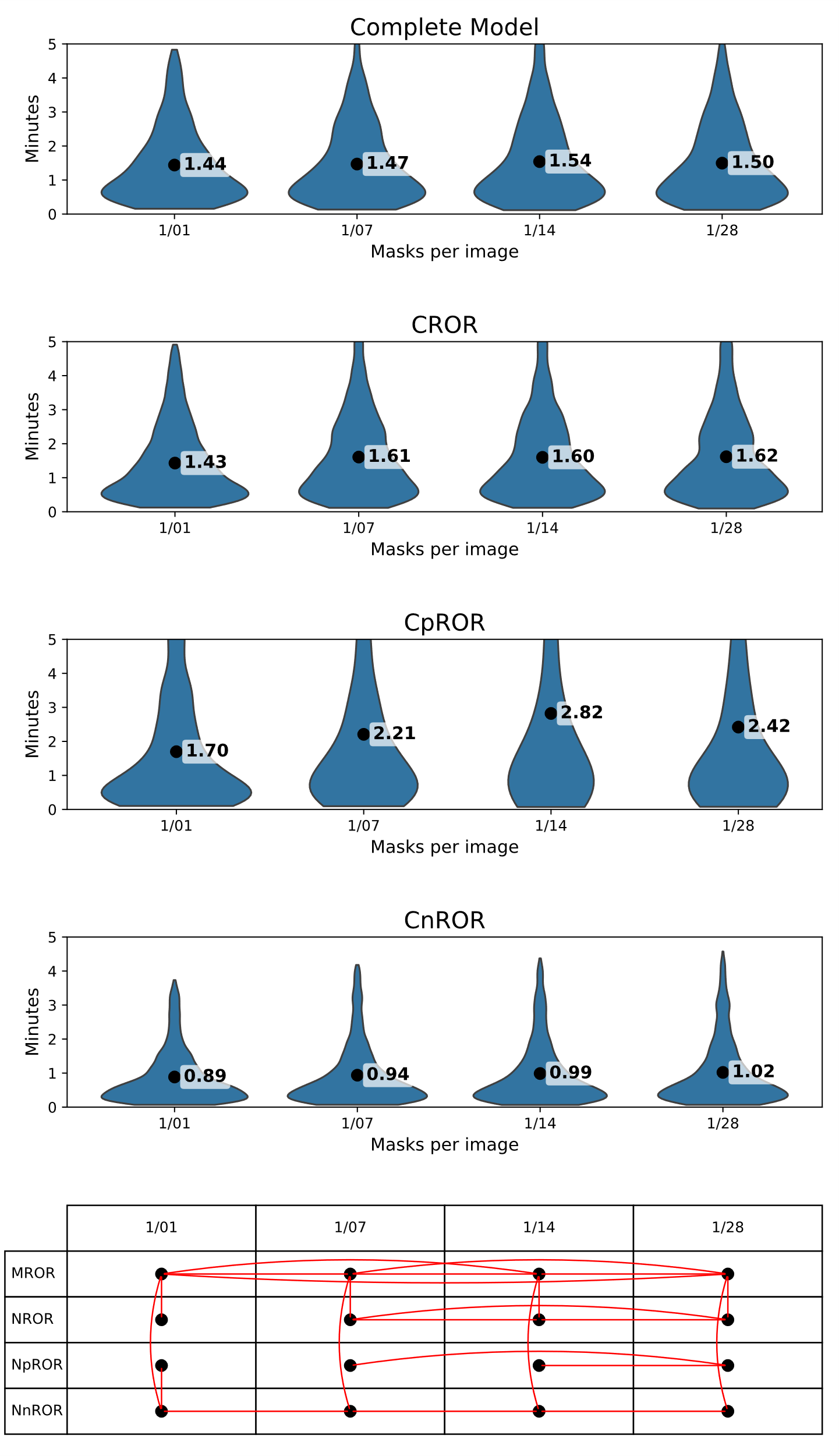

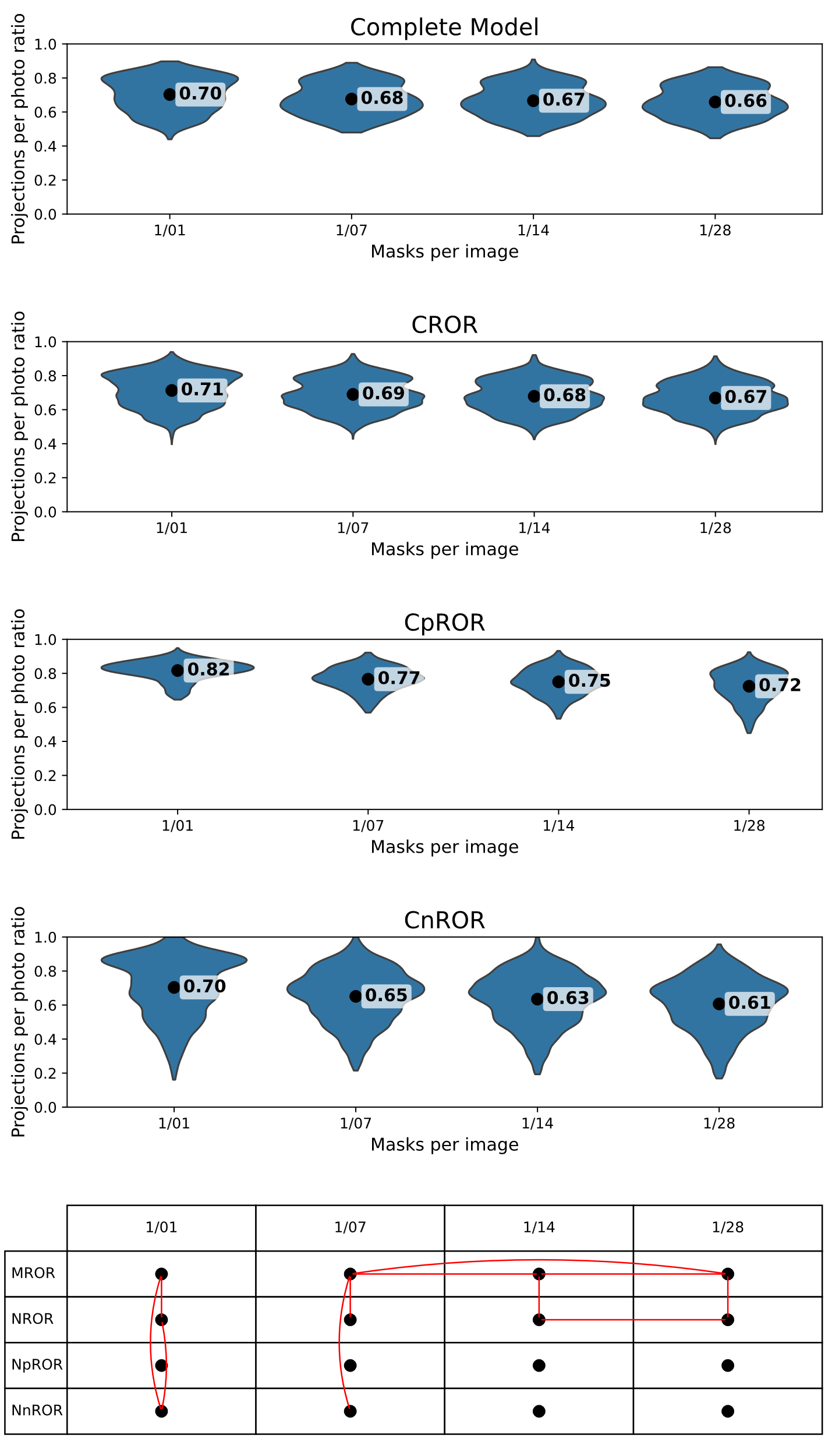


Fig. S42 Total SfM processing time (minutes) by masks per image. In the complete model (MROR), mean processing time remained relatively stable across mask configurations, ranging from 1.44 min (1/01) to 1.54 min (1/14), with a slight decrease at 1/28 (1.50 min). In CROR, mean processing time increased slightly from 1.43 min (1/01) to 1.62 min (1/28), with intermediate values of 1.61 min (1/07) and 1.60 min (1/14). Within MROR, no statistically significant differences were observed across classifications (p = 1). In CROR, statistically significant differences were found when comparing 1/01 with the remaining configurations, while other comparisons were not significant. No statistically significant differences were observed between MROR and CROR across classifications. Red lines in the bottom matrix shows no significant correlations (p > 0.05).

Fig. S41 Median projections per photo ratio by masks per image. In the complete model (MROR), mean values showed a slight decreasing trend with increasing mask density, from 0.70 (1/01) to 0.66 (1/28), with intermediate values of 0.68 (1/07) and 0.67 (1/14). A similar pattern was observed in CROR, with values decreasing from 0.71 (1/01) to 0.67 (1/28). Most pairwise comparisons within MROR were not statistically significant (p = 1), except between 1/01–1/14 and 1/01–1/28 (p < 0.001). In CROR, most comparisons were statistically significant, except among 1/07, 1/14, and 1/28 (p = 0.11). No statistically significant differences were observed between MROR and CROR across classifications. Red lines in the bottom matrix shows no significant correlations (p > 0.05).


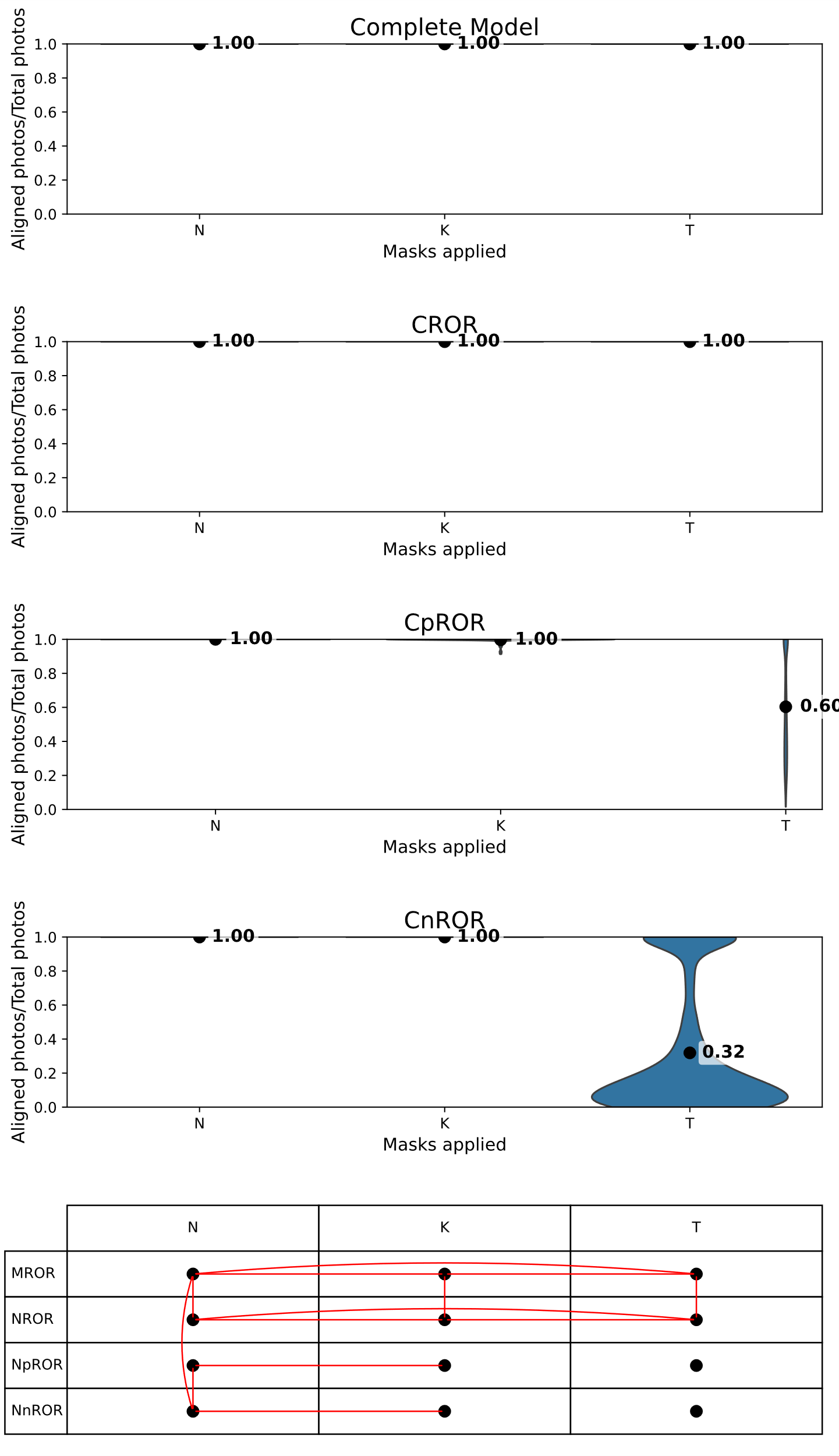

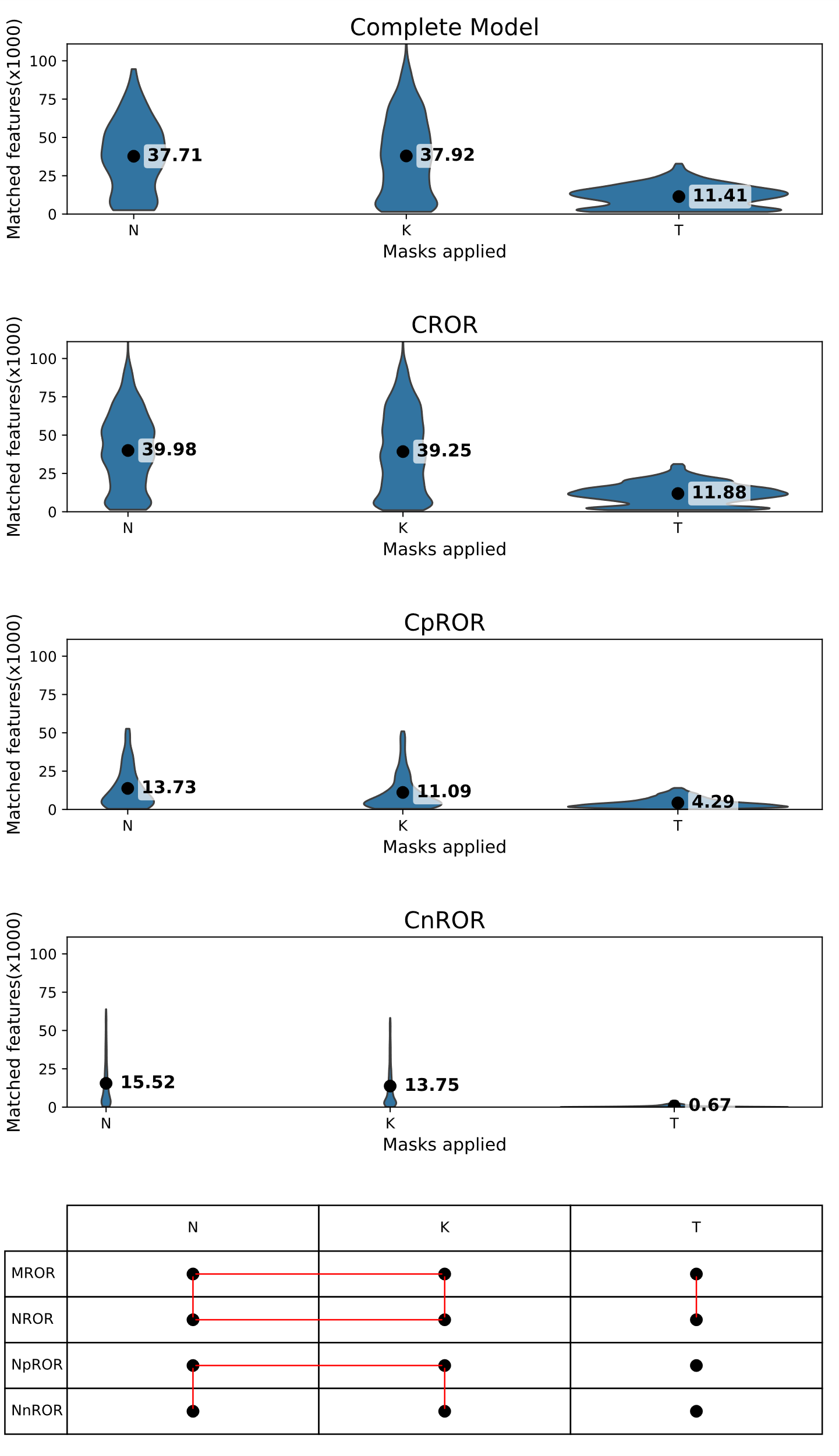


Fig. S44 Photo alignment ratio by mask type. In both the complete model (MROR) and CROR, the alignment ratio remained constant at 1.00 across all mask types (N, K, and T), with no observed variability (SD = 0; min = max = 1). Due to the absence of variation, statistical comparisons within MROR and CROR could not be computed. Likewise, no statistically significant differences were observed between MROR and CROR (p = 1). Red lines in the bottom matrix shows no significant correlations (p > 0.05).

Fig. S43 Number of matched features by mask type. In the complete model (MROR), mean values were similar for N (37.71) and K (37.92), while a marked decrease was observed for T (11.41). In CROR, a comparable pattern was observed, with similar values for N (39.98) and K (39.25), and lower values for T (11.88). Most pairwise comparisons within MROR and CROR were statistically significant (p < 0.001), except between N and K, where no statistically significant differences were observed (p = 1). No statistically significant differences were found between MROR and CROR across classifications. Red lines in the bottom matrix shows no significant correlations (p > 0.05).


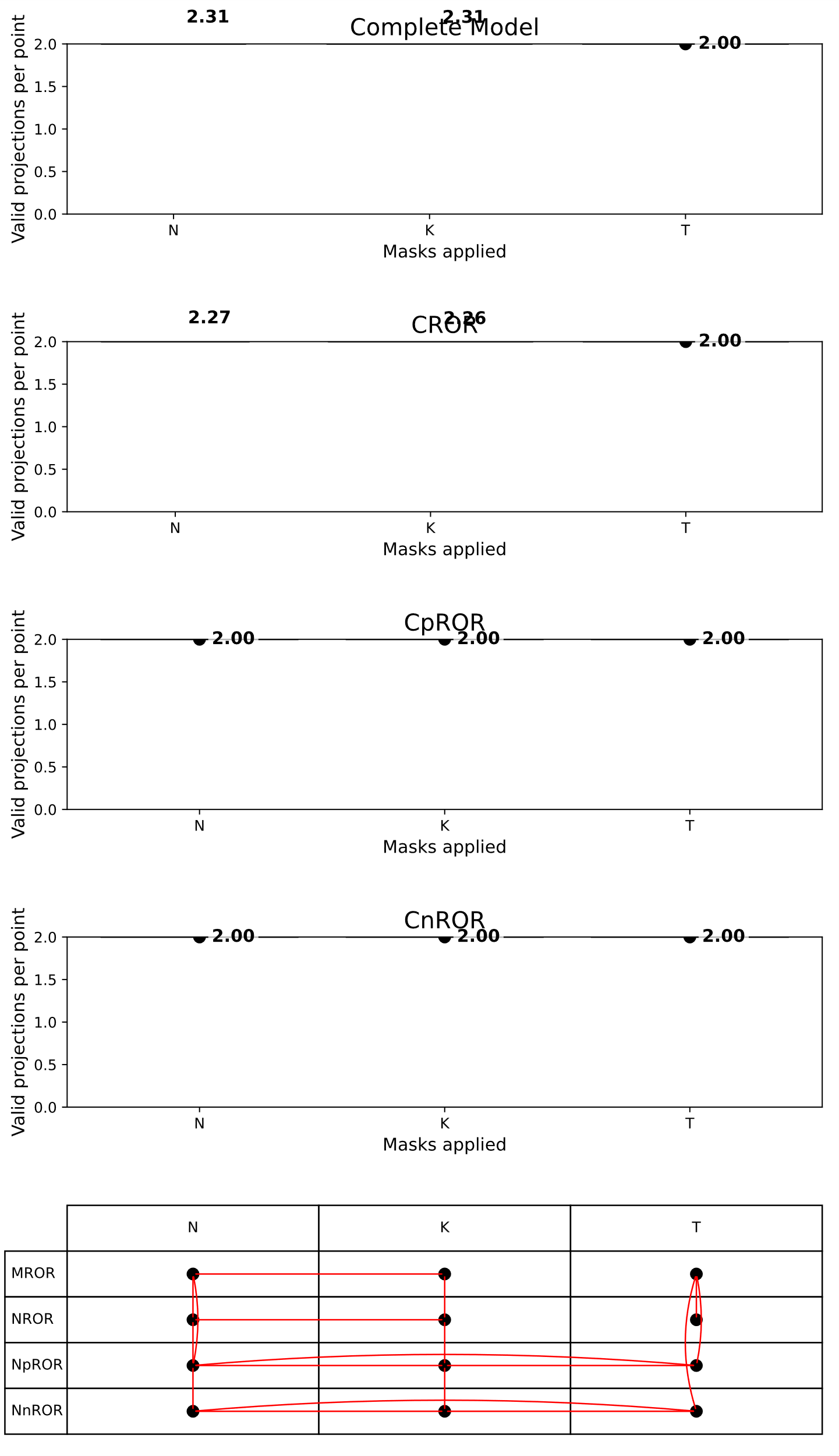

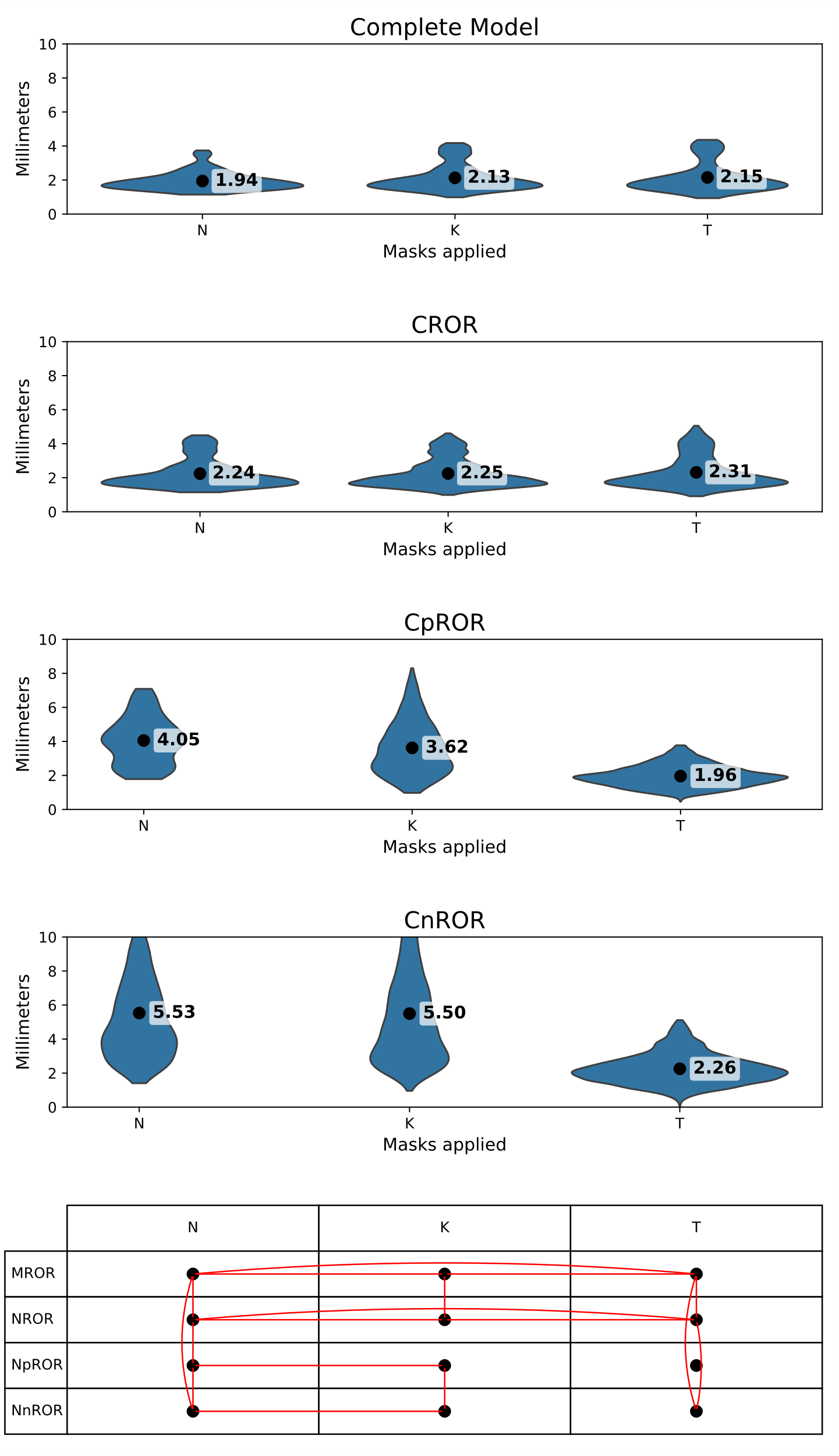


Fig. S46 Median of valid projections per feature point by mask type. In the complete model (MROR), mean values were 2.31 for both N and K, and decreased to 2.00 for T, with no variability in the latter (SD = 0). In CROR, mean values were 2.27 (N), 2.26 (K), and 2.00 (T), also showing no variability for T (SD = 0). Within both MROR and CROR, statistically significant differences were observed when comparing N–T and K–T (p < 0.001), whereas no significant differences were found between N–K (p = 1). No statistically significant differences were observed between MROR and CROR across classifications. Red lines in the bottom matrix shows no significant correlations (p > 0.05).

Fig. S45 RMS of point error per matched feature (mm) by mask type. In the complete model (MROR), mean values increased slightly across mask types, from 1.94 mm (N) to 2.15 mm (T), with an intermediate value of 2.13 mm (K). A similar pattern was observed in CROR, with values ranging from 2.24 mm (N) to 2.31 mm (T), and 2.25 mm for K. Overall, variability was comparable across mask types and between methods. No statistically significant differences were observed within MROR or CROR (p = 1), nor between MROR and CROR across classifications. Red lines in the bottom matrix shows no significant correlations (p > 0.05).


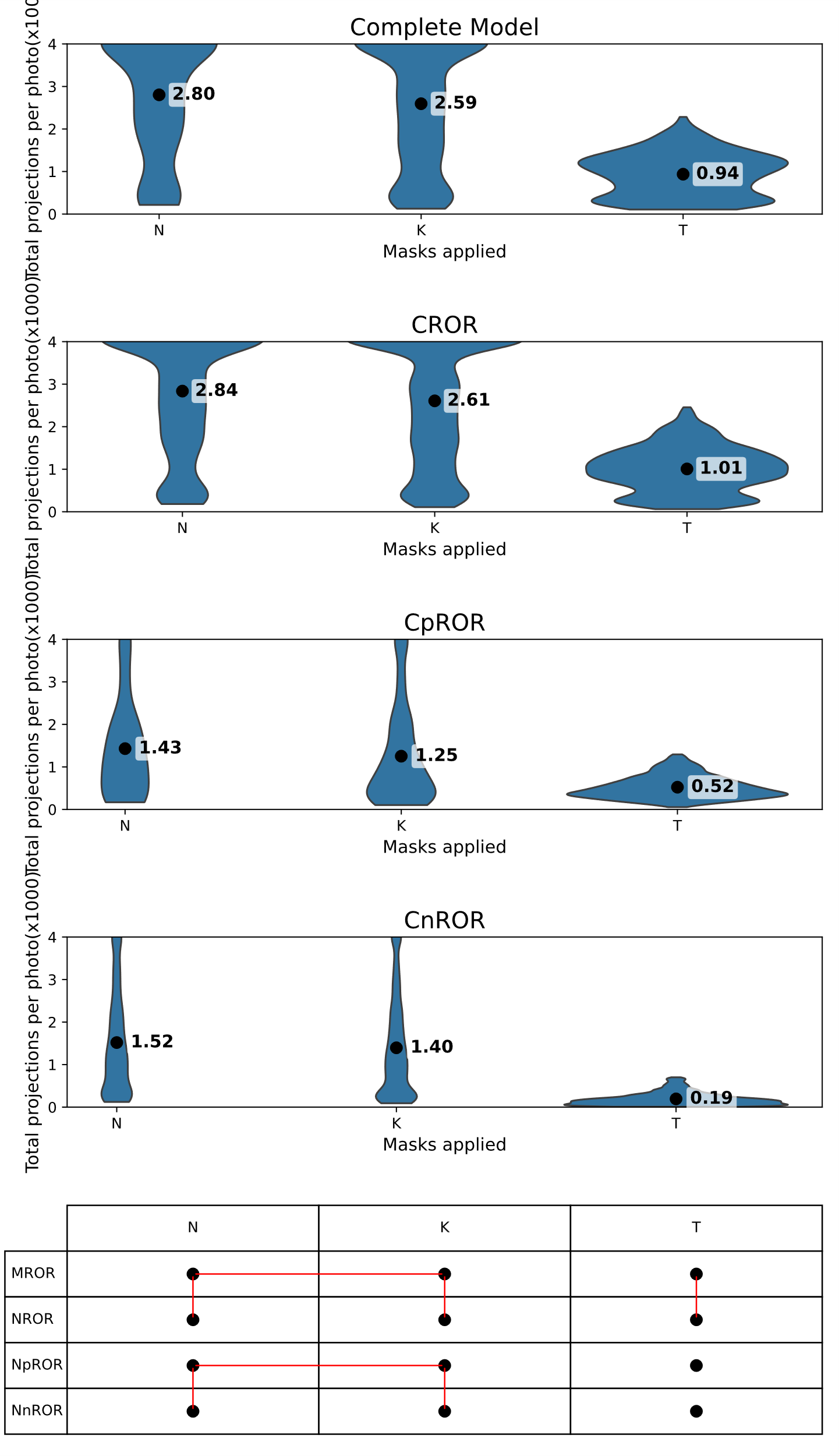

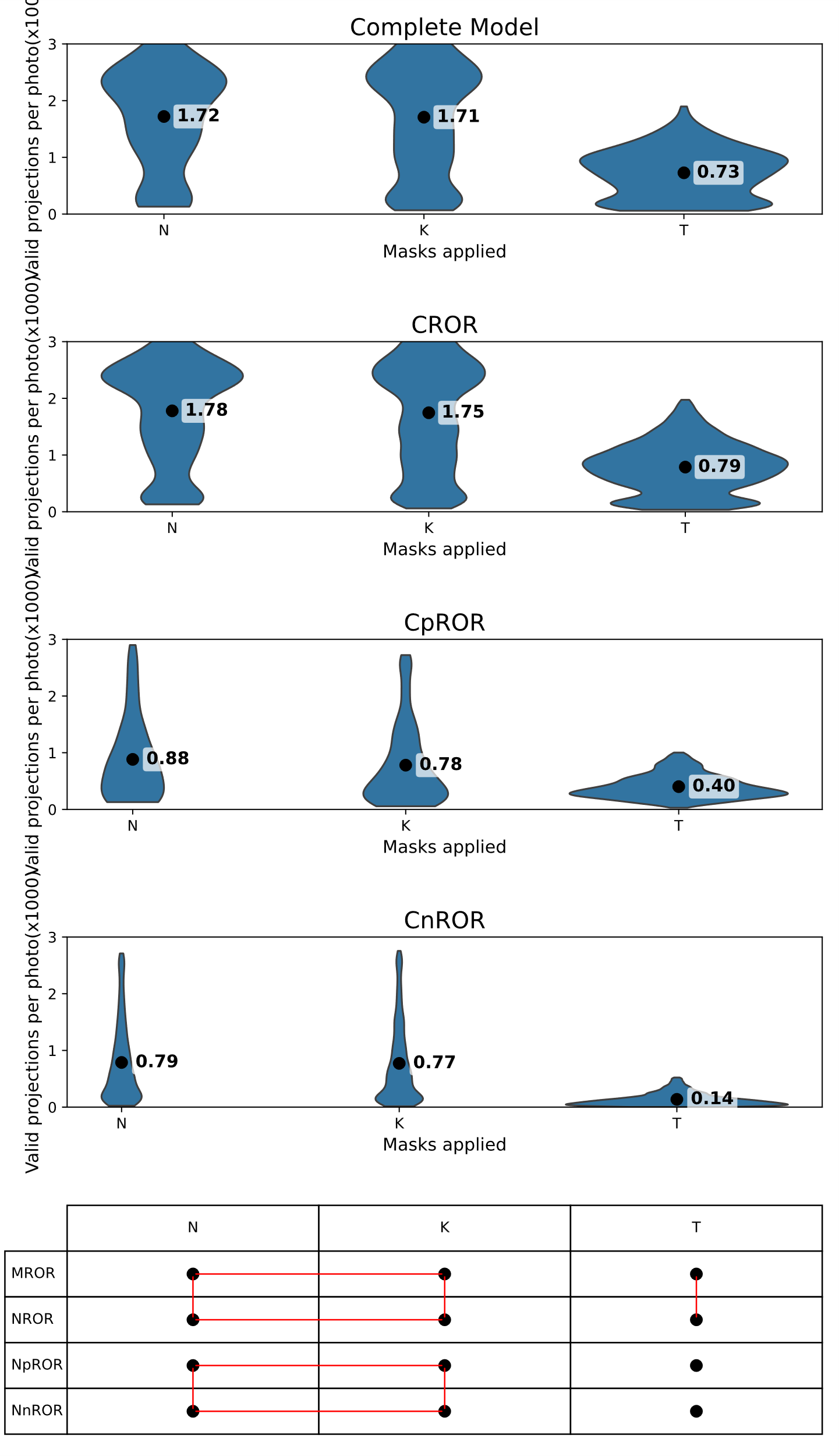


Fig. S48 Median total projections per photo by mask type.. In the complete model (MROR), mean values were 2.80 (N), 2.59 (K), and decreased markedly to 0.94 (T). A similar pattern was observed in CROR, with values of 2.84 (N), 2.61 (K), and 1.01 (T). Within MROR, statistically significant differences were found between N–T and K–T (p < 0.001), while no significant differences were observed between N–K (p = 1). In CROR, all pairwise comparisons showed statistically significant differences. No statistically significant differences were observed between MROR and CROR across classifications. Red lines in the bottom matrix shows no significant correlations (p > 0.05).

Fig. S47 Median of valid projections per photo by mask type. In the complete model (MROR), mean values were similar for N (1.72) and K (1.71), while a marked decrease was observed for T (0.73). A comparable pattern was observed in CROR, with values of 1.78 (N), 1.75 (K), and 0.79 (T). Within both MROR and CROR, statistically significant differences were observed when comparing N–T and K–T (p < 0.001), whereas no significant differences were found between N–K (p = 1). No statistically significant differences were observed between MROR and CROR across classifications. Red lines in the bottom matrix shows no significant correlations (p > 0.05).


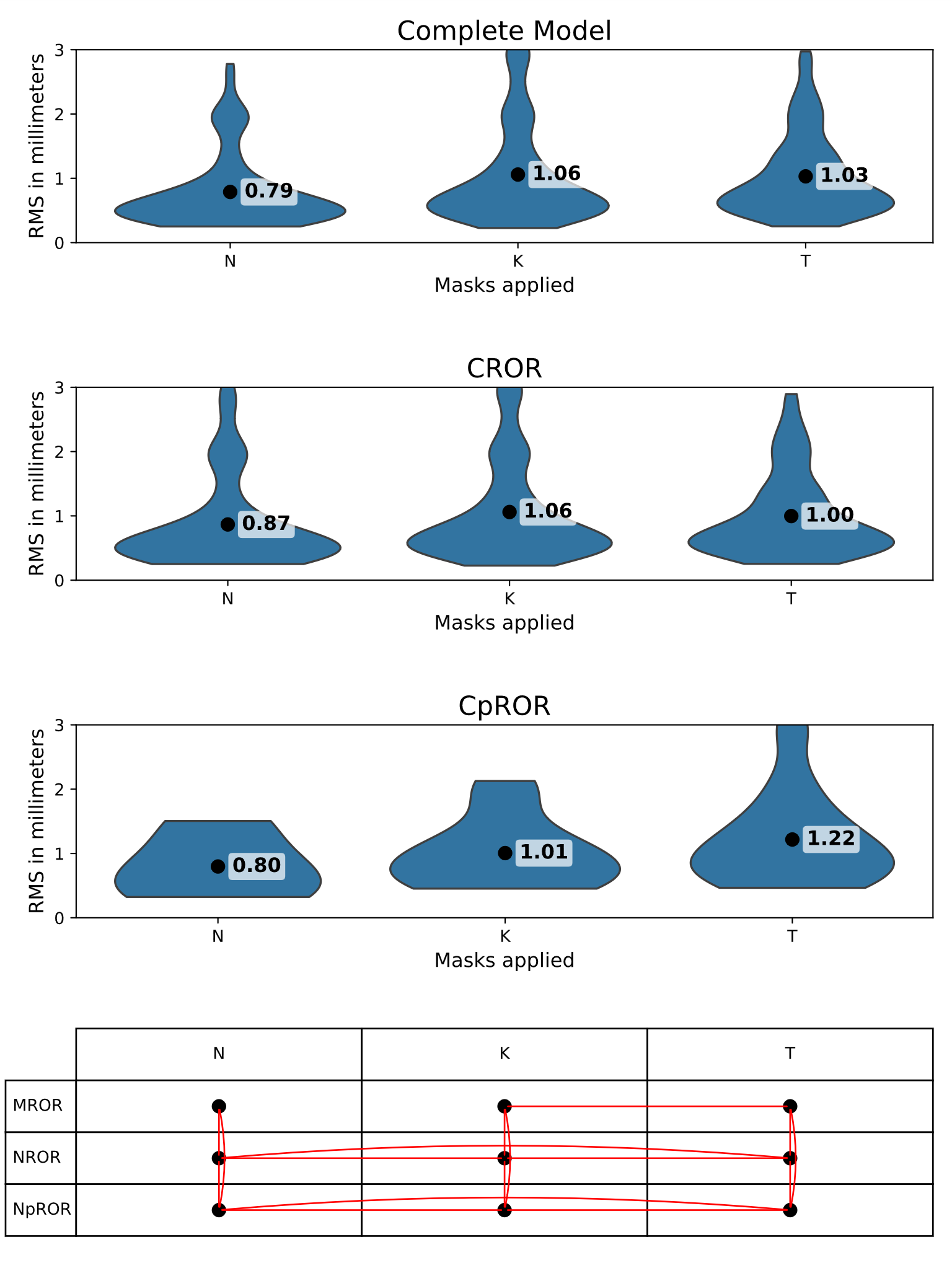

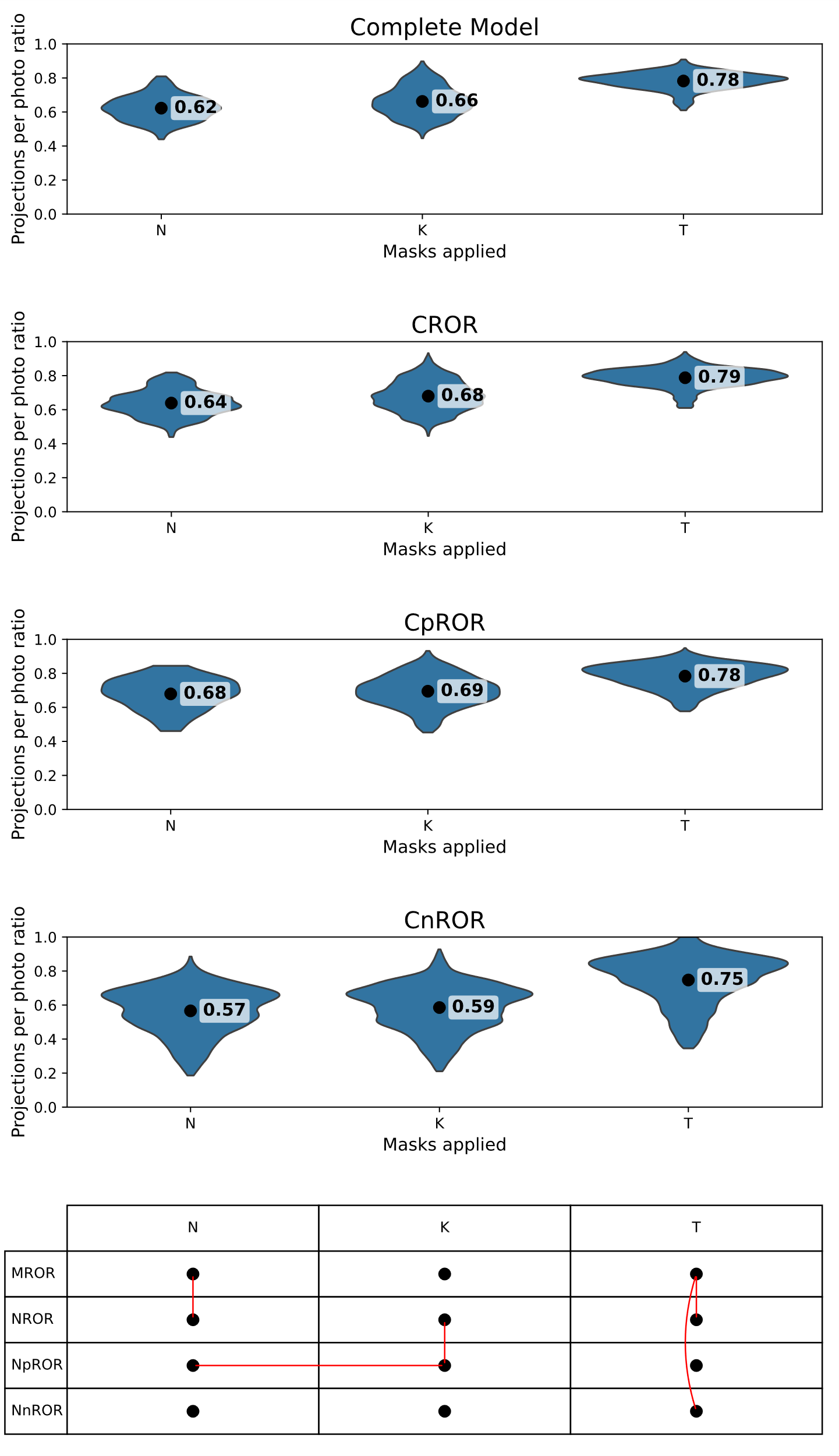


Fig. S50 RMS of vertex distance from ground truth (mm) by mask type. In the complete model (MROR), mean RMS values were 0.79 mm (N), 1.06 mm (K), and 1.03 mm (T), indicating slightly higher errors for K and T compared to N. A similar pattern was observed in CROR, with values of 0.87 mm (N), 1.06 mm (K), and 1.00 mm (T). Overall, variability was comparable across mask types and between methods. No statistically significant differences were observed within MROR or CROR (p = 1), nor between MROR and CROR across classifications. Red lines in the bottom matrix shows no significant correlations (p > 0.05).

Fig. S49 Median projections per photo ratio by mask type. In the complete model (MROR), mean values increased from 0.62 (N) to 0.78 (T), with an intermediate value of 0.66 (K). A similar increasing trend was observed in CROR, with values of 0.64 (N), 0.68 (K), and 0.79 (T). Within both MROR and CROR, all pairwise comparisons showed statistically significant differences (p < 0.001). When comparing between methods, no statistically significant differences were found for N and T (p = 1), whereas a significant difference was observed for K (p < 0.001). Red lines in the bottom matrix shows no significant correlations (p > 0.05).


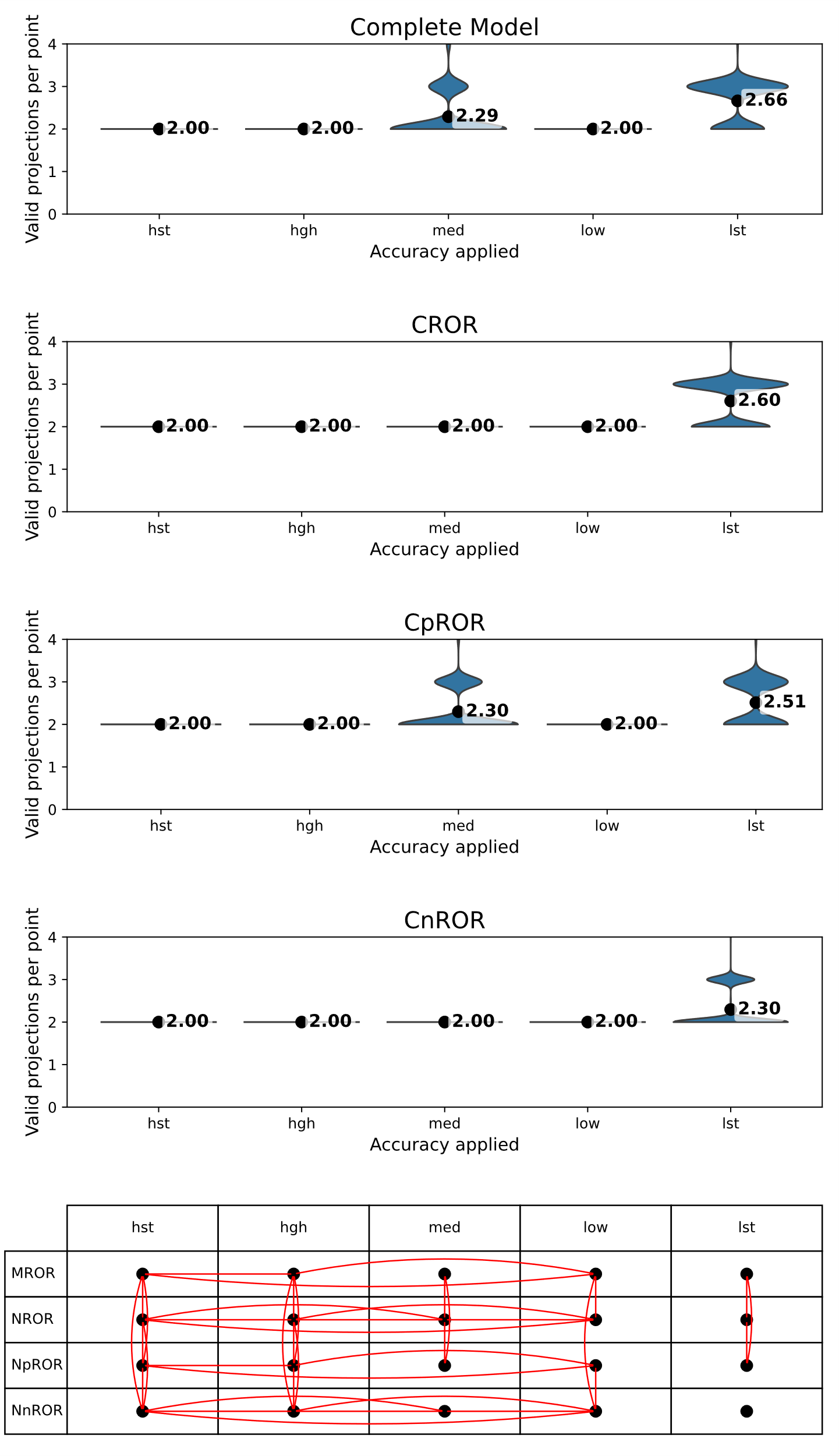

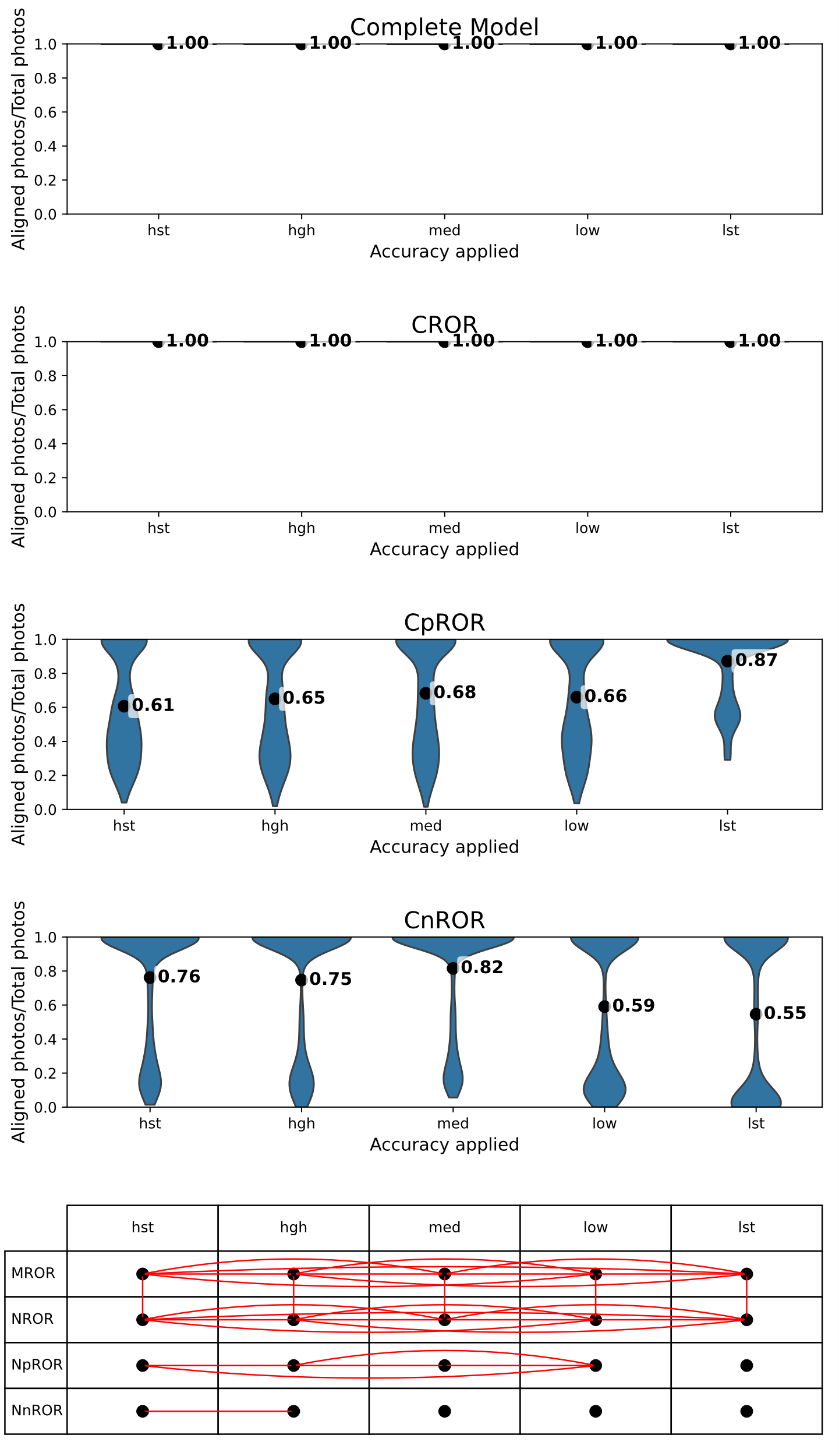


Fig. S52 Median of valid projections per feature point by accuracy level. In the complete model (MROR), values remained constant at 2.00 for highest (hst), high (hgh), and low, with no variability (SD = 0), while increasing to 2.29 at medium (med) and 2.66 at lowest (lst). In CROR, values were constant at 2.00 across hst, hgh, med, and low, and increased to 2.60 at lst. Within MROR, most comparisons were statistically significant (p < 0.001), except among hst, hgh, and low, where comparisons could not be computed due to identical distributions. In CROR, most comparisons could not be computed due to identical distributions, except for comparisons involving lst (e.g., hst–lst, med–low, low–lst), which showed statistically significant differences. No statistically significant differences were observed between MROR and CROR across classifications (p = 1). Red lines in the bottom matrix shows no significant correlations (p > 0.05).

Fig. S51 Photo alignment ratio by accuracy level. In both the complete model (MROR) and CROR, the alignment ratio remained constant at 1.00 across all accuracy levels (highest, high, medium, low, lowest), with no observed variability (SD = 0; min = max = 1). Due to the absence of variation, statistical comparisons within MROR and CROR could not be computed. Likewise, no statistically significant differences were observed between MROR and CROR (p = 1). Red lines in the bottom matrix shows no significant correlations (p > 0.05).


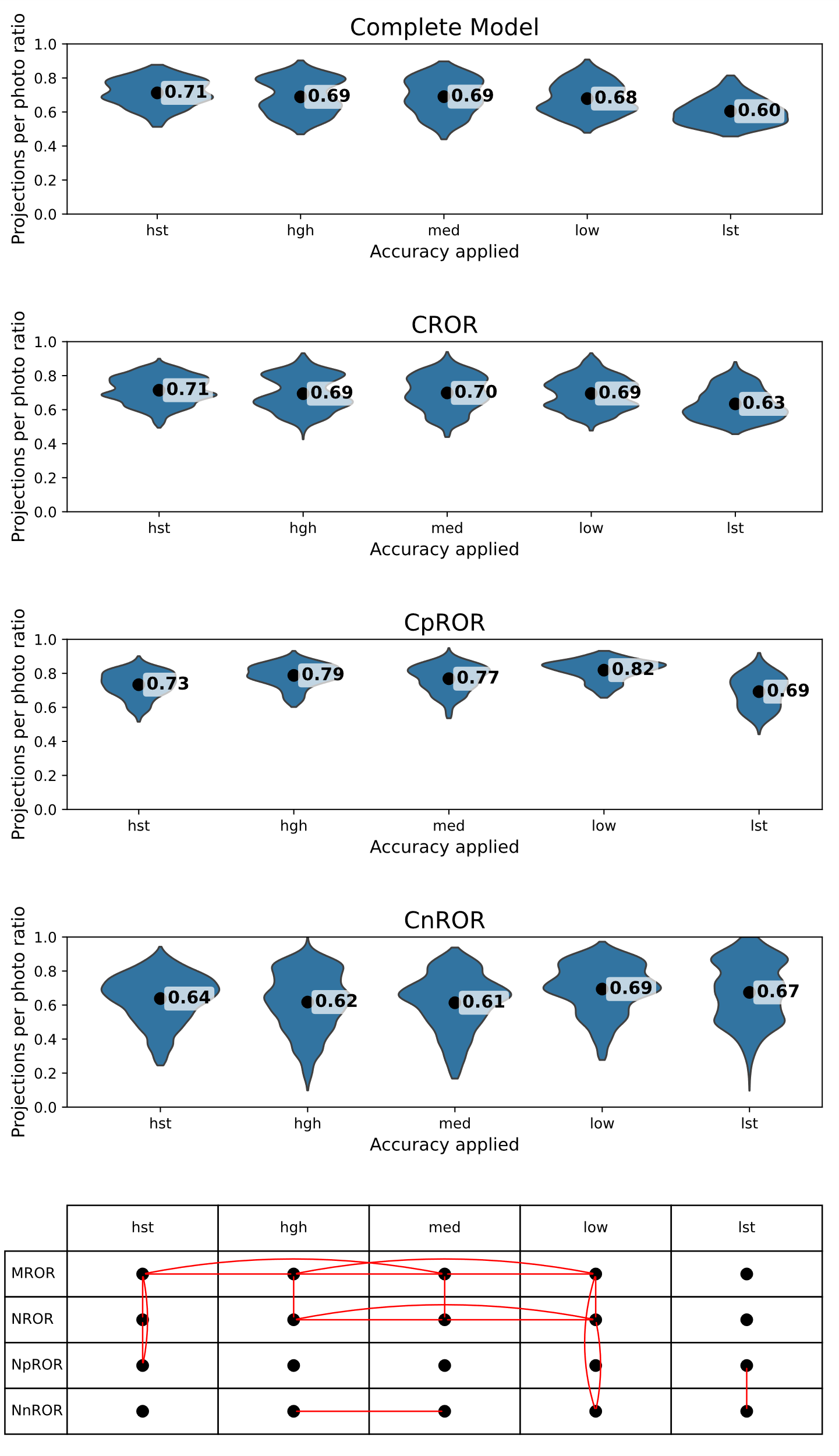

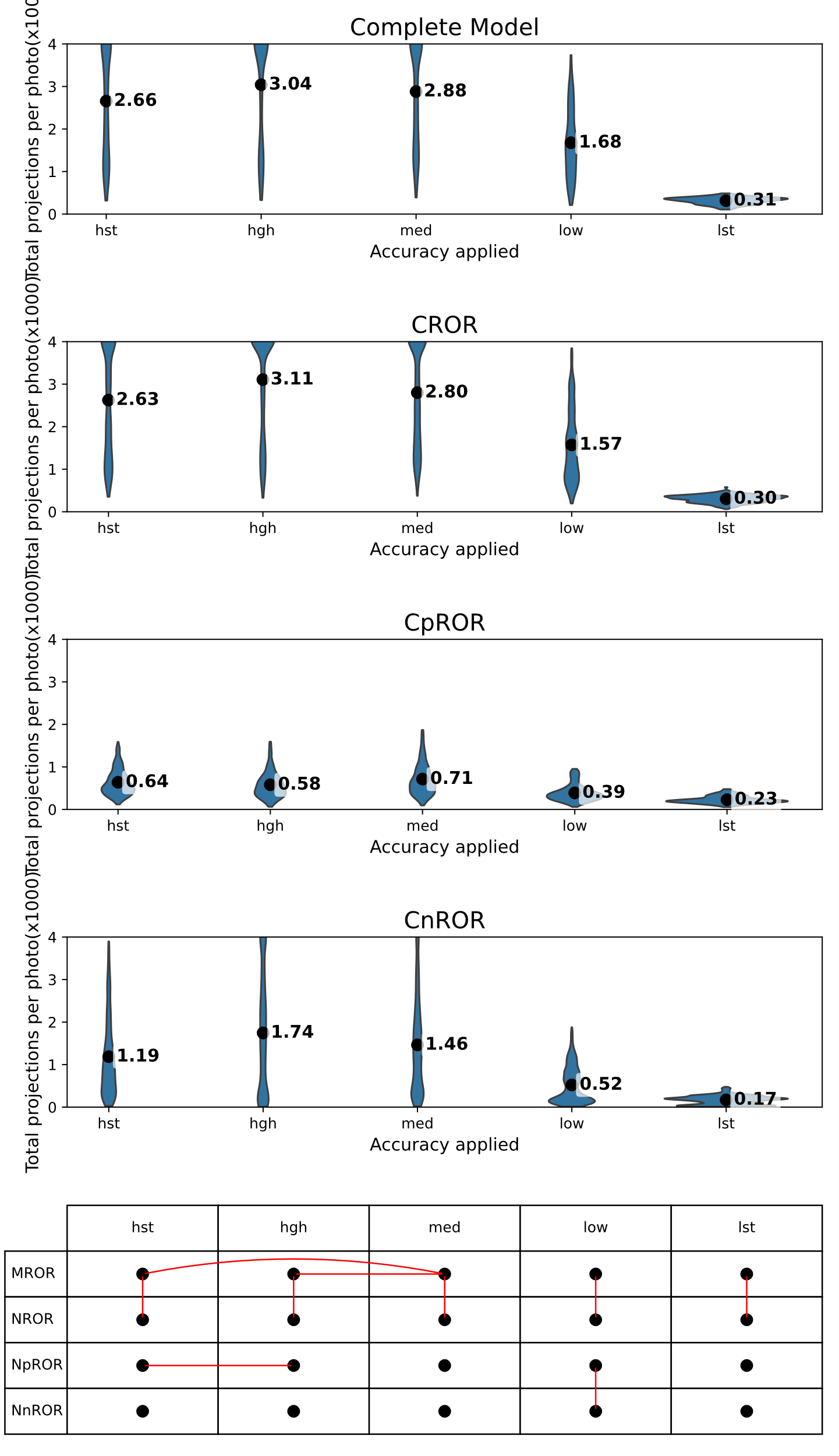


Fig. S54 Median projections per photo ratio by accuracy level. In the complete model (MROR), mean values showed a slight decreasing trend from 0.71 (highest) to 0.60 (lowest), with intermediate values of 0.69 (high and medium) and 0.68 (low). In CROR, a similar pattern was observed, with values ranging from 0.71 (highest) to 0.63 (lowest), and intermediate values of 0.69–0.70. Within MROR, most comparisons were not statistically significant (p = 1), except for comparisons involving lower accuracy levels (e.g., highest–low/lowest, medium–lowest, low–lowest; p < 0.001). In CROR, most comparisons were statistically significant (p < 0.001), except between adjacent levels (high–medium, high–low, and medium–low; p = 1). Between methods, no statistically significant differences were observed in most classifications, except at the lowest accuracy level (p < 0.001). Red lines in the bottom matrix shows no significant correlations (p > 0.05).

Fig. S53 Median total projections per photo by accuracy level. In the complete model (MROR), mean values were highest at high (3.04), followed by medium (2.88) and highest (2.66), and decreased markedly at low (1.68) and lowest (0.31). A similar pattern was observed in CROR, with values of 3.11 (high), 2.80 (medium), 2.63 (highest), decreasing to 1.57 (low) and 0.30 (lowest). Within MROR, most comparisons were statistically significant (p < 0.001), with no significant differences between highest–medium and high–medium, and a minor difference between highest–high (p = 0.01). In CROR, all pairwise comparisons were statistically significant (p < 0.001). No statistically significant differences were observed between MROR and CROR across classifications (p = 1). Red lines in the bottom matrix shows no significant correlations (p > 0.05).


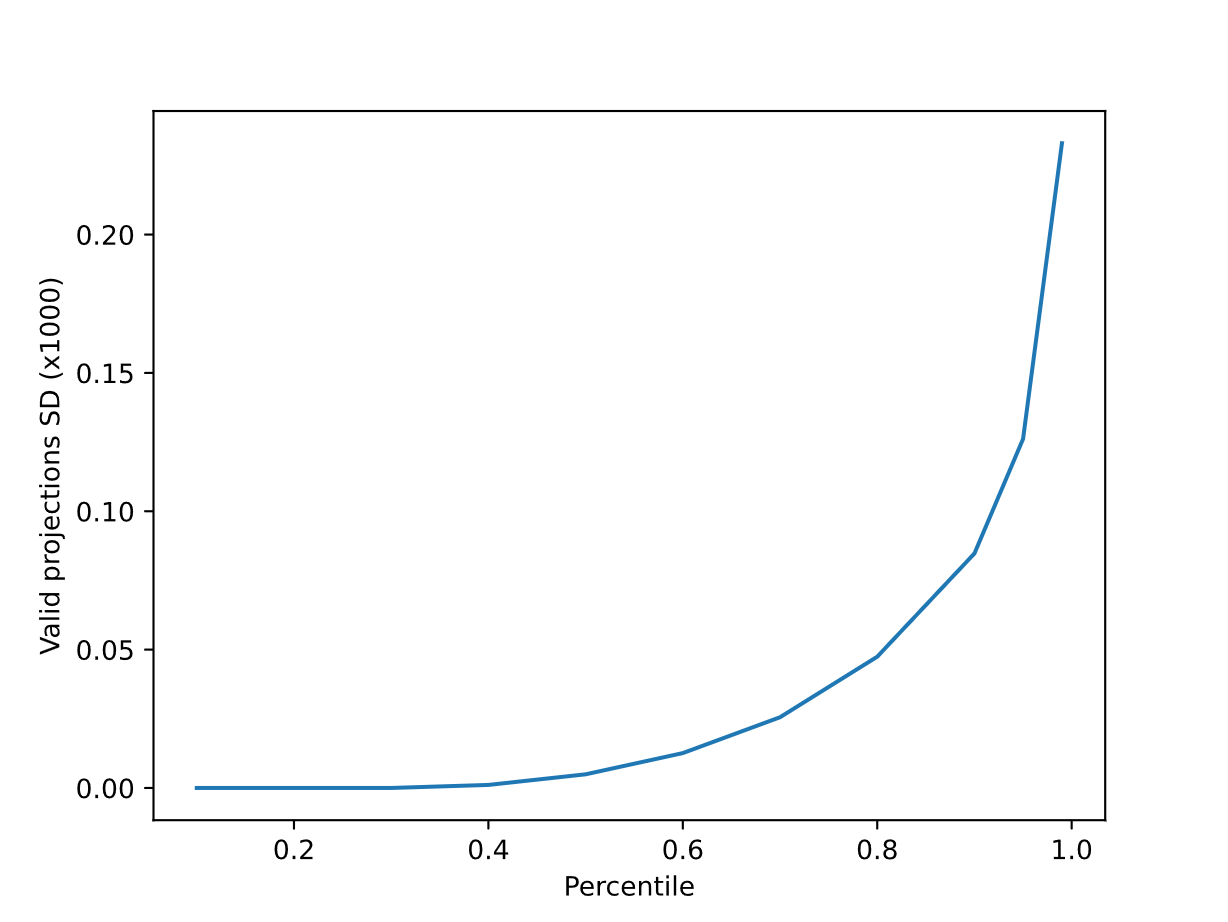

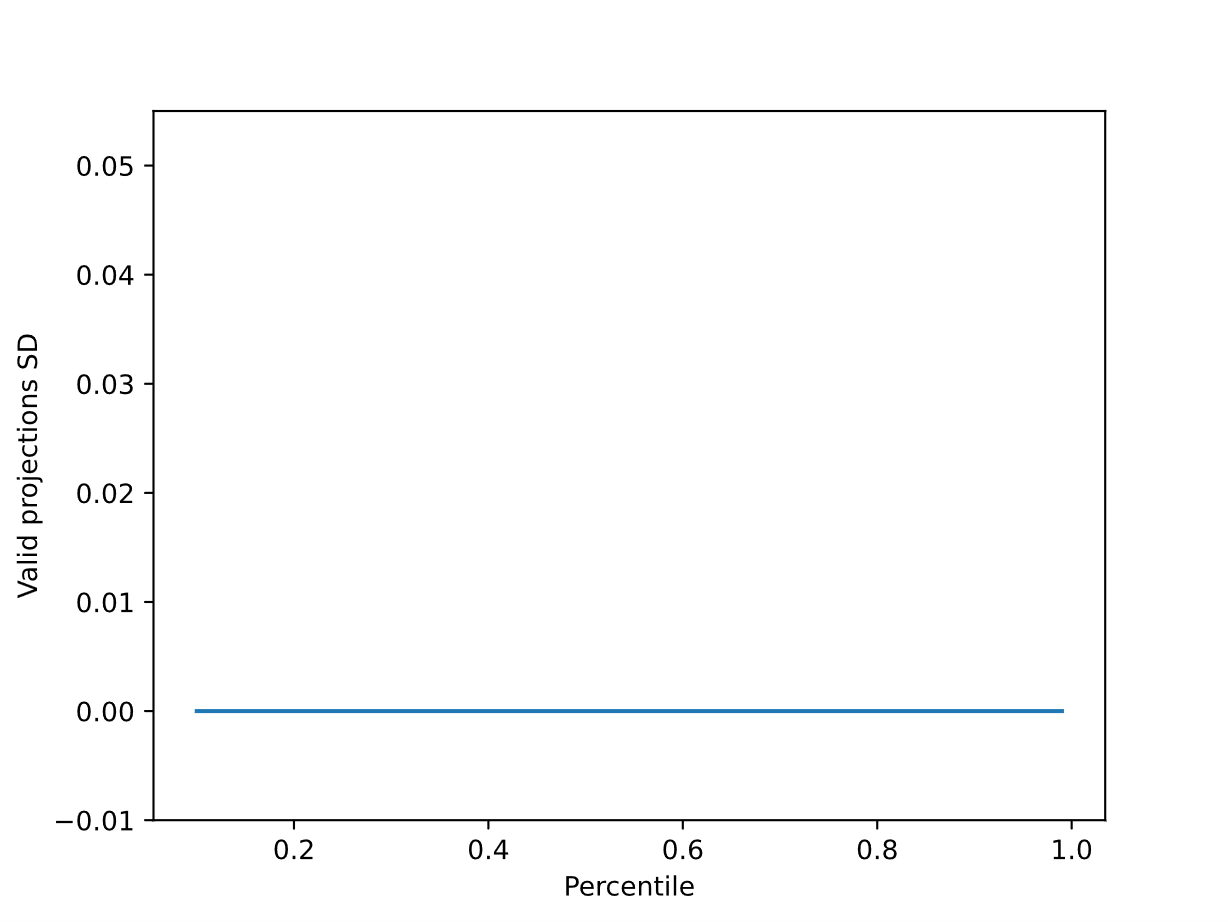

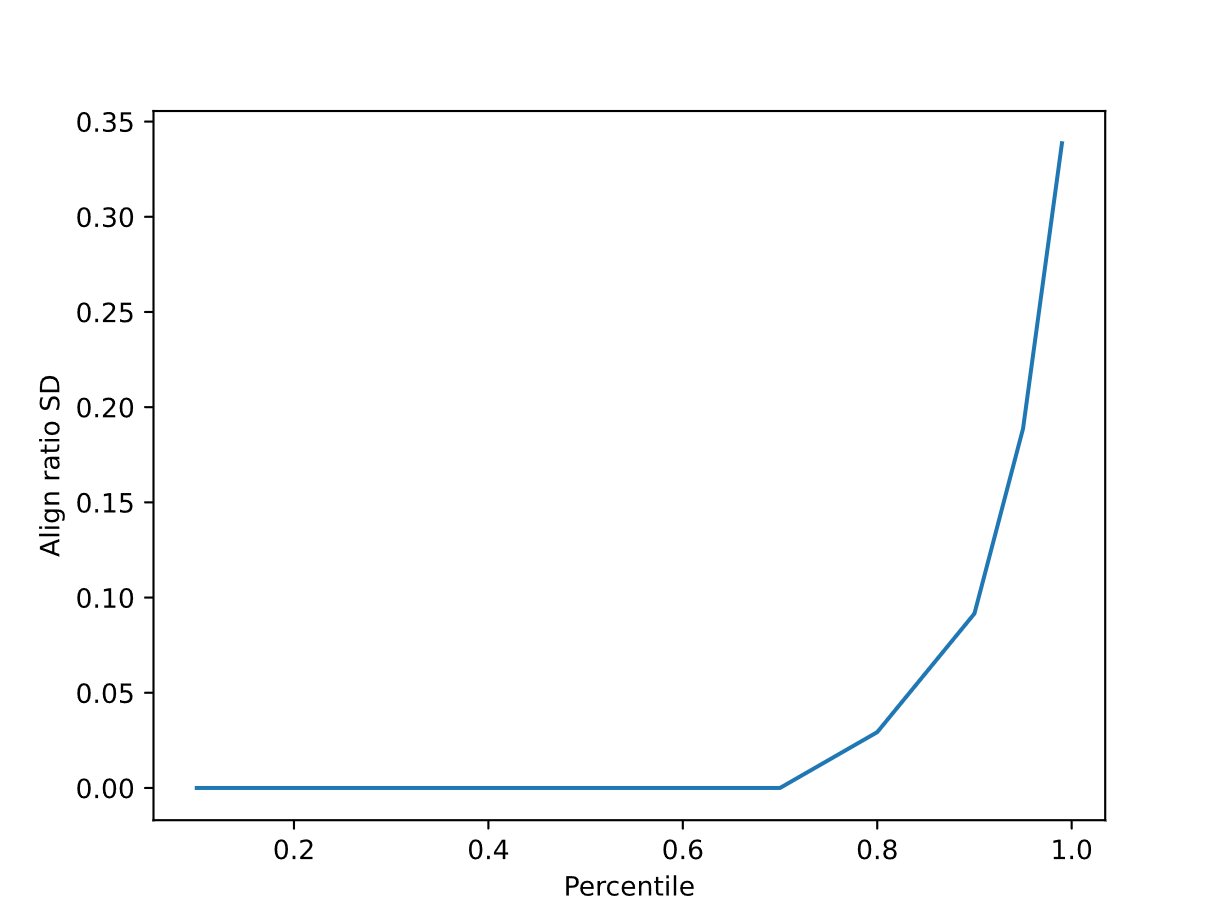

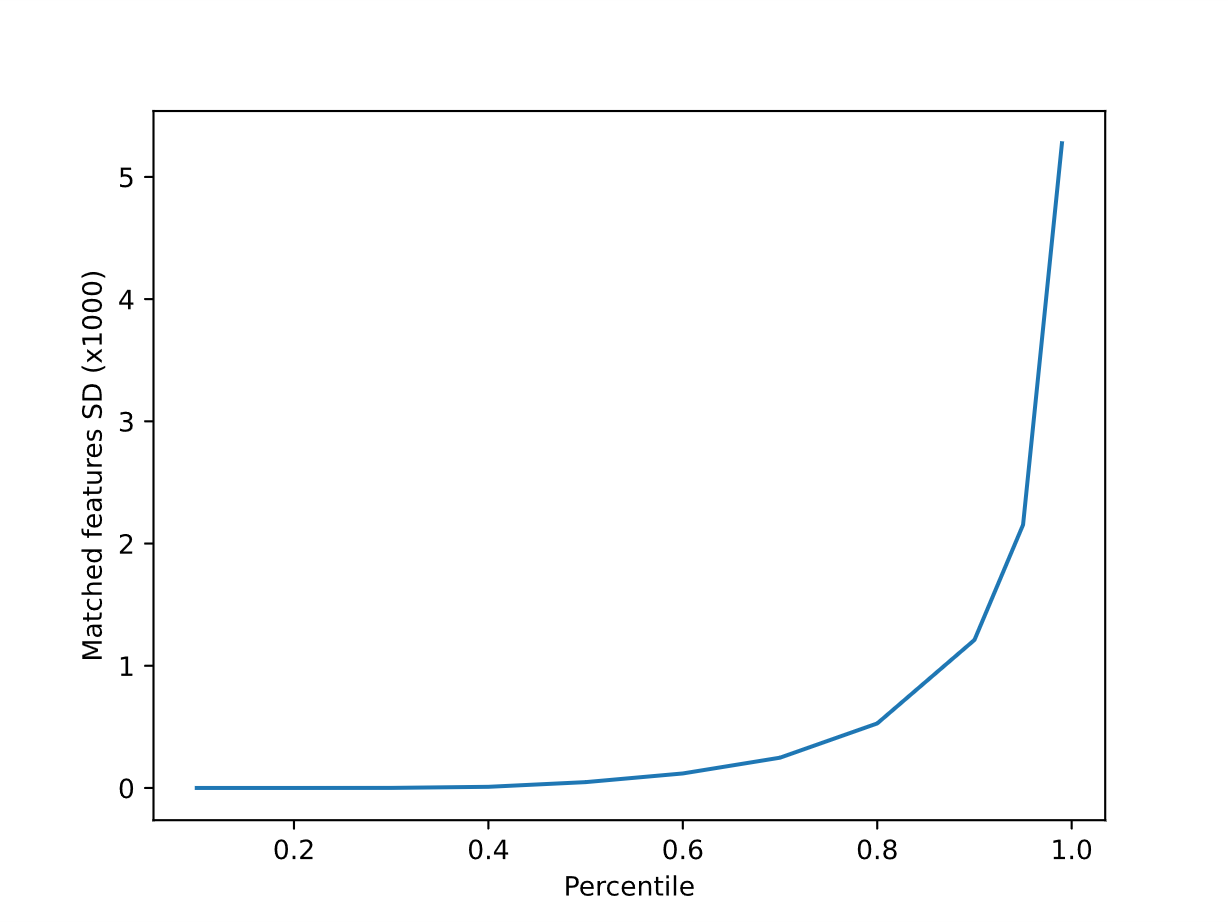


Fig. S58 SD of median of valid projections per photo. Noticeable variability emerged at the median (SD = 4.29 x 10^3^), increasing to 84.74 x 10^3^ at the 90th percentile and 232.99 x 10^3^ at the 99th percentile.

Fig. S57. SD of median valid projections per matched feature. All groups showed no variability (SD = 0)

Fig. S56 SD of align ratio. Noticeable variability emerged at the 80th percentile (SD = 0.029), increasing to 0.091 at the 90th percentile and 0.338 at the 99th percentile.

Fig. S55 SD of number of matched features. Noticeable variability emerged at the 40th percentile (SD = 9.345 x 10^3^), increasing to 1,211.08 x 10^3^ at the 90th percentile and 5,275.40 x 10^3^ at the 99th percentile.


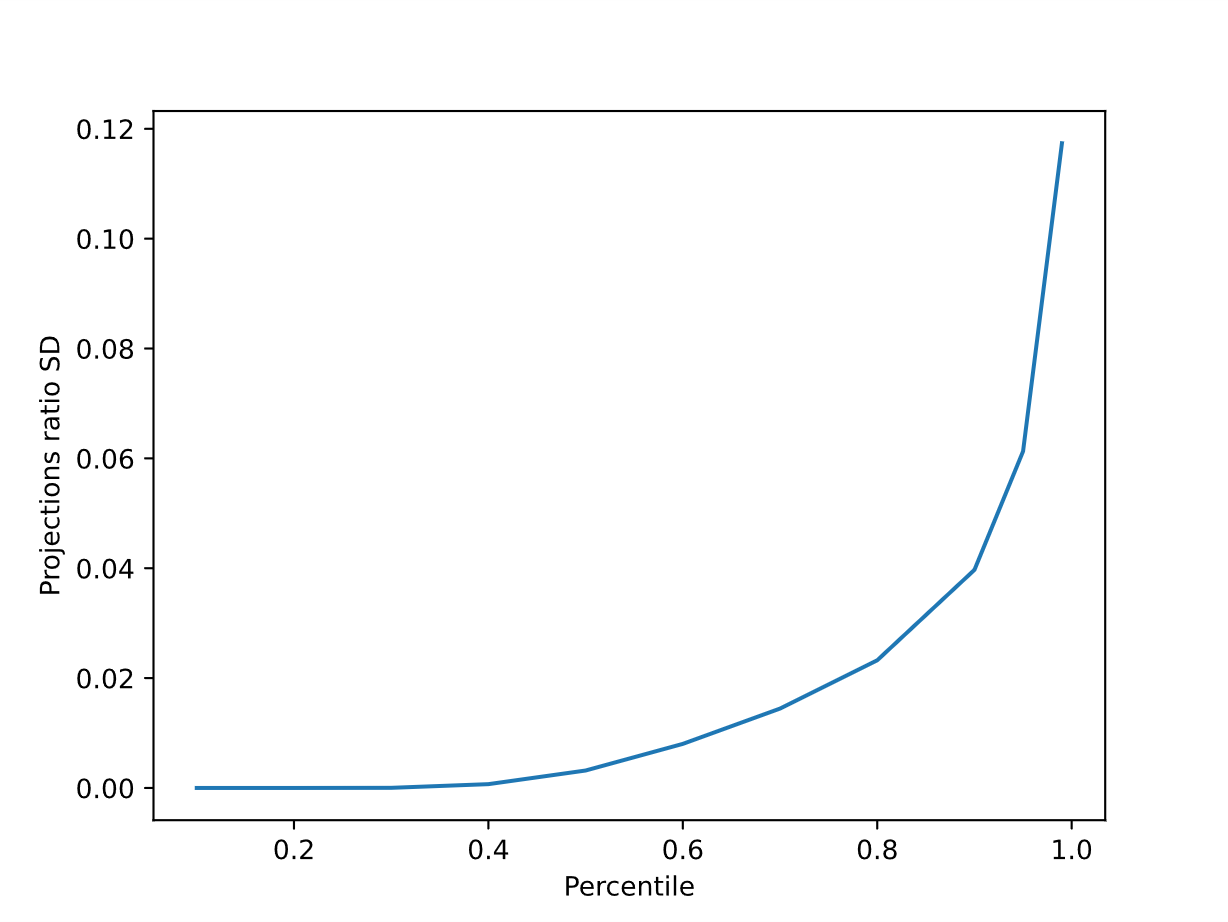

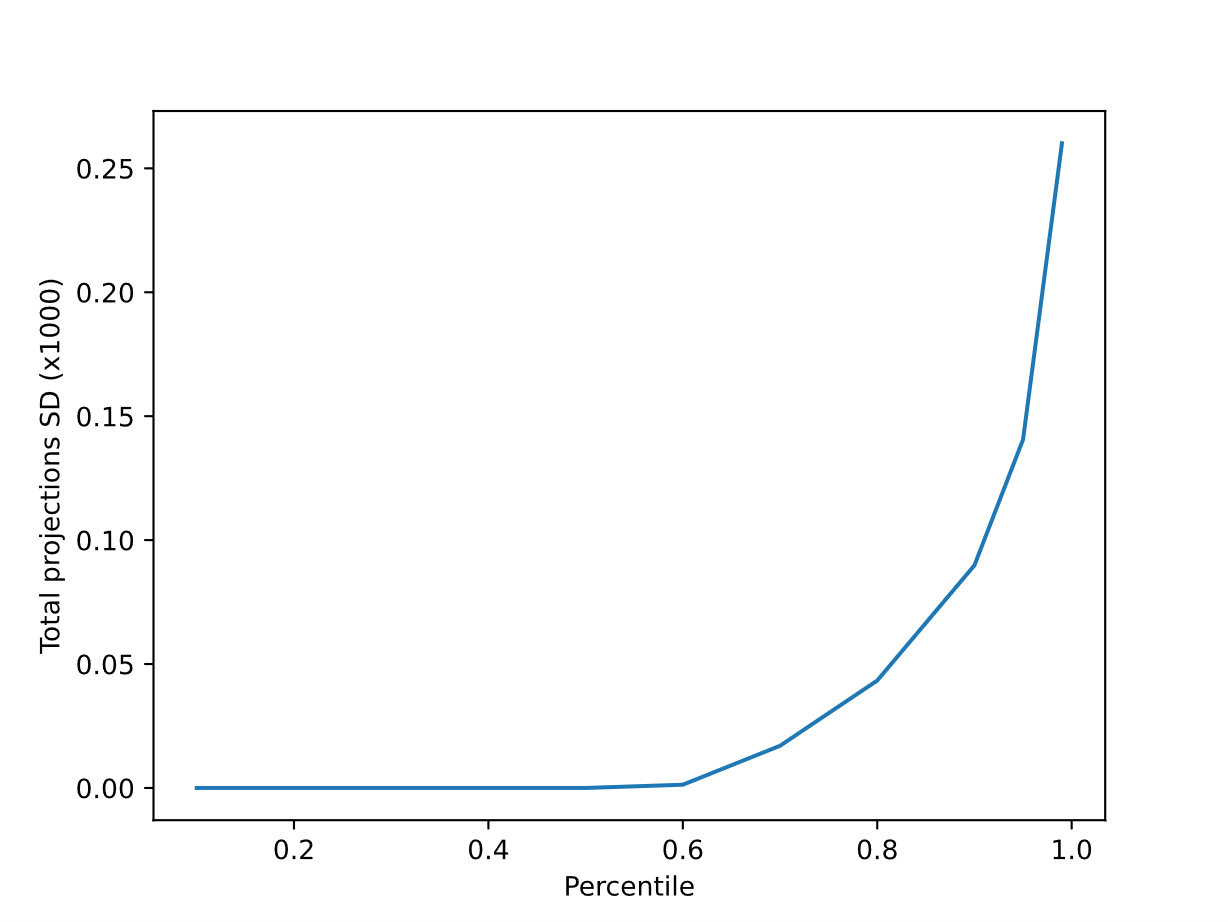


Fig. S60 SD of median of projection ration per photo. Noticeable variability emerged at the median (SD = 3.17 x 10^-3^), increasing to 39.70 x 10^-3^ at the 90th percentile and 117.36 x 10^-3^ at the 99th percentile.

Fig. S59 SD of median of total projections per photo. Noticeable variability emerged at the 60th (SD = 1.30 x 10^3^), increasing to 89.83 x 10^3^ at the 90th percentile and 260.12 x 10^3^ at the 99th percentile.


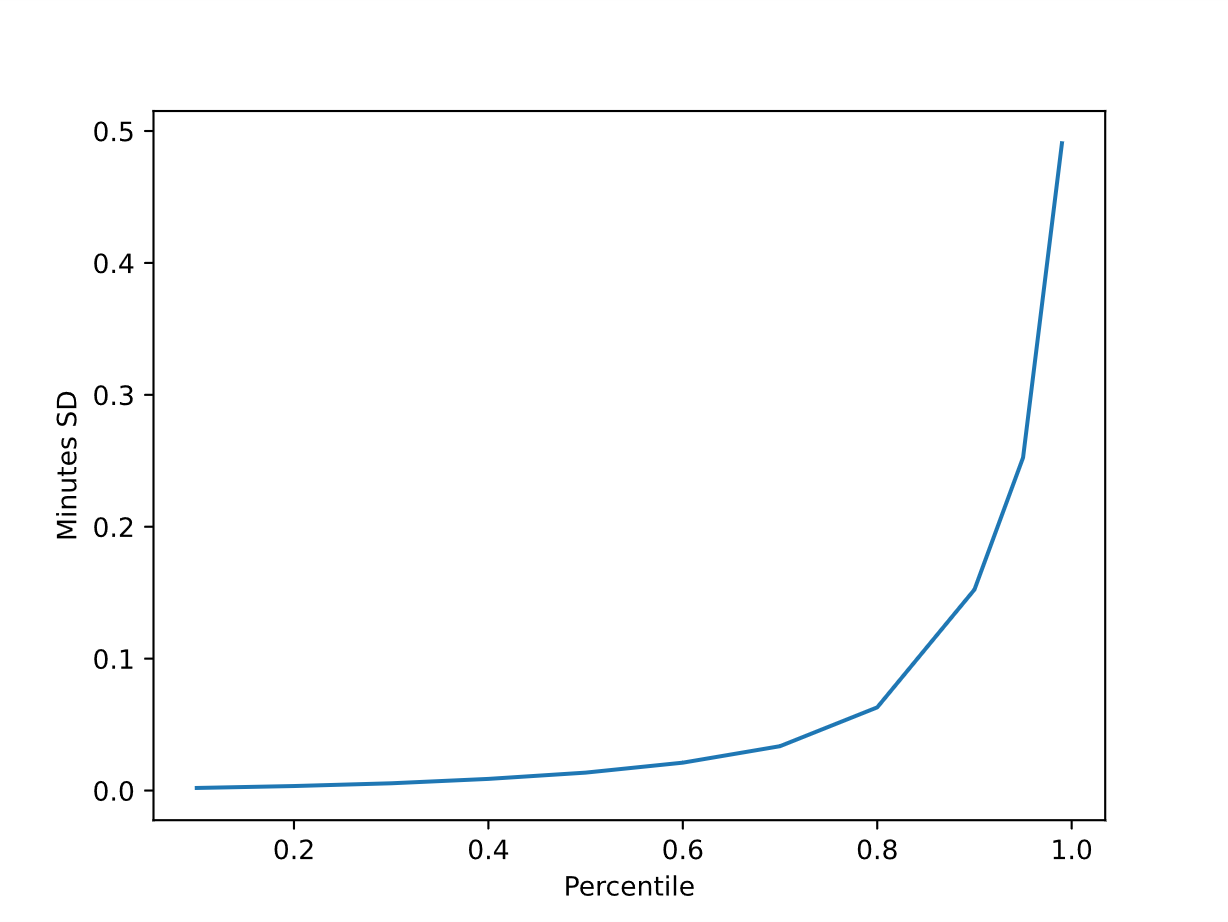


Fig. S61 SD of SfM total time. Noticeable variability emerged at the median (SD = 0.013 minutes), increasing to 0.152 minutes at the 90th percentile and 0.490 minutes at the 99th percentile.
